# Practice Facilitation and Peer Coaching for Uncontrolled Hypertension Among Black Individuals: A Randomized Clinical Trial

**DOI:** 10.1001/jamainternmed.2024.0047

**Published:** 2024-03-18

**Authors:** Monika M. Safford, Doyle M. Cummings, Jacqueline R. Halladay, James M. Shikany, Joshua Richman, Suzanne Oparil, James Hollenberg, Alyssa Adams, Muna Anabtawi, Lynn Andreae, Elizabeth Baquero, Joanna Bryan, Debra Sanders-Clark, Ethel Johnson, Erica Richman, Orysya Soroka, Jimmy Tillman, Andrea L. Cherrington

**Affiliations:** 1 Weill Medical College of Cornell University, New York, New York; 2 East Carolina University, Greenville, North Carolina; 3 University of North Carolina at Chapel Hill; 4 University of Alabama at Birmingham; 5 Health and Wellness Education Center of Livingston, Alabama; 6 West Central Alabama Community Health Improvement League of Camden; 7 Open Water Coaching and Consulting, Cape Carteret, North Carolina

## Abstract

**Importance:**

Rural Black participants need effective intervention to achieve better blood pressure (BP) control.

**Objective:**

Among Black rural adults with persistently uncontrolled hypertension attending primary care clinics, to determine whether peer coaching (PC), practice facilitation (PF), or both (PCPF) are superior to enhanced usual care (EUC) in improving BP control.

**Design, Setting, and Participants:**

A cluster randomized clinical trial was conducted in 69 rural primary care practices across Alabama and North Carolina between September 23, 2016, and September 26, 2019. The participating practices were randomized to 4 groups: PC plus EUC, PF plus EUC, PCPF plus EUC, and EUC alone. The baseline EUC approach included a laptop for each participating practice with hyperlinks to participant education on hypertension, a binder of practice tips, a poster showing an algorithm for stepped care to improve BP, and 25 home BP monitors. The trial was stopped on February 28, 2021, after final data collection. The study included Black participants with persistently uncontrolled hypertension. Data were analyzed from February 28, 2021, to December 13, 2022.

**Interventions:**

Practice facilitators helped practices implement at least 4 quality improvement projects designed to improve BP control throughout 1 year. Peer coaches delivered a structured program via telephone on hypertension self-management throughout 1 year.

**Main Outcomes and Measures:**

The primary outcome was the proportion of participants in each trial group with BP values of less than 140/90 mm Hg at 6 months and 12 months. The secondary outcome was a change in the systolic BP of participants at 6 months and 12 months.

**Results:**

A total of 69 practices were randomized, and 1209 participants’ data were included in the analysis. The mean (SD) age of participants was 58 (12) years, and 748 (62%) were women. In the intention-to-treat analyses, neither intervention alone nor in combination improved BP control or BP levels more than EUC (at 12 months, PF vs EUC odds ratio [OR], 0.94 [95% CI, 0.58-1.52]; PC vs EUC OR, 1.30 [95% CI, 0.83-2.04]; PCPF vs EUC OR, 1.02 [95% CI, 0.64-1.64]). In preplanned subgroup analyses, participants younger than 60 years in the PC and PCPF groups experienced a significant 5 mm Hg greater reduction in systolic BP than participants younger than 60 years in the EUC group at 12 months. Practicewide BP control estimates in PF groups suggested that BP control improved from 54% to 61%, a finding that was not observed in the trial’s participants.

**Conclusions and Relevance:**

The results of this cluster randomized clinical trial demonstrated that neither PC nor PF demonstrated a superior improvement in overall BP control compared with EUC. However, PC led to a significant reduction in systolic BP among younger adults.

**Trial Registration:**

ClinicalTrials.gov Identifier: NCT02866669

## Key Points

**Question:**

Does coaching by a trained peer or practice facilitation improve persistently uncontrolled high blood pressure in rural Black participants?

**Findings:**

In this cluster randomized clinical trial of 69 practices with 1209 participants, neither peer coaching nor practice facilitation improved blood pressure control better than enhanced usual care. Peer coaching significantly lowered systolic blood pressure in people younger than 60 years by 5 mm Hg.

**Meaning:**

Peer coaching can help younger rural Black individuals to lower their blood pressure.

## Teaser

This cluster randomized clinical trial evaluates the effectiveness of peer coaching, practice facilitation, or a combination of both approaches, compared with enhanced usual care, in improving blood pressure control among Black participants in the rural South.

## Introduction

Hypertension is common among Black individuals, resulting in high rates of cardiovascular disease and kidney failure and shortening life expectancy.^1,2^ In the rural Southeastern US, an economically depressed area with long-standing primary care shortages, more than one-half of Black adults have hypertension.^3,4,5^ Distances and high fuel costs make regular attendance at medical office visits difficult. Effective strategies to overcome these barriers and improve health outcomes for Black individuals with hypertension living in these areas are needed.

Two practical and scalable approaches to improving blood pressure (BP) hold promise. Peer coaching (PC) improves self-care for chronic diseases but has not been tested among Black hypertensive residents in this region.^6,7,8,9,10^ Practice facilitation (PF) is gaining traction to teach resource-constrained primary care practices quality improvement (QI) and population health management techniques.^11^ PF has not been rigorously tested to improve BP control, and neither intervention has been tested in this region specifically.

In collaboration with primary care practices and their surrounding communities, we conducted a cluster randomized clinical trial to test the hypothesis that PC or PF, alone or in combination (PCPF), results in better BP control and BP levels than enhanced usual care (EUC) among Black participants with persistently uncontrolled hypertension. The trial was designed to examine high-risk subgroups including individuals younger than 60 years, men, people with depressive symptoms, and those with low health literacy.

## Methods

### Trial Design and Oversight

In this cluster randomized clinical trial, the participating practices were randomized to 4 groups: PC plus EUC, PF plus EUC, PCPF plus EUC, and EUC alone (trial protocol in Supplement 1). Practicing stakeholder engagement, the research team adapted the trial protocol to accommodate individual practice settings, regularly seeking guidance from community advisory boards, as described elsewhere.^12^ We initially sought to engage 80 primary care practices and 25 Black participants attending each practice for a total of 2000 participants. However, natural disasters and unanticipated costs prompted reanalysis of the trial’s power that led the data and safety monitoring board to approve reducing the number of practices to 69 (eMethods 1 in Supplement 2). The study was conducted from June 2016 to February 2021. It was approved by the institutional review board of all participating institutions, and all participants provided written informed consent. Adherence to the Consolidated Standards of Reporting Trials (CONSORT) reporting guideline was ensured. Data were analyzed from February 28, 2021, to December 13, 2022.

### Setting and Participants

This randomized clinical trial was conducted in what is referred to as the *Black Belt* of Alabama and North Carolina, a rural region with high proportions of Black individuals and limited economic development. The Black Belt is not precisely defined geographically but reflects historical roots in the prevalence of enslaved people before the US Civil War. Primary care practices that were located in the Black Belt that had on-site internet access were eligible. Practice champions at each practice served as study contacts. Participants, who self-identified as Black, resided in the Black Belt. They ranged in age from 19 years to 85 years, spoke English, had telephone access, and demonstrated persistently uncontrolled hypertension, defined as mean systolic BP of at least 140 mm Hg in their medical record in the year preceding enrollment, plus systolic BP of at least 140 mm Hg or diastolic BP of at least 90 mm Hg on enrollment as assessed by a research assistant (RA) following a guideline-concordant protocol.^13^ In this community-partnered trial, community input resulted in selecting the target BP of 140 mm Hg rather than 130 mm Hg due to the large number of participants with BP above 140 mm Hg. The engagement of participants with persistently uncontrolled hypertension reflected community requests for help for those in greatest need. By design, the trial aimed to recruit one-half men, with one-half of participants younger than 60 years to ensure diversity of age and gender, which would bolster the prespecified subgroup analyses. Recruitment was conducted at practices, and written informed consent was obtained by research staff.

### Randomization and Blinding

The unit of randomization was by practice. The study statistician (J.R.) used permuted block randomization and a computerized random number generator with block sizes of 2 and 4 to minimize imbalance, balancing allocation across federally qualified health centers (FQHC) and non-FQHC practices by state. Data collectors, data analysts, and as many investigators as possible were blinded to group assignments. Because the interventions were behavioral, it was not possible to blind practices, some investigators, peer coaches, and practice facilitators to group assignments.

### Peer Coaching Intervention

Peer coaches were recruited from the community and trained to deliver a structured 8-session self-management intervention focused on dietary salt restriction and healthy eating guided by the DASH (Dietary Approaches to Stop Hypertension) diet; physical activity; self-monitoring BP; medication adherence; communication with the health care team; and stress reduction. The intervention’s theoretical foundations included motivational interviewing and goal setting, empowerment, adult learning theory, and social modeling.^14,15,16^ The intervention was delivered via telephone. When enrolled in the study, participants were teamed with a coach, and the intervention was initiated at the participant’s convenience as soon as possible. Coaches communicated progress to practices each month. After the initial 8 sessions taking place weekly, coaches maintained contact monthly for 1 year.

### Practice Facilitation Intervention

Practice facilitators were either experienced or completed training through the Millard Fillmore College Practice Facilitator Certificate Program.^17^ All practice facilitators received 1 half day of motivational interviewing training. PF was initiated when all participants were enrolled at the practice. Facilitators worked with practices throughout 1 year, making in-person visits at least monthly and communicating by telephone, email, or video conferencing between visits. Facilitators helped practices implement at least 1 hypertension-related QI project in each of 4 key areas theorized to be drivers of change: (1) using the practice’s data to monitor change; (2) team-based care; (3) standardized care processes; and (4) self-management support. Facilitators rated progress monthly using the Key Drivers of Implementation Scale, a validated qualitative assessment of progress toward implementing the Chronic Care Model.^18,19^ They assessed progress in the domains of standardized care processes (range, 1-4 points), clinical information systems (range, 1-3 points), self-management support (range, 1-5 points), optimized team care (range, 1-3 points), and practice leadership (range, 1-3 points). The maximum score of 18 points indicated high implementation.

### Enhanced Usual Care

All practices received EUC, consisting of a laptop computer with access to a participant education system called the Patient Activated Learning System,^20,21^ 25 home BP monitoring machines for the 25 individuals enrolled from that practice, a binder of practice tips, and a laminated poster depicting an algorithm for stepping up hypertensive medications tailored for Black participants.

### Outcomes and Data Collection

The primary outcome was BP control, defined as a BP reading of less than 140/90 mm Hg. The secondary outcome was a change in systolic BP level. BP was assessed by RAs in the practice at baseline, 6 months, and 12 months using a digital BP monitoring machine (OMRON Healthcare, Inc), which automatically assessed 3 readings that were averaged for analyses. RAs followed a quality-controlled protocol (ie, 5-minute seated rest with back support with both feet flat on the floor before BP measurement). Additional data were collected to assess mechanisms of how the interventions exerted their effects, including self-reported medication adherence; diet; physical activity; and characteristics that may influence intervention effectiveness, such as depressive symptoms, perceived stress, physical and mental functioning, and health literacy. Validated scales were used where available.^12^

### Statistical Analysis

The main hypothesis test was at the participant level, adjusting for baseline covariates with imbalance across treatment groups and seasons. Analyses used generalized longitudinal mixed models including both individual-level and practice-level random effects, controlling for false discovery of *P* < .05 using the Benjamini-Hochberg approach.^22^ All analyses followed an intention-to-treat approach. The secondary outcome of change in BP levels was also analyzed using generalized linear mixed models (GLMM), including a term reflecting days from baseline to follow-up. The use of GLMM with likelihood-based estimation allowed for observations at 6 months to inform estimates at 12 months through within-participant correlation. Prespecified subgroup analyses included age (<60 years vs ≥60 years), gender, depressive symptoms, and health literacy, using the same primary and secondary outcomes and GLMM approach.

All analyses accounted for the clustering of participants within practices and all participants were analyzed by trial group assignment. The sample size was determined based on the ability to detect at least a 15–percentage point difference in BP control between any of the 3 intervention groups and EUC with at least 80% power, assuming an intraclass correlation coefficient of .02. The actual intraclass correlation coefficient ranged from less than .01 to .02.

The post hoc analysis used the monthly practice-level BP control estimates generated as part of QI activities by PF practices. Practices recorded the number of hypertensive individuals regardless of race seen in the past month (denominators), and the number with a BP reading of less than 140/90 mm Hg (numerators). All numerators and denominators were combined into a dataset and mixed models derived predicted probabilities of BP control and 95% CIs over time while accounting for clustering of participants within practices and variation in denominators across practices. All analyses were conducted using SAS (SAS Institute) and STATA 16 (StataCorp) statistical software programs. Two-sided *P* values <.05 were considered statistically significant.

## Results

**Figure 1.**
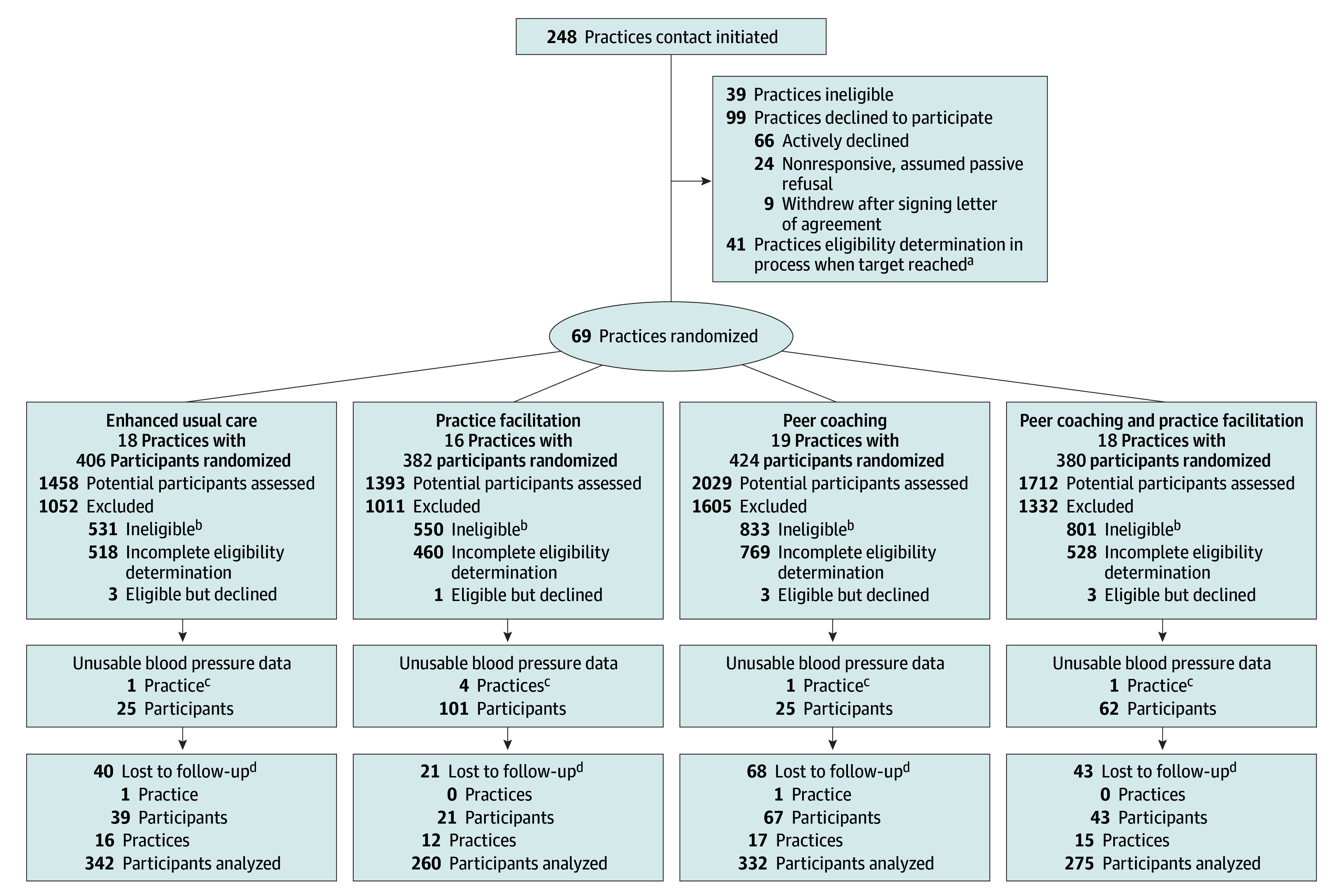
Consort Diagram ^a^Total number of practices modified after initiation of the study. The recruitment goal was 25 participants per practice; the design allowed for as few as 18 participants. ^b^Reasons for ineligibility of participants included age (n = 43), ineligible blood pressure at the time of enrollment (n = 1371), cancer (n = 32), cognitive impairment (n = 23), current illness (n = 1), deceased (n = 10), estimated glomerular filtration rate out of range (n = 142), unable/unwilling to work with peer coach (n = 36), moving/not local (n = 24), not an active participant at the clinic (n = 95), not willing to use own phone (n = 2), office did not recommend (n = 7), participating in another study (n = 9), race not self-identified as Black (n = 23), declined/not interested in screening (n = 892), unwilling to participate in study data collection (n = 27), uses a wheelchair (n = 14), language barrier (n = 4), pregnant (n = 8), and no reason listed (n = 7). ^c^Blood pressure data from participants at 7 practices were unusable; some participants at additional practices also had unusable blood pressure data. ^d^Two practices withdrew, 1 in the peer coaching group and 1 in the enhanced usual care group. The latter had 4 participants at the time of withdrawal who continued to be followed up and grouped with a nearby practice; thus, this practice’s participants were not lost to follow-up even though the practice closed. Practices lost to follow-up were missing both 6-month and 12-month follow-up data.

A total of 207 practices completed eligibility assessments, of which 69 were randomized between September 23, 2016, and September 26, 2019 (Figure 1). Nearly one-half were FQHCs or community clinics, all were well established, and all served a large proportion of participants enrolled in Medicare or Medicaid (eTable 1 in Supplement 2). Practices estimated that 20% of their patients were uninsured, and 20 (29%) had patient-centered medical home status.^23^

A total of 1592 participants were enrolled: 406 (26%) in the EUC group, 382 (24%) in the PF group, 424 (27%) in the PC group, and 380 (24%) in the PCPF group. Of these participants, 1336 individuals (84%) completed the 12-month follow-up, and 1209 participants had usable data (eMethods 2 in Supplement 2). The trial was stopped on February 28, 2021, after final data collection. The mean (SD) age of the participants was 58 (12) years, 748 (62%) were women, 268 (22%) had less than a high school education, 547 (45%) reported an annual household income of less than $20 000, and mean (SD) baseline BP was 156/90 (17/14) mm Hg (Table).^24,25,26^ Diabetes was present in 568 participants (47%), and 111 (9%) reported a history of stroke. Only 178 (15%) reported excellent to very good health. Additionally, 475 (39%) reported difficulty reading and understanding written information; 194 (16%) lacked health insurance; and 961 (81%) reported at least 1 barrier to medication adherence. Furthermore, 314 (26%) had visited an emergency department (ED), and 116 (10%) were hospitalized in the 6 months before enrollment. Participants were prescribed a mean (SD) of 3.7 (2.6) antihypertensive agents at baseline, and antihypertensive medications were intensified between baseline and follow-up at 12 months in 459 participants (38%).

**Table.** Participant Characteristics in Southeastern Collaboration to Improve Blood Pressure Trial, Stratified by Study Group

Characteristic	No. (%)			
	Enhanced usual care	Practice facilitation	Peer coaching	Peer coaching + practice facilitation
No.	342	260	332	275
Sociodemographic variable				
Age, mean (SD), y	59 (12)	56 (11)	58 (12)	57 (12)
Gender				
Men	136 (40)	107 (41)	127 (38)	91 (33)
Women	206 (60)	153 (59)	205 (62)	184 (67)
<High school education	81 (24)	63 (24)	64 (19)	60 (22)
<$20 000 Annual income	141 (41)	139 (54)	146 (44)	121 (44)
Married	137 (40)	89 (34)	110 (33)	99 (36)
Working^a^	251 (74)	186 (72)	241 (73)	180 (65)
Medical history				
Diabetes	160 (47)	123 (47)	161 (48)	124 (45)
Past stroke	28 (8)	27 (10)	32 (10)	24 (9)
Obesity	86 (25)	89 (34)	91 (27)	66 (24)
Depressive symptoms^b^	53 (16)	59 (23)	89 (27)	52 (19)
Current cigarette smoking	55 (16)	73 (28)	76 (23)	54 (20)
Self-rated health				
Excellent or very good health	51 (15)	32 (13)	48 (14)	47 (17)
Physical functioning score, mean (SD)^c^	41 (11)	40 (10)	41 (11)	41 (12)
Mental functioning score, mean (SD)^d^	50 (11)	47 (11)	47 (11)	49 (11)
Health literacy^e^				
Needs help with reading written materials from physicians	132 (39)	120 (46)	109 (33)	94 (34)
Cannot understand written information	123 (36)	132 (51)	121 (37)	99 (36)
Access to health care				
No health insurance	51 (15)	56 (22)	52 (16)	35 (13)
≥1 Barrier to medication adherence^f^	262 (78)	212 (82)	262 (81)	225 (84)
Visited an emergency department in past 6 mo	88 (26)	66 (26)	94 (28)	66 (24)
Hospitalized in past 6 mo	28 (8)	26 (10)	44 (13)	18 (6)
Medication nonadherence	201 (60)	180 (69)	210 (65)	161 (60)
Blood pressure at baseline, mean (SD), mm Hg				
Systolic	158 (18)	156 (17)	153 (14)	157 (17)
Diastolic	90 (14)	90 (14)	89 (13)	91 (14)

^a^ Includes full-time or part-time work.

^b^ Personal Health Questionnaire score of at least 10, indicating moderate to severe depression.^23^

^c^ Physical Component Summary score of the Short Form 12-item survey.^24^

^d^ Mental Component Summary score of the Short Form 12-item survey.^24^

^e^ Participant reported needing help or inability to understand at least a little of the time.

^f^ From the Murage-Marrero-Monahan Medication Barriers scale.^25^

Training was provided to 82 peer coaches and 5 facilitators. Of 804 participants randomized to the PC group, 484 individuals (60%) completed all core intervention components. Each coach controlled their workload with a mean of 4 participants at any time (range, 1-9 participants). Participants reported high satisfaction with their coaches, and 417 individuals (97%) recommended peer coaches for others in their community with hypertension (eTable 2 in Supplement 2).

All 32 practices randomized to the PF group completed the intervention and implemented at least 1 QI activity in each of the required domains (see eMethods 3 in Supplement 2 for examples of QI activities). The facilitator caseload averaged 2 practices at any given time (monthly range, 1-7 practices). Practice champions expressed high satisfaction with their facilitators and rated in-person interactions higher than telephone or video conferencing encounters (eTable 3 in Supplement 2).

**Figure 2.**
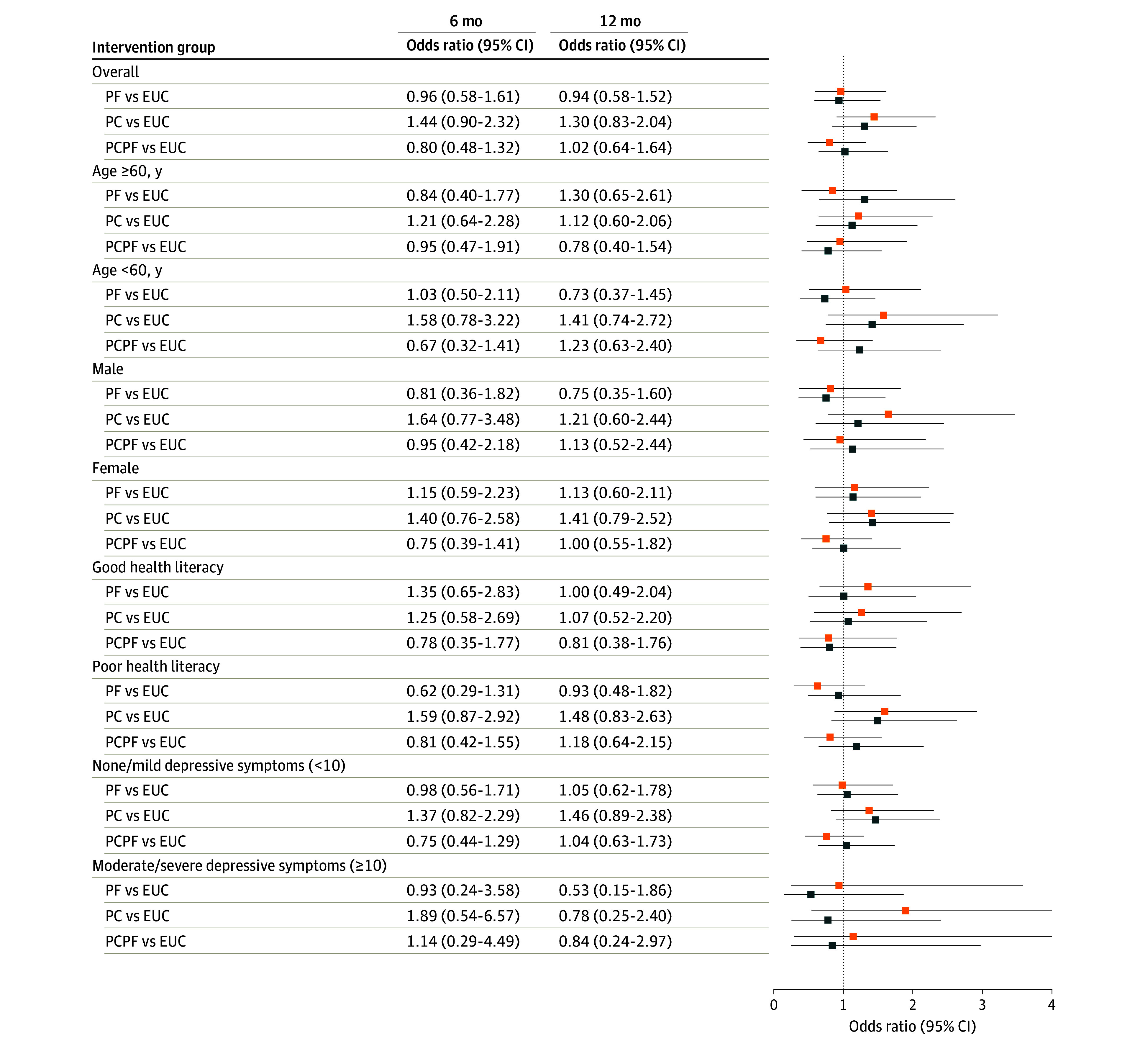
Odds Ratios for Blood Pressure Control by Trial Group and Subgroups The orange squares represent the 6-month values, and the blue squares reflect the values at 12 months. The error bars represent 95% CIs. EUC indicates enhanced usual care; PC, peer coaching; PF, practice facilitation; and PCPF, peer coaching plus practice facilitation.

**Figure 3.**
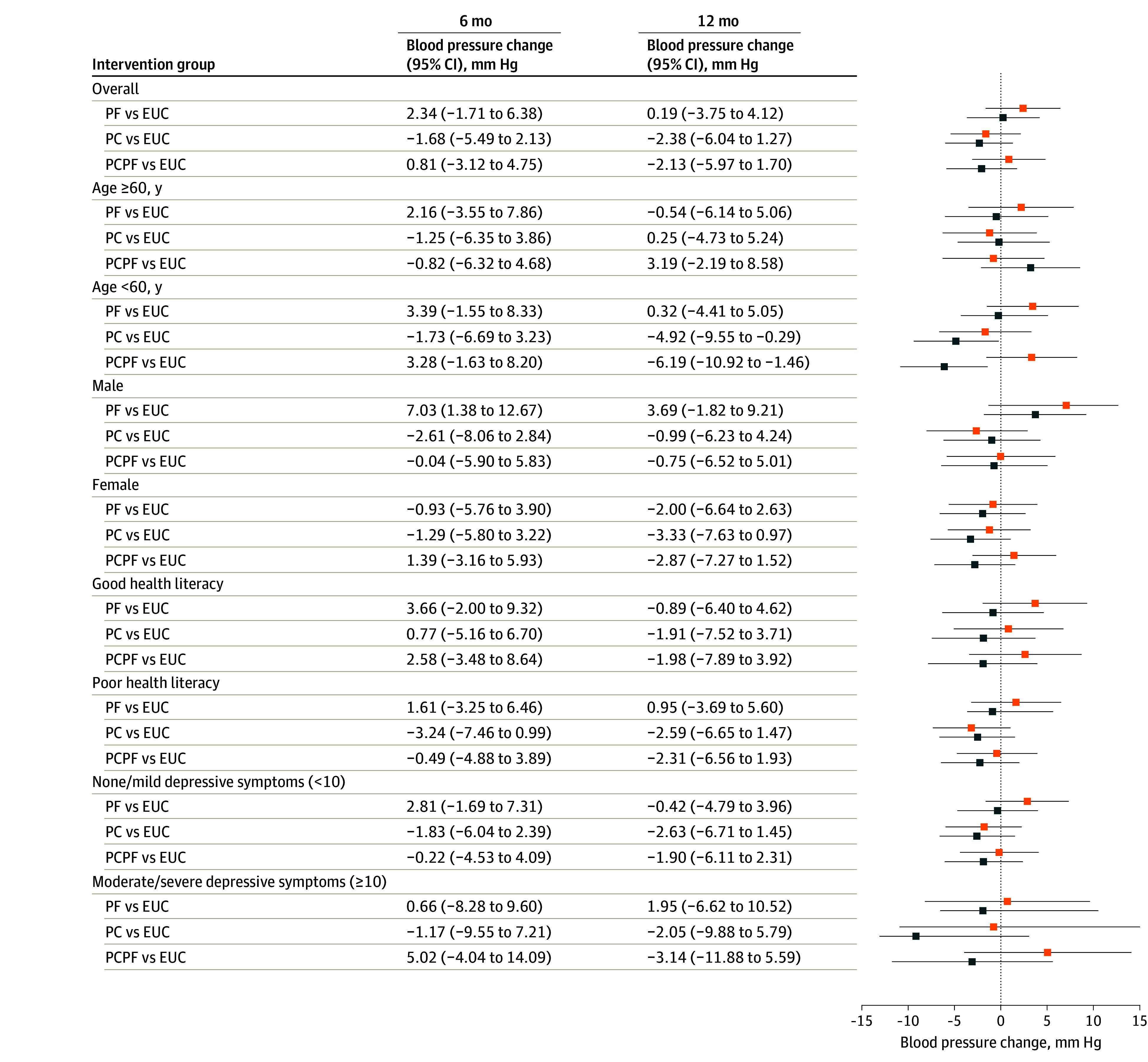
Systolic Blood Pressure Changes From Baseline to Follow-Up Overall and by Subgroups The orange squares represent the 6-month values, and the blue squares reflect the values at 12 months. The error bars represent 95% CIs. EUC indicates enhanced usual care; PC, peer coaching; PF, practice facilitation; and PCPF, peer coaching plus practice facilitation.

No intervention group achieved significantly better BP control or reduction in systolic BP level than the EUC-alone group, either overall or by any prespecified subgroups (Figures 2 and 3). At 12 months, 459 (38%) participants had controlled BP (123 [36%] for EUC, 94 [36%] for PF, 139 [42%] for PC, 99 [36%] for PCPF; *P* = .35). Men in the PF group experienced a significant 7.03 mm Hg rise in their systolic BP at 6 months, but this difference diminished and became nonsignificant at 12 months. However, among individuals younger than 60 years, systolic BP was significantly lowered (PC by 4.92 mm Hg; *P* = .04; PCPF by 6.19 mm Hg; *P* = .01).

**Figure 4.**
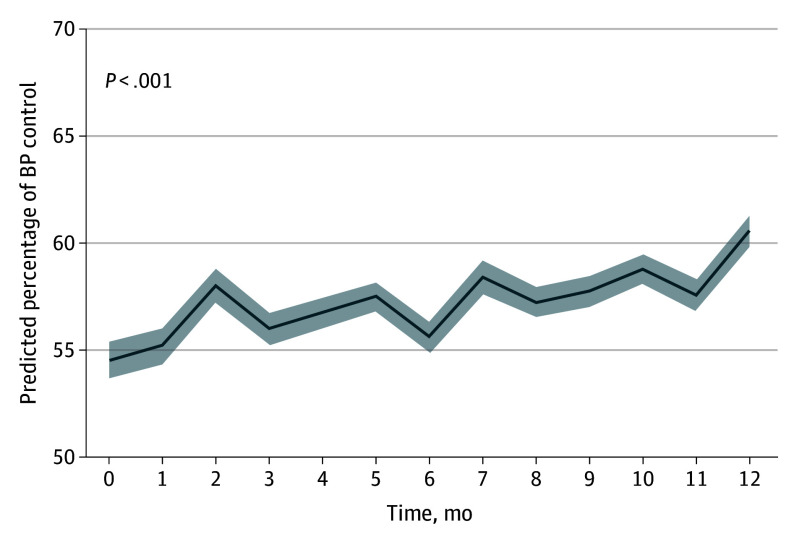
Monthly Self-Assessed Blood Pressure (BP) Control in Practice Facilitation Practices The graph represents aggregated monthly self-estimated percentages of BP control across all practice facilitation practices, presented as predicted percentages and their 95% CIs (gray shaded area). The self-assessed BP control values increased from 55% to 61% throughout the year, which was statistically significant (*P* < .001).

Among participants younger than 60 years in the PC group, compared with those randomized to EUC, a greater proportion improved their diet (68 [36%] vs 21 [21%], *P* = .01) and medication adherence (126 [43%] vs 46 [33%]; *P* = .04). Medication nonadherence in this younger group was reported by 214 participants (72%) at baseline and by 169 participants (57%) at follow-up; among those 60 years and older, baseline nonadherence was reported by 197 participants (51%). The self-assessed practicewide BP control estimates among PF practices rose from 55% to 61% throughout the year (*P* < .001) (Figure 4), while average Key Driver Implementation Scale scores rose from 3 points to 13.5 points (*P* < .001).

Safety end points included ED visits and hospitalizations. Overall, 279 (24.0%) of participants experienced an ED visit during months 0 to 6, and 255 (22.4%) experienced an ED visit during months 7 through 12. No trial group had significantly different ED visit frequency compared with the EUC group. Overall, 118 participants (10.2%) were hospitalized during months 0 through 6 with no significant differences across trial groups. Between months 7 and 12, 119 participants (10.4%) were hospitalized with 35 participants in the PF group (14.4%) compared with 23 participants in the EUC group (7.2%) (odds ratio, 2.19 [95% CI, 1.17-4.10]). No other trial group had significantly different hospitalizations vs EUC.

## Discussion

In this large cluster randomized clinical trial, neither PC nor PF resulted in better BP control in Black participants with persistently uncontrolled hypertension. However, in prespecified subgroup analyses, PC resulted in clinically important improvements in systolic BP for participants younger than 60 years. Self-assessed practice-level BP control rates at PF practices improved by 6 percentage points, suggesting that these practices may have made practicewide care improvements that did not reach the small sample of trial participants.

This randomized clinical trial adds to a growing foundation of evidence for effective strategies to lower BP in underresourced clinical settings that serve historically marginalized populations. The lack of overall findings underscores the challenges that primary care practice staff in this region face in helping their participants achieve better BP control. Although the overall trial results were not statistically significant, PC did offer clinically important benefits to participants younger than 60 years. The magnitude of systolic BP lowering in this subgroup was similar to that expected from low-dose thiazide diuretic drugs.^27^ PC has been shown to help participants improve their risk profiles in a variety of chronic diseases, but similar to past findings, PC was not effective for all participants. Moskowitz et al^28^ found that coaching worked best in participants with diabetes with low baseline adherence; we also found benefits among those with low medication adherence, in this case, for participants younger than 60 years. PC probably did not appeal to all participants in the PC intervention group because only 60% completed the entire intervention.

This clinical trial also provides preliminary evidence of the effectiveness of PF. Although trial participants demonstrated no benefit from PF, estimates of practice-level BP control suggested substantial gains throughout the yearlong intervention. During the planning of this trial, the proposal to implement at least 4 QI activities over a single year raised feasibility concerns with some of our partnering organizations. This randomized clinical trial demonstrates that this goal was not only feasible, it also may have resulted in clinically important improvements in practice-level control, even in practices with substantial resource constraints like those engaged in this study. Unfortunately, the practices’ lack of familiarity with using their electronic health record data did not allow for a more rigorous evaluation of practice-level effects by comparing changes in the PF group with practices not in the PF group. Nevertheless, the success of facilitators in improving BP control in underresourced primary care practices that served an impoverished population with 1 in 5 participants lacking health insurance was noteworthy.

One notable finding in this study was the 38% rate of medication intensification in trial participants. Superficially, this rate appears low, consistent with what is referred to as *clinical inertia*. However, this trial may effectively demonstrate the realities facing not only the trial participants but also their clinicians: Participants were already prescribed nearly 4 antihypertensive medications at baseline, with 4 of 5 reporting barriers to medication adherence.^29^ Although low-cost antihypertensive medications could theoretically have supported greater medication titration to achieve better BP control, hypertension was rarely the only chronic medical condition. Participants have reasonable concerns about adverse effects and medication interactions; furthermore, the harms of polypharmacy are well described.^30^ We, and others, have pointed out that much apparent clinical inertia may in fact be appropriate care.^31^ The finding of a modest but clinically relevant reduction of systolic BP with PC in younger adults without adding medication burden should be viewed through this lens and may suggest the need for tailored behavioral interventions that specifically target medication nonadherence rather than prescribing additional medications.

### Strengths and Limitations

Strengths of this study include its rigorous design and stakeholder-engaged philosophy, assuring feasibility and uptake, engagement of community members as change agents, and collaboration with community-based organizations to allow for scaling up and sustainability. These results may apply to other resource-constrained clinical practice environments serving low-income populations without high education levels. We focused on a rarely studied population in urgent need of intervention, namely, Black participants with persistently uncontrolled hypertension, a strength that also heightened the risk of regression to the mean, given known variability in BP levels. The 40% control at follow-up likely reflects regression to the mean, as well as improved adherence due to enrollment in a study, which is a well-described phenomenon.^32,33,34^ Given its purely educational content, EUC likely did not contribute substantially to achieving control.

Limitations of this study included demands of this study type resulting in excess costs that required midtrial modification of the study design, a practice not without precedent.^35^ The caseload for facilitators and coaches was relatively low in the present study, and effects may differ with larger caseloads. Nearly all practices in the PF groups chose to improve the standardization of BP assessment. Past studies suggest that clinic BP measures are on average 8 mm Hg higher than for clinics following guidelines.^36^ Thus, some observed improvements in practice-level BP control estimates could be attributable to improved measurement rather than true improved control.

## Conclusions

In this cluster randomized clinical trial, PC and PF did not improve either BP control or BP levels in rural Black participants overall. However, PC lowered BP among younger individuals despite variable engagement in the PC intervention. PF may hold promise for improving BP control at the practice level. This trial contributes to the evidence for both PC and PF to improve BP in populations who are difficult to access and at higher risk of poor outcomes.

## Notes

Supplement 1.
Trial Protocol

Supplement 2. **eMethods 1.** Details on the decision to reduce the trial’s sample size **eMethods 2.** Additional details on loss to follow-up **eMethods 3.** Examples of quality improvement activities **eTable 1.** Practice characteristics **eTable 2.** Perceived usefulness of Peer Coaches **eTable 3.** Practice Champions’ ratings of Practice Facilitators

Supplement 3.
Data Sharing Statement
