# Supplementary material for: Practice Facilitation and Peer Coaching for Uncontrolled Hypertension Among Black Individuals: A Randomized Clinical Trial
Source: JAMA Intern Med. 2024 Mar 18;184(5):538–46. doi: 10.1001/jamainternmed.2024.0047 (PMC10949149; doi:10.1001/jamainternmed.2024.0047)
Supplement: Supplement 1. — Trial Protocol [file jamainternmed-e240047-s001.pdf]

Southeastern Collaboration to Improve Blood Pressure Control

A collaborative project between:

University of Alabama at Birmingham;
Weill Cornell Medical College of Cornell University;
University of Carolina at Chapel Hill;
East Carolina University;
North Carolina Area Health Education Centers;
Alabama Area Health Education Centers;
Health and Wellness Education Center of Livingston, AL;
West Central Alabama Community Health Improvement League of Camden, AL;
ConnectionHealth, Birmingham, AL;
Open Water Coaching and Consulting, Cape Carteret, NC;
and 80 primary care practices,
peer coaches,
and practice facilitators living in the Black Belt region

Funded by NHLBI
UH3 HL130691-05

Study Protocol

Version November 1, 2021
NCT02866669
Unique Protocol ID: X160722009

1. Background and Significance

The background and significance section responds to **PCORI Methodology Standard 1:RQ-1** "Gap analysis to support the need for the proposed study".[1]

1.1 African Americans, Hypertension, and the Region known as the 'Black Belt'.

African Americans have among the highest prevalence of hypertension (HTN) in the world.[2] Figure 1 shows the areas of the US where HTN affects ≥60% of the US population, based on the 30,239-member REasons for Geographic And Racial Differences in Stroke (REGARDS) study which recruited African American and white subjects from the contiguous 48 US states.[3] As can be seen, the regions with the highest prevalence are in the Southeast, overlapping greatly with the Black Belt (Figure 2). The Black Belt is an agricultural rural region known for its steep poverty and largely African American population, and was described by Booker T. Washington as "*a part of the country which was distinguished by the colour of the soil. The part of the country possessing this thick, dark, and naturally rich soil was, of course, the part of the South where the slaves were most profitable, and consequently they were taken there in the largest numbers. Later and especially since the (Civil) war, the term seems to be used wholly in a political sense—that is, to designate the counties where the black people outnumber the white.*"[4] The Black Belt is therefore a region that has all three of the priority populations identified by the request for applications for this grant opportunity: rural, low-income, and minority, a 'triple threat' to health and longevity.

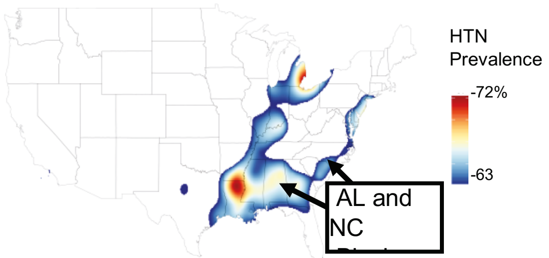

Figure 1. Areas of the US where HTN affects ≥60% of black and white adults age ≥45. Used by permission, Matthew Loop dissertation.

1.1.a Consequences of HTN in African Americans.

After a meteoric rise throughout the mid-20th century, cardiovascular disease (CVD) mortality plateaued and has had a steady decline since the 1970's.[2] However, not all race and sex groups have experienced the same pace of improvement; racial disparities in CVD persist in the 21st century,[5,6] with one of the most stubborn disparities related to HTN control.[7,8] Although African Americans are now consistently more likely to be aware of their HTN and also more likely to be treated, they continue to achieve suboptimal control.[8] In the 2003-2010 National Health and Nutrition Examination Survey (NHANES), 57.0% of African American participants with HTN were uncontrolled.[9] HTN-related outcomes that are more prevalent in African Americans than white Americans include stroke, end-stage renal disease, heart failure, death at presentation with coronary heart disease (CHD), as well as premature mortality.[2,5] Residents of the Black Belt have about the same life expectancy as Sri Lankans, and some counties have lower life expectancy than Malaysia, Ecuador or the Gaza Strip.[10] African Americans continue to be at least 50% more likely to die of heart disease or stroke prematurely (i.e., <75 years of age) than White Americans.[11]

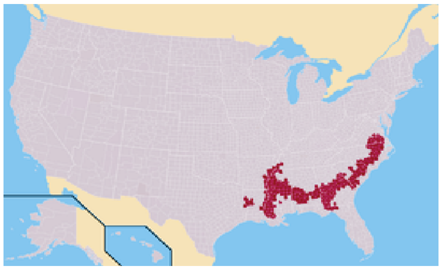

Figure 2. The US Black Belt.

In accordance with Patient-Centered Outreach Research Institute (**PCORI) Methodology Standard 1:RQ-3,** which focuses on identifying "specific populations and health decision(s) affected by the research to produce information that is meaningful and useful to people when making specific health decisions, research proposals, and protocols,"[1] this study will target African American participants.

1.1.b Healthcare in the Black Belt.

Today, the Black Belt includes some of the most disadvantaged counties in the Nation, with poverty commonly affecting a third or more of the residents, and with less availability of healthcare services than in more populated areas. Availability of primary care is lower than national averages, with the Kaiser Family Foundation estimating 3-8 primary care physicians/10,000 population in the Black Belt, compared with 25 nationally.[12] Area residents face significant additional challenges in curbing their risks for poor health outcomes. Poverty and low educational attainment have been reported to be associated with lack of risk factor control, both very common in the Black Belt. The 2009-2013 US Census data indicate that of the 30-40% of Black Belt residents living below the poverty line, only 1-3% had a bachelor's degree or higher, compared with 11-12% of individuals living in poverty nationally.[13] Distances are great, with many people traveling an hour or more to see the doctor, resulting in fewer visits and less monitoring of risk factors such as blood pressure (BP). In the NHANES 2003-10, visit frequency was strongly correlated with BP control (47.8% uncontrolled for those seen ≥2 times in the past year, 68.0% uncontrolled for those seen once, and 93.3% uncontrolled for those never seen). HTN control in Federally Qualified Health Centers (FQHC) in AL and NC Black Belt counties is shown in Table 1. As can be seen, FQHCs in both the AL and NC Black Belt served greater proportions of African Americans than their statewide counterparts, and they also achieved lower HTN control compared with others in their respective states, as well as nationally. Similar data are not available for private practices, but national data suggest that their risk factor control rates tend to fall below those of FQHCs.[9] Of note is the larger number of FQHC's in NC relative to AL, demonstrating variability in models of care available for Black Belt residents in the two states.

Table 1. Characteristics of patients in Black Belt Federally Qualified Health Centers in NC and AL.

	Total Patients	% Black	<100% Poverty	Hypertension	BP Control*
Alabama Grantees	330,401	48.1%	70.7%	29.4%	56.6%
Franklin Primary Health Center, Inc.	34,363	69.0%	80.6%	36.9%	55.7%
Health Services, Inc.	37,589	83.8%	85.9%	30.6%	51.4%
Rural Health Medical Program, Inc.	5,837	93.9%	77.1%	44.3%	48.9%
S.E. Alabama Rural Health Associates	71,617	30.7%	34.2%	34.5%	53.3%
Tri-County Medical Center	8,334	43.9%	75.3%	32.5%	28.1%
Whatley Health Services, Inc.	23,672	62.4%	85.2%	41.3%	52.9%
**Alabama Black Belt**	**181,412**	**64.0%**	**73.1%**	**36.7%**	**48.4%**
NC grantees	454,675	39.2%	74.1%	36.2%	60.8%
Anson Regional Medical Services	4,001	56.9%	44.3%	47.1%	47.5%
Carolina Family Health Centers, Inc.	14,490	43.1%	76.1%	53.5%	48.6%
First Choice Community Health Centers	11,085	36.7%	79.3%	22.1%	56.1%
Goshen Medical Center, Inc.	38,760	37.8%	51.1%	37.6%	57.9%
Kinston Community Health Center, Inc.	9,227	62.3%	75.1%	26.6%	47.0%
Opportunities Industrialization Center, Inc.	16,785	48.2%	80.3%	61.3%	87.4%
Robeson Health Care Corporation	12,637	29.7%	72.3%	35.3%	49.4%
Rural Health Group, Inc.	28,368	62.0%	66.1%	58.5%	63.6%
Stedman-Wade Health Services, Inc.	4,111	40.3%	41.4%	55.2%	74.1%
Tri County Community Health Council, Inc.	20,857	26.0%	74.3%	19.8%	56.7%
**NC Black Belt**	**160,321**	**44.3%**	**66.0%**	**41.7%**	**58.8%**
National Grantees	21,726,965	23.8%	71.9%	23.6%	63.6%

*Among patients with hypertension, the percentage with last blood pressure <140/90 mm Hg. Data from HRSA Primary Care: The Health Center Program (http://bphc.hrsa.gov/healthcenterdatastatistics/reporting/index.html).

1.2 Literature Review and Scientific Justification for the Study

This proposal advances the science through innovation in the following ways:

1. Black Belt populations have great needs in terms of healthcare innovations, but they are rarely studied.

2. To our knowledge, practice facilitation and peer coaching to improve (HTN), the two interventions to be tested here, have not been compared, especially not in rural underserved communities.

3. The study is designed from the beginning for scale-up throughout the Black Belt region, from Texas to Maryland. We accomplish this by situating the practice facilitators and peer coaches who will deliver the interventions in community-based organizations in AL, with plans to explore a similar community-based home in NC. The active involvement on our Community Advisory Boards (CABs) of health insurers and the State Departments of Health further strengthen the goal of designing a roadmap for other Black Belt communities, should our interventions prove effective and warrant wider scaling.

4. This study is designed as a pragmatic trial, with additional detail provided in section 3.2.

5. We advance methods of community-engaged research for CVD risk reduction, by engaging stakeholders at every stage of the research process. As discussed in section 3.1, we engaged patients, peer coaches, office staff, and practitioners in designing the proposal, and stakeholder will continue to be engaged throughout the project by using focus and discussion groups, CABs, and semi-structured interviews, following a collaborative, stakeholder engaged intervention development and dissemination approach. This study represents the culmination of 6 years of community-engaged research, with each subsequent study responding to community expressed needs. The study team's first project in the AL Black Belt responded to community requests for diabetes programs;[14,15] the second project responded to peer coach requests for programs to help individuals with diabetes and chronic pain to be able to exercise;[16] and the third program responded to community member misconceptions about medications used for CVD risk reduction, preventing informed medication taking decisions.[17] This proposed study also responds to a growing interest in rural primary care practices for help with practice transformation and the meaningful use of their electronic health records (EHRs).

6. The conceptual framework guiding this study is highly innovative, proposing that different types of interventions may operate better for clinical situations with high vs. low uncertainty. We will test the concept that structural or procedural interventions, like practice facilitation, may be best suited to improve low uncertainty situations (e.g., algorithms for BP management), and that relationship-based interventions like peer coaching may be best suited to improve high uncertainty situations (e.g., medication or lifestyle adherence). As such, this study will encourage a paradigm shift for selecting types of interventions based on the level of clinical uncertainty.

7. In response to our prior work with practitioners in the Black Belt, we use functional interventions ideally suited to stepping up care, since both practices and patients in rural areas have varied needs. Each of the interventions will operationalize key functions, encouraging customization at both the practice and individual levels, thereby maximizing the potential for widespread adoption. While not an 'adaptive trial design', which requires a prospectively planned opportunity for modifying the design of the study based on interim data analysis, the functional nature of the interventions permits stepping up where most appropriate. For example, in a practice that already knows how to construct registries, the approach to creating a HTN registry may require less intensive effort than in a practice still using paper records.

8. We have designed the study as an implementation trial using the Reach Effectiveness Adoption Implementation Maintenance (RE-AIM) implementation framework and collecting substantial process data designed to enhance eventual scale-up in a wide variety of practice settings commonly encountered in the Black Belt and other rural areas.

9. The Affordable Care Act provides a sustainability framework, strengthened by the January 2015 Centers for Medicare and Medicaid Services (CMS) announcement for its plans to move rapidly to 85% value-based reimbursements within 2 years. This transition will greatly enhance efforts to introduce alternative models of care that are not volume-based, which has been challenging many Black Belt communities, which have been dominated by fee-for-service medicine until now.

PCORI Methodology Standard 1:RQ-5 standard provides guidance on selecting appropriate interventions and comparators for researchers. The standard states: **"**When evaluating an intervention, the comparator

treatment(s) must be chosen to enable accurate evaluation of effectiveness or safety compared to other viable options for similar patients. Researchers should make explicit what the comparators are and how they were selected, focusing on clearly describing how the chosen comparator(s) define the causal question, reduce the potential for biases, and allow direct comparisons. Generally, non-use (or no specific treatment) comparator groups should be avoided unless no specific treatment is a likely option in standard care."[1] Thus, we have elected to utilize practice facilitation and peer coaching as the two interventions to be tested in comparison with enhanced usual care. These interventions are discussed next.

1.2.a Practice Facilitation

Practice facilitation is an increasingly widely used strategy to help practices transition from episode-based, reactive care to prevention of health outcomes and population health management, an approach that has been proposed as the model for primary care service delivery in the 21st century.[18-21] Key features of practice transformation include shifting the focus from single patients to populations, and from physician-centric to team-based clinical management; it emphasizes self-management support and maximizes the use of EHRs, including the creation of registries, audit and feedback programs, and outreach.[22] The National Committee on Quality Assurance (NCQA) offers formal recognition for practice transformation that now includes nearly 7000 practices.[18] The Centers for Medicare and Medicaid Services (CMS) announced on January 26, 2015 that they aim to have 85% of all Medicare fee-for-service payments tied to quality or value by the end of 2016, with 30% of these payments through alternative payment models, such as "advanced" patient centered medical homes.[23] In short, practice transformation has wide support nationally.

Practice facilitation is a highly engaged, flexible consultative service designed to facilitate practice transformation. Practice facilitation certification programs have sprung up, providing a cadre of facilitators to assist primary care practices make the required structural and process changes. Practice facilitation involves a trained, certified individual visiting the practice regularly over several months to teach how to work in teams and fully utilize the potential of EHRs.

Table 2. Facilitator functions and example activities within each function

Functions	Specific example activity
Facilitate delivery system redesign/team management	*Group visits, nurse manages BP using algorithm, "huddles" (quick team meetings for communication)*
Integrate self-management support	*Logbook to track BP, HBPM, education binders in exam rooms, education videos, linkages to community nutrition counseling*
Improve access	*Telephone management*
Facilitate outreach	*Use EHR to create BP registry and population level reports; reach out to patients with missed appointments*

BP = blood pressure. EHR = electronic health record. HBPM = home BP monitoring.

According to Agency for Healthcare Research and Quality's (AHRQ's) *Practice Facilitation Handbook,* facilitators "support change in practices by focusing… attention on the process of change and by empowering members of the practice to engage in the change process *together."*[24] Dr. Lyndee Knox, lead author of the AHRQ *Handbook*, was a consultant on this project during the pre-award and UH2 planning phases. Facilitators establish trust with the practice team; help them to assess the practice, set goals, and build capacity to gather data to create performance metrics; and help the practice monitor its performance, map workflows, and implement plan-do-study-act cycles to improve the quality of care (see Table 2). Assumptions behind practice facilitation are that many practices are inadequately resourced and lack experience and skills to make transformative changes on their own. A key aspect of practice facilitation is the relationships facilitators develop with the practice staff, similar to the Cooperative Extension model, in which agents develop relationships with farming families to facilitate the implementation of evidence-based farming practices.[25] By definition, practice facilitation interventions are highly tailored, stepping up activities if the practice is not accomplishing its goals as tracked through regular performance reports drawn from its own data.

1.2.b The Effectiveness of Practice Facilitation and Practice Transformation

While the enthusiasm for practice transformation is high, early evaluations of pilot studies are mixed. For example, a 2013 systematic review of 19 studies of practice transformation interventions demonstrated that practice facilitation increased NCQA recognition, but achieving recognition was associated with only a small

positive effect on patient experiences, preventive services delivery, and staff experiences, with reduced Emergency Department (ED) utilization but no effect on overall costs or clinical outcomes.[26] A 2014 report on the Southeastern Pennsylvania Chronic Care Initiative, an early and large multi-payer patient centered medical home (PCMH) pilot, showed that while all of the participating 32 practices achieved NCQA PCMH recognition status and adopted new structural capabilities such as registries, only one of 11 quality measures improved significantly with no significant changes in utilization or costs of care.[27] A 2013 report on a Rhode Island pilot demonstrated substantial increases in NCQA scores but no changes in quality metrics.[28] A randomized controlled trial of 32 practices in NY that included 18 months of practice facilitation, revised payments, and embedded care management support improved BP control by 23 percentage points in intervention practices, but the final control BP rate was only 36%, and there were very modest additional improvements in only one other of the 11 quality metrics.[29] An evaluation of a 9-year practice transformation in 17 FQHCs in lower NY State revealed that mean A1c declined from nearly 11% to below 9% among those diabetes patients with A1c >9% as the program was implemented, but mean A1c for those with A1c <9% actually rose slightly, and overall glycemic control remained at 8%, well above the goal of 7% recommended by the American Diabetes Association (ADA).[30]

A report by Leykum, et al, may help to understand what may be underlying these findings of relatively modest effectiveness and possible floor effects.[31] Their qualitative study was designed to generate new theories to explain why some of their group's collective studies worked and others did not. They proposed that uncertainty may play a central role in the effectiveness of interventions. They concluded that structure or process based efforts, like practice facilitation with its quality improvement (QI) focus, may be most effective for situations where required clinical decision-making is relatively simple with low uncertainty (e.g., algorithms for BP control, HTN patient who has not been seen for a year should be recalled). On the other hand, relationship based interventions like peer coaching may be more effective for more complex clinical management situations with high uncertainty (e.g., medication adherence, healthy eating). Most practice facilitation evaluations to date have focused more on quality, costs and objectively assessed experience of care, and less on the quality of the relationships that are established in the PCMH. In fact, high quality, trusting relationships with healthcare providers/teams may be especially challenging for minorities living in low socioeconomic circumstances, especially in rural areas.[32]

1.2.c Peer Coaches

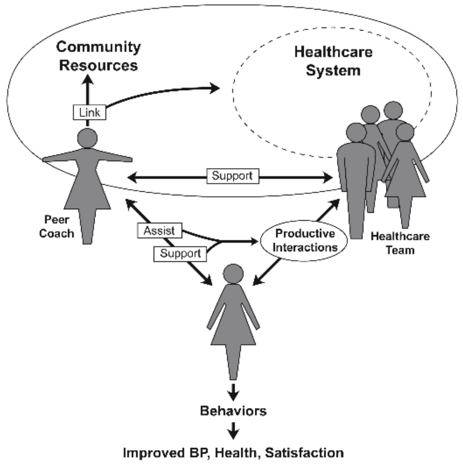

Figure 3. Peer coaches in the context of the Chronic Care model.

Peer coaches, also called peer advisors, community health workers, community health advisors, or *promotoras*, are people who live in the same community as the targeted population. They have an in-depth understanding of the day-to-day challenges of living with a chronic disease like HTN. They receive training in motivational interviewing, which they use to coach other community members with HTN on how to improve self-management within the context of their own lives, helping them to overcome challenges to achieving a healthy lifestyle and taking medications (Figure 3). First and foremost, they provide social and emotional support and encouragement, but they also assist patients by motivating them to understand which behaviors are increasing risks and to engage in risk management. They link their clients into community resources and back to the practice, encouraging the development of continuous relationships with a usual source of care. The key functions of peer coaching interventions are therefore assisting, supporting and linking into resources and care. By nature, peer coaching interventions are multi-level, since they reach into the community as well as the health system, and multi-component, stepping up as warranted by the individual's needs.

> ***"I can talk to her about anything and she always be nice. I just love working with her, it makes me want to do right. She really understands how hard it can be."***
>
> *--Comment from a participant about his peer coach.*

Interacting with a live peer coach is a potent experience because participants have discussions with someone like themselves, building a supportive relationship while the peer coach assists them in carrying out the healthcare team's recommendations and in understanding educational materials. At the core of peer coaching interventions are the relationships patients forge with the peer coach. In our past programs, 96% of participants were either extremely or highly satisfied with working with their peer coach. Many of our peer coaches continue to speak regularly with their former clients, even years after the formal program has ended. A participant in one of our studies summarized his relationship with his peer coach this way: "*She really cared about me. When we first started talking I didn't really know what to think, but she really helped me. I started growing a garden because of her, and now it's so plentiful I want to share the vegetables with her."*

In preparation for the proposal that led to the current study, we engaged 40 Black Belt residents with chronic diseases in 5 focus groups to understand their perspectives on the challenges they face in curbing CVD risk. While participants had many questions about their health, many did not feel comfortable asking their doctor or nurse. It is important to note that Tuskegee, site of the infamous syphilis experiment, is located in the AL Black Belt, and this experience has left a deep mark in the African American community, creating additional barriers to trust.[33] One participant said, "*I just want to get out of there as quickly as possible. You ask too many questions they start looking, and then they start finding things."* Misunderstandings are common; e.g., a participant noted, "*They always experimenting on you with those high blood pills, changing up the pills every time I go."* Consistently, participants expressed greater comfort with their peer coach than with members of their healthcare team.

1.2.d Evidence of Effectiveness of Peer Coaching for HTN

The evidence of the effectiveness of peer coaches to improve BP control spans over 3 decades. A 2007 review of 14 studies included 8 randomized controlled trials (RCTs), many engaging urban African Americans; their analysis favored intervention effects ranging from 0-8 mm Hg systolic blood pressure (SBP), and improved control ranging from 0-15%.[34] The Centers for Disease Control and Prevention (CDC) is a strong supporter of community health workers and has promulgated training materials and policy briefs on how to implement such programs.[35] However, few studies of peer coaching have been conducted in rural settings, and even fewer in the Black Belt region. Many interventions included face-to-face meetings or group meetings, which is not feasible in the Black Belt due to the distances and cost of transportation. It is unknown whether an entirely telephone-delivered peer coaching intervention can achieve significant reductions in BP in the Black Belt region, a knowledge gap this study will fill.

2. Study Objectives

The central objective of the "**Collaboration to Improve Blood Pressure in the US Black Belt – Addressing the Triple Threat**" is to rigorously compare two strategies designed to improve BP control in primary care practices serving rural Southeastern African Americans with low socioeconomic status (SES).

The AHRQ Disparities Report identifies individuals with rural residence, minority ethnicity, and low SES as being at high risk for poor health outcomes. The "Black Belt" region stretches from eastern Texas in an arc to Maryland and includes residents with all 3 of these characteristics – the proverbial triple threat. This traditionally agricultural region is characterized by steep poverty, low educational attainment, scarce resources, and mostly African American residents. The Black Belt is in the heart of the Stroke Belt, a geographic area long recognized to have the highest CVD mortality in the US. The AHRQ Disparities Report also cites that the Southeast has lower quality of care than the rest of the US, thus effective strategies to optimize CVD prevention in general and HTN control specifically are urgently needed here.

We draw on the growing evidence that **practice facilitation** can speed the transition to high quality, evidence-based care. Practice facilitation is a highly customized, staged approach to helping a practice to implement *process and structural changes* to enhance the quality of care and improve patient and staff satisfaction, e.g., by implementing protocols for algorithm-driven BP medication management, and creating registries and audit and feedback systems using their EHRs. Evidence that practice facilitation can help practices to change their structure is strong, but evidence on outcomes such as achieving BP control is limited. An alternate approach to improving CVD risk factors that is more *relationship-focused* and with growing evidence of effectiveness involves the use of **peer coaches**. We and others have shown that peer coaches can be particularly effective in Black Belt communities, where mistrust of the healthcare system is common. Further, CVD risk reduction through BP control requires challenging behavior modifications that can be difficult to achieve using office-based interventions. Our study responds directly to the need for Black Belt communities to know how much benefit their communities can expect to derive from structural and process interventions like practice facilitation, or from relationship-focused interventions like peer coaching, or from both. Using well-established community-based partnerships and the RE-AIM implementation framework, our Specific Aims are:

2.1 Year 1

Aim 1: Engage rural primary care practices, HTN patients, peer coaches, and Community Advisory Boards in AL and NC to collaboratively finalize a practice facilitation intervention, a peer coaching intervention, and a hybrid intervention integrating peer coaching and practice facilitation, all designed to improve BP in African Americans.

Aim 2: Create the data systems for the trial.

2.2 Years 2-5

Aim 3: Enroll 80 practices and 25 African American patients with uncontrolled HTN at each practice (total n=2000) in a cluster-randomized, controlled, pragmatic implementation trial to evaluate the three multi-component, multi-level functional interventions finalized in the UH2 phase compared with enhanced usual care. **Our primary hypotheses are that each of the three interventions will improve BP more than enhanced usual care. Our secondary hypothesis is that the hybrid intervention will result in greater improvements in BP control than the other interventions.** While we aim to control BP in >75% of participating intervention patients overall, the trial is designed to detect ≥15% difference in BP control (defined as <140/90 mm Hg, the primary outcome) between all three interventions and the enhanced usual care arm.

Secondary outcomes will include group mean BP differences between baseline and follow-up; quality of life; patient satisfaction; healthcare utilization; and provider and staff satisfaction. The study is designed to examine differences by sex, age, depression, health literacy, and numeracy.

Aim 4. Establish scalability of the intervention throughout the entire Black Belt region using extensive process data intended to facilitate future implementation across a wide variety of practices. To accomplish this goal, we will utilize practice characteristics, patient characteristics, intervention implementation variables and fidelity measures, as well as focus groups and interviews with key stakeholders, including patients, peer coaches, facilitators, practice staff, and clinicians.

2.3 Hypotheses

The main hypothesis that will be tested in this study is that there will be statistically significant differences among the study groups. Specifically:

1) The practice-levels rates of achieving hypertension control at one year will be higher in the **practice facilitation** group compared to the enhanced usual care group.

2) The practice-levels rates of achieving hypertension control at one year will be higher in the **peer coaching** group compared to the enhanced usual care group.

3) The practice-levels rates of achieving hypertension control at one year will be higher in the **integrated practice facilitation with peer coaching** group compared to the enhanced usual care group.

We will test several secondary hypotheses, including a test of whether the combined interventions are superior to either intervention individually, and a series of analyses examining changes at the individual patient level. Prespecified subgroup analyses will be conducted at the patient level, including examination of differences by sex, age (above and below age 60), literacy/numeracy, and depressive symptoms. See section 8 for details of the analytic plans.

3. Study Design

3.1 Research Design and Methodology: Stakeholder Engagement

PCORI Methodology Standard 2:PC-1 is: "Engage people representing the population of interest and other relevant stakeholders in ways that are appropriate and necessary in a given research context. People representing the population of interest include individuals who have the condition or who are at risk of the condition and, as relevant, their surrogates or caregivers. Other relevant stakeholders may include clinicians, administrators, policy makers, or others involved in healthcare decision-making. Stakeholders can be engaged in the processes of:

- Formulating research questions;
- Defining essential characteristics of study participants, comparators, and outcomes;
- Identifying and selecting outcomes that the population of interest notices and cares about (e.g., survival, function, symptoms, health-related quality of life) and that inform decision making relevant to the research topic;
- Monitoring study conduct and progress; and
- Designing/suggesting plans for dissemination and implementation activities.

When applicable, research proposals should describe how these stakeholders will be identified, recruited, and retained. If engagement is not necessary or appropriate in these processes, explain why."[1] This study is designed to align with this Methodology Standard, using a highly stakeholder engaged approach.

3.1.a Stakeholder Engagement for Input into the Study Design and Procedures

This community-partnered research project engages stakeholders at every phase of the research. We started by engaging stakeholders during the planning phase to select the study question and we continue to engage them throughout the course of the study. Our general approach involves 1) embedding members of the stakeholder group on the research team; 2) conducting qualitative research prior to the start of the project planning; 3) conducting additional qualitative research (focus groups, nominal groups, and interviews) during the study; and 4) regularly reaching out to CABs for advice.

3.1.b Input from Hypertensive African American Residents of the Black Belt

In our past studies, we have embedded members of the targeted patient population into the research team, which has resulted in invaluable advice and modifications of our preliminary plans. We will again include on the research team a research assistant (T. Davis) who grew up in Boligee, AL, a Black Belt town with fewer than 400 residents. Ms. Davis provides ongoing input at research team meetings. In addition to integrating members of our target audience in the research team, we conduct qualitative research to obtain input from a broader range of stakeholders.[17,36,37] As part of the planning process for the proposal, we conducted 5 focus groups with 40 Black Belt residents with HTN or diabetes requiring ongoing medication management in 2013 to understand the barriers they face in attempting to control cardiovascular risk factors including HTN. Top barriers included inability to keep doctor appointments, lack of exercise, limited income, lack of will power, and pain as a barrier to physical activity. These findings have led us to target telephone support as a component of the practice facilitation intervention, and motivational interviewing in the peer coaching intervention.

During the year-long planning phase of the study, we engaged in ongoing discussions with area residents with HTN in both AL and NC to further inform the design of the study as well as the interventions. These discussions included community member perspectives on the challenges they face accessing medical care and advice, barriers to medication adherence, community-level barriers to healthier diet and physical activity, attitudes toward working with a peer coach potentially from their same community (including potential concerns), attitudes toward home BP monitoring, as well as specific input on study design questions (e.g., preferences for recruitment and informed consenting, preferences for retention activities, input on selection of outcome measures). We recruited patients of area practices and individuals with HTN in the social networks of

our current cadre of over 60 trained peer coaches. We conduct in-person groups in community settings, either at the practice or in a community location. A trained moderator led the discussions, in many cases community coordinators based at the partnering community non-profit organizations (Health and Wellness Education Center of Livingston, AL, and the West Central Alabama Community Health Improvement League).

3.1.c Input from Peer Coaches

Our two community coordinators Ms. Clark and Ms. E. Johnson are both Black Belt residents and experienced peer coaches. They provide ongoing input on the peer coaching perspective, as well as supervising the AL peer coaches during the study.

In preparing the proposal, we conducted 4 focus groups with 24 peer coaches and office staff to learn their perspectives on barriers to curbing CVD risk in the Black Belt. Lack of education was cited as the major barrier, followed by transportation and limited economic means. These results led us to incorporate the Patient Activated Learning System (PALS) as enhanced usual care and as a resource for the peer coaches in the intervention (see section 6.3.a). We will continue to engage peer coaches throughout the study period through biweekly conference calls to help with intervention development; participant, peer coach, and practice recruitment plans; and data collection plans, among other topics. These meetings occur weekly by teleconference and monthly when the study team travels to the Black Belt.

3.1.d Input from Primary Care Providers Practicing in the Black Belt

Our team includes practicing physician investigators, and Dr. Harrington leads the AL Practice Based Research Network (PBRN), assuring the physician perspective is represented on the research team. In addition, in preparation for this proposal, we engaged 12 primary care providers in 2 focus groups to learn their perspectives on barriers to curbing CVD risks in the Black Belt. Providers cited motivation, noncompliance and economic hardship as the top barriers. These perspectives led us to emphasize outreach, self-management support, and telephone support in the practice facilitation intervention, and motivational interviewing in the peer coaching intervention. We will continue to engage physicians and office staff in the course of the study using teleconferencing and face-to-face meetings.

3.1.e Input from Experienced Practice Facilitators

Drs. Viera, Halladay, and Cummings have conducted several studies of practice redesign, and their team includes experienced facilitators. We engaged them in the development and finalization of the practice facilitation intervention protocol, obtaining their input during intervention design through discussion groups. The experienced NC practice facilitators also serve as resources for the AL practice facilitators as they customize the practice facilitation intervention to individual practices during the intervention implementation phase. Lyndee Knox, PhD, author of the AHRQ *Practice Facilitation Handbook*, consulted for the study during the planning phases.

3.1.f Community Advisory Boards

The CABs were designed to obtain high-level input from stakeholders who will be critical for scaling the interventions throughout the Black Belt regions, should they be found to be effective. Each participating state has their own CAB, and the composition of these boards is shown in Table 3. The CABs meet annually and serve as a between-meeting resource.

The first CAB meetings focused on challenges to implementing the practice facilitation and peer coaching interventions, and advice on how to design the interventions to enhance sustainability and widespread adoption. The format of this discussion was a nominal group, a structured group process designed to develop a prioritized list of responses to a question. Participants first generate a list of unique responses, then prioritize the most important among this list by voting for their top three choices. The total number of available votes is then used to generate the percent of available votes to facilitate comparison across groups.

Table 3. Composition of the Community Advisory Boards for the study.

State	Member	Perspective
ALABAMA	Kierra Powell, Grants Coordinator, Constituent Service Representative, U.S. Representative Terri Sewell (AL-7)	Elected representatives
	Dow Briggs, MD, Chief Business Officer, Blue Cross Blue Shield	Private health insurance company
	Terry Knight, Senior Vice-President, Viva Health, Inc	HMO, Accountable Care Organizations
	Maggie Jolly, Assistant Pastor of the Greater St. Paul CME Church and community health worker	Faith-based organizations
	Sondra Reese, MD, Director, Bureau Health Promotion & Chronic Disease Bureau, Alabama Department of Public Health	Public Health, safety nets
NORTH CAROLINA	Angela Bryant, NC Senator, District 4	Elected representatives
	Sam Cykert, MD, UNC Director, Program on Health and Clinical Informatics, PI, Fast Pace NC	Health information technology
	Annette Dubard, MD, Director, Informatics, Quality, and Evaluation, Community Care of NC	Population health management for Medicaid
	Jan Hutchins, Director, Medical Services UNC Physicians Network	Physician networks
	Ben Money, MPH, CEO, NC Community Health Center Association	Public Health, safety nets
	Al Richmond, Executive Director, Community-Campus Partnerships for Health	Community-academic partnerships to promote health

Table 4. Input of the CAB in the first year of the study.

Practice Facilitation	Percent available votes	Peer Coaching	Percent available votes
ALABAMA			
Challenges to implementation			
Clinics under resourced	19%	Trust	15%
Practices are busy	15%	Coach reliability	15%
Convincing practices of value	11%	Acceptance of coach by professional	11%
Clinics under staffed	9%	Recruiting coaches long term	9%
Conservative/not open to new	9%	Defining value for patients (why would I want a coach)	9%
		Tailoring coach to patient, chemistry	9%
How to design for sustainability			
Reimbursement model	19%	Robust reimbursement	39%
Highly tailored	19%	Ensure provider buy-in	11%
Burden neutral/doesn’t hurt	17%	Cost effectiveness	9%
Demonstrate long-term outcomes	9%	Stopping limits – when is enough	9%
Build in feedback on QI metrics	9%	Metrics for pts, peers, providers	9%
NORTH CAROLINA			
Challenges to implementation			
Practices overwhelmed	44%	Training, competencies	22%
Impact on practice, patient flow	20%	Being aware of coaching activities already in place	13%
Competing interests/similar projects	9%	Getting the practice to accept coaches	13%
Financial pressures not aligned	9%	Identifying coaches	11%
How to design for sustainability			
Clear messaging on impact on patient outcomes	24%	Implementation details and costs of the program	33%
Work to align incentives	22%	Assure coaches are part of clinical care team	24%
Training for practice staff	18%	Build on existing efforts already in the community	13%
		Engage stakeholders to advocate for program	13%

The results of the first CAB meetings are shown in Table 4, limited to only those items that received at least 9% of available votes. The CABs had some advice that was similar across the two states, for example, both CABs emphasized that the under resourced and overwhelmed state of many practices will be an implementation barrier for practice facilitation, and that data on the benefit to patients of this type of program will be important for long-term sustainability. On the other hand, the AL CAB members voiced concerns about the conservative nature of doctors in the rural Black Belt, whereas the NC CAB members advised that numerous projects ongoing in the state may pose a challenge.

The advice from the CABs was used to shape the approach to recruiting practices, with a set of talking points developed for recruitment discussions. The advice also shaped the type of data collected, emphasizing process and cost data to facilitate implementation.

3.1.g Community meetings

In the final year of the study, we will plan a series of community meetings to share the findings of the study and plan for sustainability should the results prove favorable. We will work with our community partners to identify suitable venues for these meetings and feature community members prominently in the presentations.

3.2 Design of the Study

This study is a cluster randomized trial with pre-planned subgroup analyses (Figure 4). We will compare the effectiveness of 3 distinct interventions to achieve BP control in comparison with enhanced usual care in our targeted high-risk communities. This design in essence allows us to carry out three trials simultaneously, sharing a single control group. In addition to testing each intervention versus the control group, this design will also allow us to test the interventions against one another. Since our conceptual framework posits that a structure/process intervention will operate differently than a relationship-based intervention, this design is optimal for testing the effectiveness of each type of intervention on BP control.

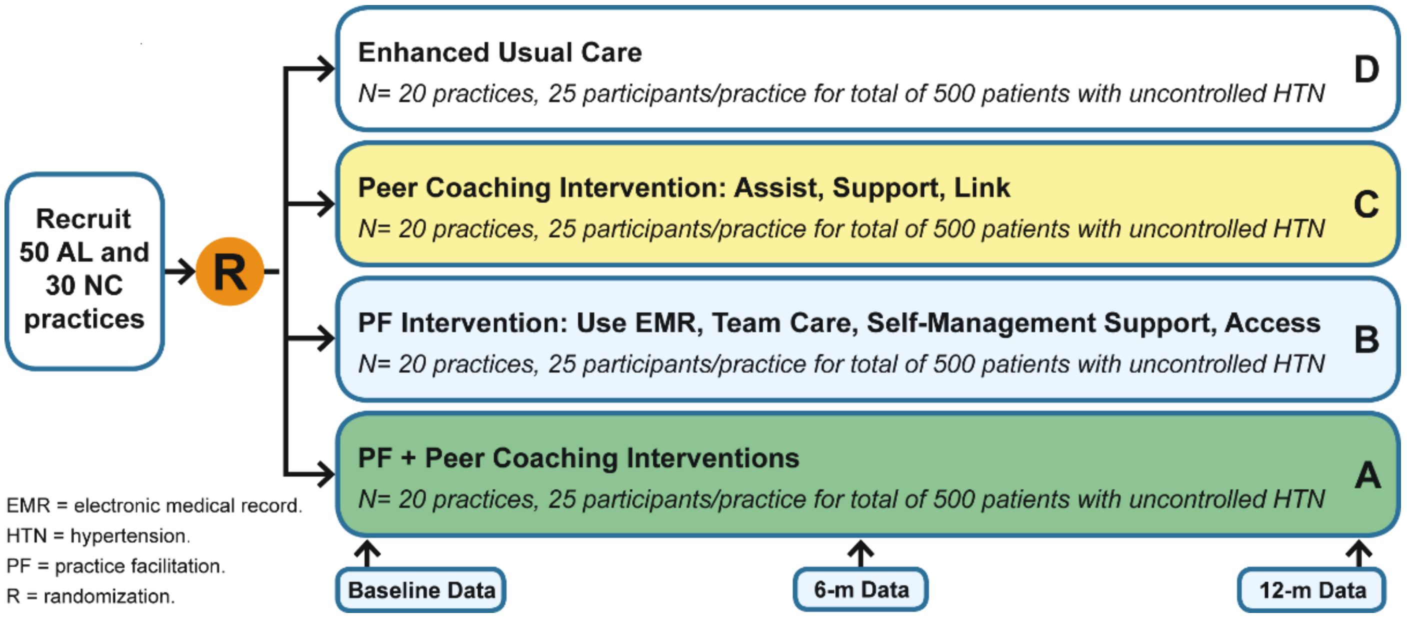

Figure 4. Design of the Cluster-Randomized, Controlled, Pragmatic Trial. *All practices receive enhanced usual care (Patient Activated Learning System [PALS], home BP monitors, practice tips, see 6.3.a).*

3.3 Study Population: Inclusion / Exclusion Criteria

The inclusion and exclusion criteria for trial participants are shown in Table 5. Concordant with the pragmatic trial context, inclusion and especially exclusion criteria are kept to a minimum to maximize generalizability.

Table 5. Patient inclusion and exclusion criteria for the trial.

Inclusion criteria	Exclusion criteria
• *African American adults aged 19-85 years*<br>• *Uncontrolled hypertension**<br>• *Black Belt resident*<br>• *English speaking*<br>• *Willing to work with a peer coach*<br>• *Willing to sign informed consent*<br>• *Has access to a telephone*	• *Plans to move out of the area within the next two years*<br>• *Advanced illness with limited life expectancy*<br>• *Pregnant or plans to get pregnant in the next year*<br>• *Advanced chronic kidney disease (estimated glomerular filtration rate <45 ml/min/1.73 m^2)*<br>• *Unwillingness to work with a peer coach or to sign informed consent*<br>• *Lack of phone access*

*Uncontrolled hypertension will be determined by a mean systolic blood pressure ≥140 mm Hg calculated using at least 1 BP value documented in the medical record in the preceding year AND a value ≥140/90 mm Hg assessed by the research assistant at the time of screening.

3.4 Definition of Hypertension

Hypertension (HTN), or high blood pressure, is a common disorder affecting about 70 million Americans according to the CDC.[38] As described in the Background and Significance, it disproportionately affects African Americans, and it increases risks for a host of health outcomes including stroke, heart attack, kidney failure requiring dialysis, vascular disease, and heart failure, among others.

Hypertension has long been defined as blood pressure that is 140/90 mm Hg or greater. Table 6 shows the categories of abnormal BP according to pre-2017 guideline, including pre-hypertension and hypertension. Hypertension is commonly categorized into Stage 1 or Stage 2, depending on the degree of elevation.

Table 6. Stages of High BP in Adults.

Stages of High Blood Pressure in Adults	Systolic		Diastolic
Prehypertension	120-139	OR	80-89
High Blood Pressure – Stage 1	140-159	OR	90-99
High Blood Pressure – Stage 2	160 or higher	OR	100 or higher

According the seventh Joint National Committee on the Prevention, Detection, Evaluation, and Treatment of High Blood Pressure, individuals with a reading in the Stage 1 range should have a second measurement within 2 months to confirm the BP elevation. Those with Stage 2 hypertension need not wait for confirmation prior to initiation of evaluation and treatment.

As a practical matter, BP even within a single patient is highly variable from visit to visit. The Counseling African Americans to Control Hypertension (CAATCH) community based intervention to control hypertension enrolled patients with BP ≥140/90 mm Hg at baseline; the investigators found that >40% of both intervention and control subjects were controlled at follow-up[39,40]. This suggests that unless more stringent criteria are used to identify uncontrolled hypertension, the results of this study could also be impacted by a similar regression to the mean. However, although at least two trials in pragmatic settings have used higher thresholds of BP higher than 140/90 mm Hg as inclusion criteria, none had an empiric justification for the higher levels used. It is worth noting that in one of these trials, the use of the higher threshold was prompted by community practitioner requests to focus on higher risk patients.

Absent an established approach to minimizing regression to the mean in hypertension trials, we examined data from the Antihypertensive and Lipid-Lowering Treatment to Reduce Heart Attack Trial (ALLHAT), which was a large, pragmatic, hypertension trial in which 50% of participants achieved BP control at 1 year.[41] We therefore used all BP measurements through the first 9 months of the trial to predict whether hypertension would be controlled at one year. Overall we found that approximately 39% of those uncontrolled at 9 months were controlled at 12 months, a similar regression to the mean as in the CAATCH trial. Using classification trees we found that the most important predictor was a mean blood pressure through 9 months of >148 mm Hg. This group comprised approximately 60% of those uncontrolled at 9 months but had a lower control rate at one year of 30%. To simplify logistics, in this study, uncontrolled hypertension was to be defined as a mean systolic blood pressure ≥150 mm Hg in the year prior to enrollment including at least one blood pressure reading over the preceding 12 months. Participants would also be required to have a measurement by the research assistant on the day of enrollment of 140/90 mm Hg (140 systolic or 90 diastolic) or greater.

In November, 2017, the 2017 ACC/AHA/AAPA/ABC/ACPM/AGS/APhA/ASH/ASPC/NMA/PCNA Guideline for the Prevention, Detection, Evaluation, and Management of High *Blood Pressure* in Adults was announced. This guideline proposed that hypertension should be defined as a blood pressure of ≥130/80 mm Hg, and that blood pressure between 130/80 and 139/89 mm Hg should be defined as Stage 1, with values of 140/90 mm Hg and above defined as Stage 2 hypertension. This major shift in the definition of hypertension prompted the study group to reconsider our inclusion criteria. Our recruitment experience showed that many patients were referred for consideration for enrollment in the study, having been informed by their physician that their blood pressure was uncontrolled. The requirement to have a mean systolic blood pressure of 150 mm Hg or higher over the previous year as well as a blood pressure of 140/90 or greater on enrollment day made many of these patients ineligible for the trial. This created confusion in our partnering clinics. In addition, recruitment was not completed within the allotted 90 day period at some clinics. These considerations coupled with the new definition of hypertension led us to modify the inclusion criteria to require a mean in-clinic systolic blood pressure of 140 mm Hg over the previous year (i.e., the 150 mm Hg criterion was lowered to 140 mm Hg). This modification resulted in the trial focusing on treatment of Stage 2 hypertension according to the 2017 guidelines.

3.5 Study Outcomes

3.5.a Primary Outcome Defined

The RFA for this study required that the primary outcome be the improvement in BP control to less than Stage 2 hypertension between baseline and follow-up between practices in any of the intervention arms (A, B, or C in Figures 4 and 5) and the enhanced usual care arm (D) (Table 7). We operationalize this as a patient-level outcome of the proportion of patients with controlled Stage 2 hypertension in the relevant trial arm at the one-year follow-up.

3.5.b Secondary Outcomes Defined

Secondary outcomes include the change in systolic BP between baseline and follow-up; quality of life (depressive symptoms, physical and mental functioning); and patient and practice team satisfaction.

Preliminarily, we propose a target of controlled Stage 2 hypertension to mean <140/90 mm Hg for all participants because of the very high risk African American sample and because a single BP goal is easier to implement in real-world settings. However, several evolving developments may influence this goal. First, there was considerable controversy following the 2014 Evidence-Based Guideline for the Management of High Blood Pressure in Adults (JNC8) recommendation to relax the BP target for patients with hypertension aged ≥60 years. Second, the Systolic Blood Pressure Intervention Trial (SPRINT) was stopped earlier than its projected project period due to unexpectedly strong beneficial effects of BP control to a target of 120/80 mm Hg.[42,43]

In accordance with **PCORI Methodology Standard 1:RQ-6**, we will "provide information that supports the selection of outcomes as meeting the criteria of "patient-centered" and "relevant to decision makers,"[1] such as patient and decision-maker input from meetings, surveys, or published studies." Our discussions with patients, providers, peer coaches, practice facilitators, and the stakeholders engaged in the CABs shaped the selection of the study outcomes, which are both patient-centered and relevant to decision makers. As described in the Background and Significance (section 1), high blood pressure is a leading killer among African Americans, and leads to significant morbidity. Control of high blood pressure is a high priority in our partnering communities, confirming that this outcome is patient-centered.

Table 7. Primary and secondary outcomes

Type of measure	Measure
Primary outcome	*Difference in BP control between trial arms*
Secondary outcomes	*Difference in change in BP between baseline and f/u*<br>*Depressive symptoms*<br>*Physical and mental functioning*<br>*Patient, provider, staff satisfaction*

3.6 Pragmatic Elements

This study is designed as a pragmatic implementation trial. The elements which meet the Pragmatic-Explanatory Continuum Indicator Summary (PRECIS) criteria for pragmatic trials are shown in Table 8.

Table 8. Our study's features that meet PRECIS Criteria for pragmatic trials.

PRECIS Domain/ Pragmatic Principle	Design Approach and Estimated Levels of Clinical Research Pragmatism (1 = Extremely; 2 = Moderately; 3 = Somewhat Pragmatic)
Participant eligibility criteria	*Minimal exclusion/ inclusion criteria: adult African American adults with uncontrolled HTN (1)*
Participant adherence	*Patient adherence captured via self-report, medical record review, cell phone data (1)*
Practitioner expertise	*PF intervention draws on each practice's self-knowledge; peer coaching draws on coach training and experience (1)*
Practitioner adherence	*Practice adherence captured via self-assessments (1); peer coaching adherence captured via cell phone use and manuals (1)*
Flexibility and complexity of the intervention	*Multi-level, multi-component functional interventions, both maximally flexible and tailored to local/individual needs and circumstances (1). Flexibility adds a degree of complexity (2)*
Allocation concealment and blinding	*Data collectors not blinded to allocation (1). Practices not blinded due to nature of interv'n (1). Pts blinded to EUC vs. PF allocation (1), not blinded to peer coaching allocation (1).*
Sample size based on hypothesized effect size	*Large number of practices with 25 patients/practice maximizes power for main analysis at practice level, while allowing for subgroup analyses at the patient level; incorporate conservative attrition estimates (1)*
Randomization scheme	*Block randomization stratified by practice type (FQHC or not) (2); cluster randomization (1)*
Clinically meaningful pt.-centered outcomes	*BP and BP control directly and causally linked with CVD outcomes; quality of life and satisfaction highly patient-centered (1)*
Follow-up intensity	*3 data collection visits at practice over 1 year, follow-up phone calls to practice 1 year later for large subsample (2)*
Analytic approaches	*Primary analysis intent to treat, adequately powered subgroup analyses for especially high-risk groups (men, age <60 years, low literacy, depressive symptoms) (1)*

BP = Blood Pressure, CVD = Cardiovascular Disease, EUC = Enhanced Usual Care, FQHC = Federally Qualified Health Centers, HTN = Hypertension, PF = Practice Facilitation.

3.7 Randomization

3.7.a Cluster Randomization

We use a cluster randomized design for two reasons. First, the practice facilitation intervention acts at the practice level, requiring practices to be the unit of randomization. Second, patients are nested within practices, thus the design should reflect this non-random distribution of the study population for analyses at the patient level. We also respond to a recent call for designs that engage more practices with fewer patients per practice in studies of practice change interventions.[18,22] As discussed in section 8, the inclusion of 80 practices provides sufficient power to detect clinically important differences in BP control to <140/90 mm Hg between the intervention arms and the enhanced usual care arm. While the RCT design is the most robust experimental design available, it is often not feasible in real-world settings. However, several things make this design

feasible for the study. First is our experience implementing RCTs in practice-based and community-based research in the Black Belt. Second, we offer enhanced usual care as a comparison condition, which is attractive to practices and participants alike, who often wish to avoid being in the “control arm” and “getting nothing.”

3.7.b Practice Randomization

There are some structural differences between practices located in the Black Belt region. For example, FQHCs tend to have well developed EMRs and quality reporting systems and interventions, whereas the private practices tend to have fewer activities in these areas and are more likely to use a traditional model of care delivery. These structural differences may impact baseline measures and response to the interventions. Therefore, at each stage of the implementation, we will actively work to balance allocation across these two practice types: FQHC and non-FQHC. We will also work to achieve balance by state (AL and NC). The study’s biostatistician, Dr. Richman, will use a random number generator to randomize the practices and will be responsible for assuring balance across practice type, state, and study arm using permuted block randomization with sizes of 2 and 4 to minimize imbalance. Randomization will occur for each stage-group when they are randomly selected for implementation. We will not randomize practices at recruitment because attrition between recruitment and readiness could cause imbalance.

The RCT portion of this study has two distinct stages, beginning with the initial vanguard phase described below in section 6 followed by staged implementation (see Figure 5). From among the practices ready for randomization by June 2016, we will select 4 as the ‘vanguard.’ The 4 vanguard practices will be randomized to each of the 4 treatment arms/cells of the trial, such that one practice is in each of the 4 arms/cells.

Randomization assignments for the rest of the trial will be drawn from the remaining pool of recruited practices meeting readiness criteria. This second implementation phase will be rolled out in stages as shown in Figure 6. Because the pool of recruited practices will change due to recruitment, readiness, and attrition, approximately 3-6 months before each stage, that stage’s participating practices will be chosen in groups of 10-12, with Dr. Richman assuring balance across practice types (FQHC vs not) and also across states (AL vs. NC) using a block randomization approach. The goal is to recruit 50 practices in AL and 30 in NC, reflecting the relatively larger geographic region in AL. The allocation will be examined carefully prior to the final two stages and, depending on the proportions of practice types and number of practices in each state, the randomization may be changed slightly to ensure balance of the final cohort. Because recruitment occurs well in advance of randomization, Dr. Richman will be able to ensure balanced allocation without compromising random assignment.

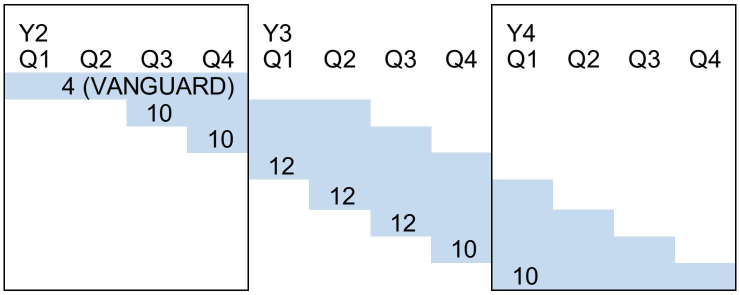

Figure 5. Staged implementation scheme for the 80 participating practices.
Numbers indicate the number of practices per wave. Actual accrual pace may vary.

3.8 Blinding

Ideally, participants, practices and data collectors as well as any study staff interacting with participants should be blinded to the practice’s randomization status in order to minimize biases. However, in many trials of behavior change interventions like this one, this is not possible. Nevertheless, we will implement blinding where feasible. Data collectors will be separated from research team members engaged in intervention implementation. Patients in the enhanced usual care and practice facilitation-only arms will be blinded to the

practice’s randomization status. However, it will not be possible to blind practices to their randomization status, and it will not be possible to blind patients in the peer coaching arms to the practice’s randomization status.

3.9 Expected Duration of Subject Participation

The expected duration of subject participation is one year.

4. Recruitment of Practices, Facilitators, and Coaches

This study will recruit practices (section 4.1), practice facilitators (section 4.2), peer coaches (section 4.3), and participants (section 5). In accordance with **PCORI Methodology Standard 2: PC-2** we will "identify, select, recruit, and retain study participants representative of the spectrum of the population of interest and ensure that all data are collected thoroughly and systematically from all study participants."[1]

4.1 Recruitment of Practices

4.1.a Number of Practices

We will recruit 50 practices in AL and 30 in NC with a higher target in AL because this region demonstrates slightly worse BP control than NC, based on BP control rates reported by FQHCs in each state's Black Belt counties (Table 1). AL also contains a larger portion of the Black Belt by area (see Figures 1 and 2), and AL has more single physician practices, which have been shown to face considerable challenges to improving BP control in their patients with HTN.[18] We will therefore make special efforts to reach out to single physician practices. As part of the proposal phase of this study, we obtained letters of support from over 300 practices (Table 9).

Table 9. Sampling frame for practice recruitment

Recruitment Source	Practices
AL PBRN	30
Dr. Safford's Black Belt programs	130
AL AHEC	150
Deep South CME Network – AL rural	200
AL Primary Care Association	7 FQHC's
Medical Alumni Society of AL	220
**TOTAL AL**	**220**
Vidant of NC	68
Opportunities Center of NC	2
Carolinas Healthcare	5
Goshen Medical Center of NC	25
Robeson Healthcare Centers of NC	7
Kinston Medical Specialists of NC	5
**TOTAL NC**	**95**

AHEC = Area Health Education Center. CME = Continuing Medical Education. PBRN = practice-based research network.

As of June 2016, we have received signed letters of agreement from 29 practices; the practices cannot be named in this protocol until they have received Federal Wide Assurance numbers, and this application process is in progress. These practices were selected for the first wave because the recruitment team, led by Dr. T. Michael Harrington, Chair of Family Medicine at UAB and a large presence in the Family Medicine community in the Southeast, has personal connections with these physicians. The earliest physicians have been engaged to further shape the design of the study, including patient recruitment and intervention finalization.

4.1.b Practice Inclusion/ Exclusion Criteria

All practices located in counties in either AL or NC considered to be part of the Black Belt will be eligible. We recognize that there is no formal definition of the Black Belt, and various sources define the included counties slightly differently, particularly around the borders of the Black Belt.[44] In AL, the Black Belt will include rural counties in the south central part of the state spanning from Mississippi to Georgia. In NC, the Black Belt will include rural counties in the eastern part of the state.

For this project, the <u>practice inclusion criteria</u> are:

- Location in the Black Belt
- Serve a predominately rural population
- High proportion of indigent patients
- High proportion of African American patients
- Internet access at the practice
- Willingness to sign a Letter of Agreement to participate
- Willingness to identify a Practice Champion
- Willingness to modify structure and processes of care with the help of a practice facilitator
- Willingness to work with peer coaches

We will not require EHR implementation for inclusion in the study, because some of the targeted practices have not yet implemented electronic records, and those that have not done so are often also those in greatest need of assistance in achieving BP control. Because the introduction of an EHR requires considerable attention from the physician and practice staff, we will require that EHR implementation is completed at least 6 months prior to participation. We will require that the practice has Internet access, because our data system and the PALS education portal are web-based. In our experience, practices have the highest speed Internet access available in their area.

Practices that do not meet these inclusion criteria will be excluded.

Once a practice meets inclusion criteria and commits to participating, a Letter of Agreement is signed by the appropriate practice official and the practice is placed in queue for readiness assessment, described below in section 4.1.e.

4.1.c Practice Engagement Methods

We use several strategies to engage practices in the Black Belt with sufficient numbers in the sampling frame to assure feasibility (Table 9). Dr. Harrington, a respected leader in primary care in the state of AL and leader of a PBRN leads the AL team's practice engagement, and Drs. Halladay and Cummings lead the NC team's practice engagement efforts. Dr. Cummings leads a PBRN in NC. During the proposal development phase, we secured letters of interest from over 100 practices or practice organizations. Dr. Safford's team engaged over 600 patients from 130 mostly single physician practices in the Black Belt in their work to date, and these practices will be engaged to participate by direct invitation from Dr. Safford. Dr. Harrington will not only approach the AL PBRN practices in the Black Belt, but will also make presentations at local meetings of the Medical Association of the State of AL (see letter of support) and the local chapter of the American Association of Family Physicians. Our and the NC AHEC work extensively with primary care practices in their regions to place medical students in these practices for their ambulatory medicine training, thus they are familiar with many of our targeted practices. They will assist with recruitment as well as providing their qualitative assessment which will inform decision-making on individual practice readiness as part of the Recruitment and Retention Workgroup deliberations. Of note, demand for assistance with practice transformation in both AL and NC is high, as meaningful use criteria are fully implemented and as CMS moves rapidly to value-based reimbursement.

The process of practice engagement is sketched out in Figure 6 and Appendix 1. The process begins with a lead letter and an informational sheet providing an overview of the study (see Appendix 1). Within 2 weeks, a telephone call takes place between a study investigator and the practice physician. The feedback from the Community Advisory Boards and NC AHECs was used to develop talking points for how to respond to potential resistance. The telephone call is followed by additional calls and/or an in-person visit. Once a practice agrees to participate, a Letter of Agreement is obtained from the practice physician or other authorized official, and the practice's Champion is identified (see 4.1.d below). Once the Letter of Agreement is signed, the study team assists the practice with obtaining a Federal Wide Assurance number if the practice does not already have one, and the practice is assessed for readiness to be randomized, as described below in section 4.1.e. If the practice has not previously had a Federal Wide Assurance number, the study team will work with the relevant Institutional Review Board to assure that practice staff participating in the research have received training and certification in human subjects in research.

To assess reach in the RE-AIM framework, we will track the number of practices approached (lead letter sent, initial telephone call completed), the number eventually agreeing to participate (Letter of Agreement signed), the number randomized, and the number completing the intervention period. We will collect information about reasons why practices declined to participate, why they did not move forward to randomization, and why they did not complete the intervention period.

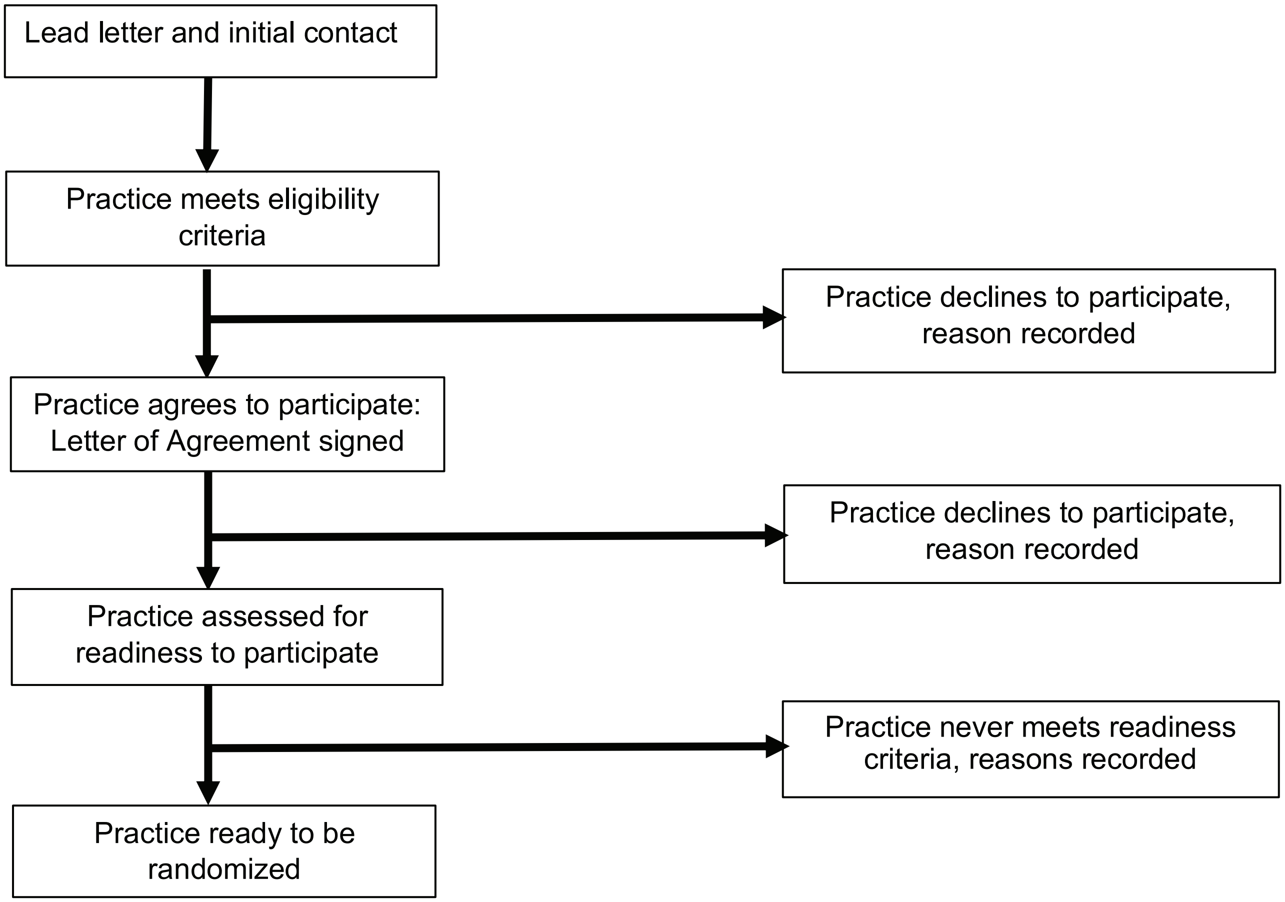

Figure 6. Practice engagement scheme from initial contact through practice readiness for randomization.

4.1.d Practice Champion

A critical requirement for each practice will be a Practice Champion. The Champion agrees to be the point person for communication about the project. Ideal Champions have a vested interest in the success of the project, and are willing to advocate with their fellow practice members to make the project a success. Champions are often nurses but can be anyone on the team. Each practice will be asked to select their Champion.

4.1.e Practice Readiness

A major driver for this proposal is the recognition that some of the highest need populations in terms of uncontrolled HTN and CVD risk receive their healthcare in a wide variety of practices, ranging from single physician practices still using paper records to more sophisticated health center networks actively working on practice transformation. Some variety in practice structure and process is desirable in this implementation context, but too much variation can also create threats to validity. Therefore, based on the NC team's extensive experience with practice facilitation and practice transformation, we have developed practice readiness criteria to assure that a minimum standard is met prior to moving a practice forward to randomization. We have devised a 'speed to readiness program' for those practices committing to participating but not deemed ready by the study team (see below). Furthermore, it is likely that some practices that express commitment initially may experience changes over time. Because the study design calls for waves of practices being engaged over a 2-year period, it is likely that some practices may not be approached for randomization until several months after they signed. Therefore, we will assess readiness on signing the Letter of Agreement, and again just prior to randomization.

In brief, the readiness criteria include:

- Financial stability over the study period and no plans to close the practice in the next 3 years for reasons such as retirement
- Engagement and commitment by the leadership of the practice to support change
- No major disruptions over the study period (e.g., key staff position vacant, key staff member going out on family leave, new EHR being implemented)

The assessment of practice readiness for making structural practice changes is a very new concept and the validation of instruments used to assess various domains of capacity for change is limited. The practice readiness assessment includes items from the Organizational Readiness for Implementing Change (ORIC) scale,[45] which includes two subscales assessing change commitment and change efficacy. The ORIC has acceptable psychometric properties in practices similar to those that will be engaged in the proposed study. A copy of the practice readiness survey (along with the survey of practice characteristics) is provided in Appendix 2.

Readiness is assessed by the Recruitment and Retention Committee, led by Drs. Shikany and Cummings. The data that are considered in determining readiness include:

- Results of the Practice Readiness assessment
- Practice characteristics (assessment tool provided in Appendix 2)
- Qualitative report by the workgroup member who interacted with the practice
- Area Health Education Center (AHEC) team's feedback
- Any additional information known to the study team

These data are considered in making the decision whether to move forward to randomization or whether the practice may need to be placed in the "Speed-to-Readiness" program (described next) and reassessed at a future date. The main driver of the decision is whether the practice is deemed able to complete the year-long intervention, and whether there is agreement or strong agreement on at least 4 of the 8 domains assessed by the Practice Readiness assessment.

The multiple waves of randomization over two years permit practices that are not ready for time-limited reasons, such as implementation of a new EHR, or a staff member away on maternity leave, etc., to enroll at a later date.

4.1.f "Speed-to-Readiness" program

Because some practices may be implementing electronic record systems, or have internal temporary circumstances that may preclude meeting readiness criteria, or have hesitation about participating, we will implement a program designed to help practices meet readiness criteria, hence the name "Speed-to-Readiness". This program is guided by Bandura's social cognitive theory, which posits that practice leaders may learn from others like themselves, and modify their own behavior accordingly.[46] Peer testimonials are effective in modeling results of actions taken by others, a core tenet in social cognitive theory.

In our team's experience with practice redesign interventions, an important impediment to readiness is ambivalence on the part of the practice leaders. Practice leaders are more enthusiastic about peer coaching interventions, which are less disruptive to practice flow. Since a practice has a 50% chance of being randomized to receive the practice facilitation intervention, the peer modeling aspect of the Speed-to-Readiness program will focus on practice facilitation.

We will offer academic detailing for practices in the Speed-to-Readiness phase delivered by Drs. Harrington, Cherrington, Oparil, Cene, Halladay, or Cummings. Practices will receive monthly telephone check-ins by study leaders to assess progress, provide supportive encouragement, and use motivational interviewing techniques to help the practice leaders work towards overcoming barriers to participation and increasing their self-efficacy.

We will create a video of testimonials of practicing primary care physicians who have experience with practice facilitation or peer coaching interventions. We will ask these physicians and their staff to relate their satisfaction with the interventions, to share some of the challenges encountered in implementing the interventions and how they overcame them, and to include the feedback from patients. Practices in the Speed-to-Readiness phase will be engaged in discussions during which we will view segments of the video as a springboard for discussion with ample opportunity for questions.

We may also invite practices in the Speed-to-Readiness phase to join biweekly practice facilitation Collaboratory conference calls at which practices enrolled in the study and receiving the practice facilitation intervention confer about their progress and brainstorm solutions to challenges they are facing. (Practices not receiving the practice facilitation intervention will not participate in this Collaboratory to avoid contamination.) This will permit practices in the Speed-to-Readiness phase to hear first-hand from other physicians and staff similar to themselves about the intervention in near real-time. Practices in the Speed-to-Readiness phase will also be offered a peer mentor in the form of a participating practice for one-on-one discussions and support.

We will evaluate success of the Speed-to-Readiness program by tracking the number of practices that enter the program and the number that achieve readiness to be randomized by the end of Year 3, which will be part of our adoption metric in the RE-AIM framework. We will also track frequency of contacts, participation in the Collaboratory teleconferences, and engagement with the peer mentor. The study investigator making the Speed-to-Readiness calls will take notes during the call and immediately afterward provide qualitative impressions of progress, reporting back to the study team for input and collaborative problem-solving.

4.1.g Practice Compensation

Each of the 80 participating practices will receive a total of $4000. The first payment ($500) is made upon enrollment of the practice, defined as having both a Letter of Agreement signed and randomization completed. Another $1500 will be paid when the 25 patients have been enrolled and have completed baseline data collection, $1000 upon completion of the 6-month follow-up assessment, and $1000 upon completion of final data collection. In addition, each practice will receive a laptop computer with access to the Patient Activated Learning System and 25 home blood pressure monitors, which are theirs to keep.

4.1.h Under Recruitment

Recruitment may be difficult at some practices for a variety of reasons. Every effort will be made to recruit 25 participants at each practice. However, after vigorous attempts over at least 2-3 months, if at least 19 but fewer than 25 participants have been recruited, the program manager and site PI can request an exception to the recruitment target of the Recruitment and Retention Workgroup. This should be an exception and not the rule.

Despite vigorous recruitment efforts, in rare cases, fewer than 19 participants may be recruited over the 2-3 month recruitment period. In such cases, after consultation with the Recruitment and Retention workgroup, recruitment can be stopped at the practice and over-recruitment at other practices may be initiated to make up the shortfall. The guidelines to operationalize over-recruitment are listed below:

1. If a practice has enrolled fewer than 19 participants by 2-3 months, the project manager and PI should obtain approval from the Recruitment and Retention Workgroup to look for same-arm practices for over-recruitment, first at their own site and then at other sites.
2. For every one patient less than 19 recruited at a given practice, the over-recruiting practice must enroll 2 patients. For example, if a practice closes enrollment with 17 enrolled patients, the over-recruiting practice must enroll 4 extra patients to address analytic issues related to power, sample size, and intra-class correlation. The 4 extra patients could be recruited from one single practice within the same arm, or from more than one practice within the same arm (in fact, the second option is preferred from a statistical point of view but not required).

3. Practices cannot be considered for over-recruitment unless they have reached 25 enrolled patients.

Payment for practices that over and under recruit will be modified as follows:

1. A practice that enrolled 19-25 will receive the full payment amount ($1500.00).
2. A practice that enrolls less than 19 patients will be paid per participant at a rate of $60 per participant.
3. A practice that enrolls more than 25 patients may be offered an additional $60 for each extra patient recruited, depending on the relationship between the study team and the practice and the practice's expectation. The decision whether to discuss extra payment with the practice will be at the discretion of the site's program manager and site PI.

It is imperative that all practices recruit at least 19 participants, and stopping short of 25 should be an exception and not the rule. However, in some cases, recruitment may be very slow. If a practice is encountering exceptionally slow recruitment and it has not reached at least 10 patients by 8 weeks, this should be discussed during the Recruitment and Retention Workgroup calls and consideration may be given to dropping that practice altogether. Dropping practices due to slow accrual of participants should be a rare occurrence given the process of selecting only the most promising practices under the purview of the Recruitment and Retention Workgroup.

4.1.i Practice Withdrawal/Dropout Criteria

An occasional practice may need to be withdrawn from the study for a variety of reasons. Some practices may close over the study period, whereas others may modify their structure so that the practice no longer meets eligibility criteria, or enrollment is too slow for the pace of the study.

When a program manager and site PI identify changes at a randomized practice that they feel warrants consideration of withdrawal of that practice, they will present the following to the Recruitment and Retention Workgroup with the proposal to withdraw the practice:

- Description of the change in the practice; e.g., changing to a concierge medicine model, insufficient population, practice closure, staff opposition to participation
- The practice's original eligibility criteria
- Study arm practice was randomized to
- Number of participants enrolled and how far along they are in the study

The Recruitment and Retention Workgroup will discuss the case and render a decision on the practice's status. Depending on the study arm, participants at that practice may be managed differently.

1) Practice Facilitation practices: The intervention will be terminated and retention activities will cease. Participants will be contacted and notified that the practice has been withdrawn from the study. Data already collected may be used in some analyses.
2) Peer Coaching practices**:** Participants will be notified that the practice has been withdrawn from the study and they will be offered the opportunity to complete the intervention if they would like to. For patients electing to complete the intervention, the program manager, site PI, and Peer Coaching intervention Workgroup will assist the study team and peer coach in managing high blood pressure readings on the home monitor that would normally have been communicated to the patient's practice. The following guidelines will be followed:
   - SBP ≥180 or DBP ≥110, no symptoms: coach encourages patient to reach out to their new provider, follows up after the reach out to learn what advice has been given; if patient has not followed up, coach reaches out to study team for assistance.
   - SBP ≥180 or DBP ≥110, with symptoms: coach follows study protocol and reaches out to study team for assistance.
   - Other non-life threatening situations: coach follows study protocol; if the patient has not yet identified a new provider, coach reaches out to study team for assistance.
   - Monthly reports that would normally go to the Practice Champion will be sent to the study team.

   No follow-up data will be collected, with one exception. Participants will be offered the opportunity to join an existing practice in the Peer Coaching arm should one be available nearby. If the participant

chooses to join another Peer Coaching practice, the follow-up data collection schedule will be followed for that practice, and monthly reports will be sent to the new practice's Practice Champion.

4.2. Recruitment of Practice Facilitators

4.2.a Number of Practice Facilitators

Practice facilitators typically manage several practices at once when they work full time. The ideal number of practices is not well defined, but the NC team's experience indicates that no more than 10-15 practices at once is desirable to achieve full engagement and support for the practice facilitator's entire portfolio. For this study, the intervention implementation is staged across a period of several months (see Figure 6, page 15). We will therefore plan on 3.5 FTE facilitators, 2 in AL and 1.5 in NC.

4.2.b Practice Facilitator Inclusion/ Exclusion Criteria

Facilitators will have at least 2 years of experience in a healthcare setting and have high confidence in interacting with busy practices and practice staff. Although not required, an advanced degree in a healthcare field is desirable. Facilitators will be certified through the same program at the University of Buffalo, with additional training provided by the NC Area Health Education Center practice facilitation team. Individuals who meet the inclusion criteria and who are willing to undergo training and commit to the project for at least two years will be eligible.

4.2.c Practice Facilitator Recruitment Methods

Facilitators will be hired by the AHECs in AL under subcontract. They will be responsible for recruitment. As of February 1, 2016, three individuals from AL have entered facilitation training with the University of Buffalo, one of whom expects to be a full-time facilitator. Facilitators in NC will be recruited in collaboration with the NC AHEC or the UNC Chapel Hill team, which collectively have a cadre of certified facilitators active throughout the state.

4.2.d Compensation of Practice Facilitators

Facilitators will be employees of their hiring institutions with salary commensurate with their experience and local hiring practices.

4.3. Recruitment of Peer Coaches

4.3.a Number of Peer Coaches

There are a number of peer coaching models, spanning from full-time community health workers who are employed by a practice on the one hand to volunteers spending a few hours per month providing education or navigation assistance on the other hand. In the Black Belt, FQHC's may have resources to hire a full-time community health worker, but other practices typically do not. For this reason, a model which engages part-time coaches who can work as much or as little as they would like works best. Therefore, the number of peer coaches is contingent on the case load each one of them would like to handle. In our team's past work this has ranged from one client at a time to a dozen or more.

We have engaged and trained over 60 peer coaches for our programs in AL and many have expressed strong interest in being involved in the proposed study. We anticipate requiring 3-5 peer coaches per clinic, thus we will train more in order to assure availability. We plan to recruit peer coaches from each clinic randomized to the peer arm. This is a strength of the program, since for scale-up, a major issue will be how to find suitable individuals to be peer coaches if the peer coaching intervention proves to be effective in lowering BP and improving BP control.

4.3.b Peer Coach Inclusion/ Exclusion Criteria

Peer coaches by nature seek to help others in their community. In keeping with Bandura's social cognitive theory of behavior change, we will seek peer coaches who themselves have high blood pressure, or care for a family member or close friend with high blood pressure. However, they must also be willing to accept help from the research team and community coordinator, especially regarding intervention fidelity. There are additional realities of participating as a peer coach in a research study, such as commitment to the study's duration, willingness to undergo training such as HIPAA and IRB, and willingness to communicate with their client's doctor or nurse. Therefore, to be invited to participate in the study, peer coach candidates must answer "yes" to the following questions:

1. Do you have the desire to help others?
2. Are you willing to accept help?
3. Are you willing to become a ConnectionHealth (AL peer coaches) or Open Water Coaching and Consulting (NC peer coaches) employee or contractor?
4. Do you have high blood pressure or do you help a close friend or family member take care of their high blood pressure?
5. Are you willing to attend and complete the Peer Advisor training?
6. Are you will to work with approximately 5-7 clients over 12 months, by telephone?
7. Are you willing to attend weekly group phone calls with other peer advisors and study doctors?

Of note, the peer coach training and certification programs are excellent filters, with the less committed dropping out prior to completing the program.

4.3.c Peer Coach Recruitment Methods

A key aspect that informs peer coach recruitment is the structure that peer coaching programs are likely to take in the Black Belt region moving forward, as the Affordable Care Act continues to be fully implemented. An important part of this Act is provisions to pay for community health workers, but community health workers are currently not implemented in many practices in the area. In AL, practices are predominately private single or two-physician practices or FQHCs. A community coalition meeting on the topic of peer coach implementation for chronic disease self-management held in Camden, AL in September 2014 engaged over 100 community leaders and revealed that the current fee-for-service model that predominates in AL currently has been a major barrier to implementation of peer coaching programs. This is largely because many of the practices are too small to be able to afford their own peer coach, even in the wake of the January 2015 CMS announcement about the aggressive timeline for transitioning to value-based reimbursement within 2 years. Community members were supportive of a model in which peer coaches worked under community-based organizations (CBO) such as the nonprofit organizations led by our community coordinators rather than in a specific practice. This way, practices (or Regional Care Organizations) could reimburse the CBO for peer coaching services without taking on the human resources management aspects of this type of care extender.

Another key insight during the development of the peer coaching intervention was the need to integrate peer coach into the practice. The peer coach stakeholders that are assisting with intervention development recommended we make efforts to recruit coaches from each of the practices, from among their pool of patients. This increases the likelihood of true integration into the care team, since the person being integrated is already well-known to the practice. We estimate that we will need between 3-5 coaches per practice. A peer coaching program in San Francisco reported that recruiting peer coaches from practices was feasible.[47] We will therefore make efforts to recruit peer coaches from each practice, drawing on our reserve of 65 trained coaches in AL and 15 in NC should recruitment at the practice prove difficult.

In the community capacity building model of community-based participatory research, the Health and Wellness Education Center of Livingston, AL, will be the administrative home for all AL peer coaches, expanding the reach of this CBO and anticipating sustainability on conclusion of the funding period. The Health

and Wellness Education Center and the West Central Alabama Community Health Improvement League will work with the study team and the practices to identify potential peer coaches from among their patients, and to identify suitable coaches from among their pool of trained coaches. The goal will be to identify coaches who can easily travel to participating practices such that travel to the practice will not be burdensome for the coach, since at least one trip to the practice will be required.

In contrast to AL, the NC team members have less experience with peer coaching in collaboration with a CBO. Like in AL, most practices do not currently use a peer coaching model. However, there are a number of peer coaching programs in existence in the NC Black Belt region, as we learned from the NC CAB meeting in February 2016. Therefore, we will partner the coaching coordinator in NC with the coordinators in AL in a mentoring relationship to assure that the structure of the coaching programs in the two states are similar. We will also actively explore partnerships with existing CBO's in NC over the course of the study to plan for long-term sustainability. Coaches will be recruited at the practices as in AL, assuring feasibility of travel to the practice.

4.3.d Compensation for Peer Coaches

Peer coaches in our past studies have expressed strong preferences for a fee-for-service model of reimbursement, which will again be used here. As such, compensation for peer coaches will follow the schedule below:

- Peer coach training consists of 8 courses. Peer coaches will receive $50 for completing each training course, regardless of passing certification for a total of $400
- For each participant:
  - $200 for completing the first 8 program sessions (~2 months)
  - $150 for completing program session 14 (~6 months)
  - $150 for completing program session 20 (~12 months)
  - $25 per month for each additional calls should the participant's blood pressure become uncontrolled with a maximum of 2 months for a total payment of $50

4.3.e Peer Coach Coordinators

A critical aspect of successful peer coaching programs is the ongoing support provided to coaches. This role is filled by the peer coach coordinators. The role of the coordinator is to reach out frequently to the coaches to assure that they have the support that they need, especially social support. Peer coaches greatly enjoy monthly get-togethers with other coaches and the coordinator to discuss challenges, brainstorm solutions, and socialize. The bonds forged in these programs are an important part of satisfaction for the coaches. The coordinator's role is to assess each coach's level of functioning and assure that they remain enthusiastic and committed to the program.

In AL, we have worked with two community coordinators for over 6 years, and these experienced coordinators once again fill this role for the present study. In NC, the coordinator is an experienced peer coach who has worked on previous research studies with the NC team. As with the AL peer coaches, the NC peer coaches will be situated in a CBO led by the coordinator. The coordinators are critical in setting the supportive, collaborative tone and maintaining enthusiasm for the program. The mentoring relationship will assure consistency of implementation across the two states.

4.3.f Peer Coaching Program Session Schedule

The Peer Coaching program is designed to provide an intensive intervention phase, followed by a maintenance phase with boosters as required to keep the participant's blood pressure controlled (see Figure 6A). Because of the implementation context, it is important to recognize that strict adherence to the protocol may not be feasible in some cases. Therefore, we have devised the following goals, which incorporate the real-world issues that often arise and create delays in program implementation.

- Goal: complete session 1 within 30 days of patient signing informed consent

- Peer coach and participant should be matched within 2 weeks of participant is enrolled in the study.
- Session 1 should be scheduled within 2 weeks of the date that the peer and participant are matched.
- Sessions 1 – 8 should be scheduled and completed every 7 days.
- Sessions 9 and 12 should be scheduled and completed every 14 days.
- Monthly sessions should be scheduled every 4 weeks

- Program Goal: complete session 20 by month 12 of baseline enrollment
  - Compress monthly sessions no more than to be 3 weeks apart. If monthly sessions need to be completed <3 weeks apart to keep on schedule, use the following the guidelines below to drop sessions:
    - Sessions 1 - 8 must be completed
    - Sessions 9 – 12 Should be completed
    - If needed - drop sessions 13-18
    - Sessions 19-20 should be completed
- Goal will be to complete session 20 by month 12.
  - 12-month data collection will be scheduled during the 30 days after the 12-month date

If the 20th session is completed before the 12-month date, an alert with will send to the data collection team notifying them that the pt is ready for data collection.

Month 1
- Session 1: Welcome to the program, Blood Pressure Basics
- Session 2: Blood Pressure Medications
- Session 3: Healthy Eating 1
- Session 4: Healthy Eating 2

Month 2
- Session 5: Physical Activity
- Session 6: Stress and Health
- Session 7: Family, Friends, and Health
- Session 8: Getting the most out of your doctor's visits

Month 3
- Session 9: Planning for the future 1 (bi-weekly)
- Session 10: Planning for the future 2 (bi-weekly)

Month 4
- Session 11: Planning for the future 3 (bi-weekly)
- Session 12: Planning for the future 4 (bi-weekly)

Months 5-20
- Sessions 13-18: Monthly calls 1-6 (check-in)
- Sessions 19-20: Preparing for the end of the program 1-2

Special calls: Intensification calls if client needs additional help during sessions 13-20

Figure 6A. Program Schedule for the Peer Coaching Intervention.

4.3.g Contacting Non-Responsive Clients in the Peer Coaching Arm

The goal of this section is to provide guidance regarding participant contacts to peer coaches and program staff. This plan should be tailored for each participant by the peer coach and community coordinator with input from program staff.

Table 9A. Schedule of call attempts by coaches and study staff.

Week	Description
0	Peer and participant matched
1- 2*	Peer makes **frequent** call attempts, varying days and times. *Minimum call attempts: every other day*
2- 3	Community coordinator and program staff begin strategizing ways to reach out to participant (program staff should work with clinic to check contact information, reach out to participants to assess interest in study and verify times/days available, making sure that patient knows the peer's number to ensure they are not screening the peer's calls). Participant is flagged as "at risk."
3-4*	Peer makes **frequent** call attempts, varying days and times. *Minimum call attempts: every other day*
5-8*	Peer makes **intermittent** call attempts, varying days and times. *Minimum call attempt: once/week.*
9	Peer, community coordinator, program staff, data collection staff review non-responsive ppts. *Potential outcomes:*<br>1. Peer coach continues call attempts. A plan for continued peer coach contact is developed at the meeting. Part of plan will be determining a date that the contact plan will be reviewed.<br>2. Participant is given back to program staff.<br>a. Participant will be classified as inactive and peer coaches paid $200.<br>b. Program staff / data collection staff will call *monthly or retention calls* to see if participant can be contacted. If contacted, staff will ask participant if they would like to speak with their peer coach (verifying times available, contact info, and give peer's number)<br>c. If patient is scheduled for 6-month data collection, staff will ask ppt if they would like to work with peer coach (verifying times available, contact info, and give peer's number).<br>d. Participants will be returned to peer coaches if participant requests peer calls to resume. At this time, ppt will be re-classified as active and peer calls resume. *See Revising program session schedule protocol.*

*** Peer coach should be in frequent communication with community coordinator, who, with help of program/data collection staff, will work with peer coach and practice staff and try different methods to contact participants.**

4.3.h Peer Coaching Participants at Risk of Not Completing Intervention and Dropouts

Information on when a peer coaching participant is hard to reach, missing sessions, has suffered a significant illness or life event, or other situation which would cause the patient to miss coaching sessions or become unresponsive to coach calls can come from many sources. This information may be obtained from retention calls, six month and 12 month interviews, speaking to peer coaches or coordinators, or coaching monthly reports. The following are guidelines on what to do once an investigator or a research assistant or data collector identifies a peer coach participant who would like to drop out from the peer coach arm:

1) Program Managers should be notified, and supplied with all the information, including if possible, the peer coach call logs showing all the calls the coach made to connect with the patient. This information should be entered into the peer coaching database in *ClinvestiGator*, and the patient marked *"*At Risk.*"*

2) At Risk Participants: Patients can be considered at risk of not completing the peer coaching program for any reason. It can be a temporary status, or it can lead to eventual peer coaching intervention withdrawal.
    1. Program staff should work with coaches and practice champions to make sure the patient contact information is up to date.
    2. Peer Coaching protocols should be followed with the minimum number of calls to the patients, texting etc… and documented in the peer coach workbooks for that patient.
    3. If a patient continues to be unreachable or tells study staff or their peer coach they wish to withdraw from the intervention, the appropriate project manager should be notified. All Non-responsive patients should be referred to program managers. Program managers should make one last attempt to reach the patient and make sure the patient is aware that they can continue in the study, and withdraw ONLY from the coaching intervention. As needed the PM will mark patient as Withdrawn from Peer Coaching.

3) Patients Withdrawn from Peer Coaching
    1. Patients who choose not to participate in peer coaching are still enrolled in the study. They should be continued to be tracked, and attempts to complete the follow-up data collection should be made.
    2. In addition, once a patient is withdrawn from peer coaching, the alert banner will automatically show up on the patient dashboard each time the patient record is opened in *ClinvestiGator*.
    3. All staff who have contact with this patient record will see this alert, and check to see if the 'withdrawal from peer coaching' event form has been filled out. This form is located at the very bottom of the patient database in *ClinvestiGator* called "Withdrawn from Peer Coaching Intervention."
    4. All staff who have contact on the phone, or at a 6 month or 12 month interview will try to complete the form; only one is needed.

5. Recruitment of Subjects

5.1 Number of Participants per Site

This study will include a total of 2,000 African American participants. Participant recruitment will take place in participating practices, and 25 patients with uncontrolled hypertension per practice will be recruited. Because some practices in the Black Belt are very small and we wish to optimize generalizability, we will accept a total of 23 participants if recruitment has been ongoing for at least 3 months and the full target of 25 participants has not been recruited by that time point.

An important aspect of a pragmatic trial is the ability to examine the heterogeneity of treatment effects in high-risk subgroups of interest that are often difficult to recruit into research studies. We preplan subgroup analyses on 1) men, 2) those younger than age 60 years, 3) those with low health literacy/numeracy, and 4) those reporting mild or greater depressive symptoms. Past reports have shown that male sex and younger age are associated with higher risk for uncontrolled CVD risk factors, and health disparities are also greatest in younger individuals.[2,11] Low health literacy, numeracy and depression have also been shown to be associated with uncontrolled CVD risk factors.[48-52] We will therefore recruit at least 10 men and 12 individuals younger than 60 years of age at each practice; based on our past trials, we know that mild depressive symptoms are present in 50% of this population, and health literacy is also very low. The background prevalence and oversampling of men and younger individuals will provide enough power to conduct these pre-specified subgroup analyses (see also Analysis Plan, section 8).

5.1.a Participant Inclusion/ Exclusion Criteria

Inclusion and exclusion criteria are shown in Table 10. Concordant with the pragmatic trial context, inclusion and especially exclusion criteria are kept to a minimum to maximize generalizability. In order to minimize regression to the mean and to select patients at highest risk, an important goal for our partnering practices, we will select individuals with uncontrolled hypertension, defined as a mean systolic BP over the previous year at least 140 mm Hg plus an enrollment day BP ≥140/90 mm Hg as assessed by the research assistant.

Table 10. Patient inclusion and exclusion criteria for the trial.

Inclusion criteria	Exclusion criteria
• *African American adults aged 19-85 years*<br>• *Uncontrolled HTN, defined as ≥140 mean systolic BP over the past year AND BP≥140/90 mm Hg at the time of study enrollment*<br>• *Black Belt resident*<br>• *English speaking*<br>• *Telephone available*	• *Plans to move out of the area within the next two years*<br>• *Advanced illness with limited life expectancy*<br>• *Chronic kidney disease with eGFR ≤ 45 ml/min/1.73 m^2*<br>• *Unwillingness to work with a peer coach*<br>• *Unwillingness to sign informed consent*<br>• *No telephone available*

Because the control of BP in people with advanced chronic kidney disease (CKD) is more difficult and requires special approaches, we focus this study on more average patients with estimated glomerular filtration rates (eGFR) greater than 45 ml/min/1.73 m^2. We use 45 as a cut point to maximize reach without selecting a population that requires management that is too different than that for more average patients. We considered 60 mL/min/1.73 m^2, which is the threshold for Stage 2 CKD, but this threshold may exclude too many individuals in this pragmatic setting. In the national REasons for Geographic And Racial Differences in Stroke cohort study comprised of over 30,000 African American and white community dwellers aged 45 and over at baseline, 4.2% of African Americans had eGFR mL/min/1.73 m^2. However, another 7.1% of African Americans had eGFR between 45 and 60 mL/min/1.73 m^2, which would exclude one in 10 prospective participants. Based on input from Dr. Oparil, a well-respected expert in the field of HTN, the threshold of 45 mL/min/1.73 m^2 is a reasonable threshold for the study, balancing inclusivity with the need for simplicity in the treatment algorithm, which was updated to reflect the new HTN guidelines.

5.2 Withdrawal Criteria

Because of the pragmatic design, this study will not have withdrawal criteria. However, over the course of the study, a participant may develop an exclusion criterion (e.g., become pregnant); in this case, the participant may be withdrawn from the study by the Recruitment and Retention Workgroup.

5.3 Participant Recruitment Method

Participant recruitment approaches will be adapted to individual practices needs. Recruitment materials (posters, pamphlets) are displayed prominently in each practice (see Appendix 3), along with a certificate of the practice's participation. The practice staff led by the Champion encourage patients to consider participating; those who express interest fill out an interest card that includes the patient's contact information and signature indicating willingness to be contacted by the study team.

Our experience shows that despite enthusiastic support for the study, practice staff members rarely refer many patients because they have too many competing demands.[15] Therefore, a study research assistant schedules a time to visit the practice to conduct in-person recruitment with the help of the practice's staff in accordance with HIPAA regulations and guided by our respective institutional review boards. In AL, recruitment staff members are African Americans from the Black Belt, lessening mistrust as a barrier to participation, and the NC teams similarly will strive to have African American staff. Since the prevalence of HTN is so high in the targeted population, and more than half of African Americans are not controlled, each practice is given 1-3 months to recruit the target number of 25 patients. If a practice is not able to meet the target number of patients in the given time period, the site research team should refer to section 4.1.h for guidance.

5.4 Participant Screening and Consent

The number of patients with uncontrolled HTN approached for participation is tracked as part of the Reach metric of the RE-AIM framework by the research assistant stationed at the practice for recruitment purposes. These data are entered by the research assistant into the study's web-based data system.

Once a participant has expressed interest, the study research assistant screens the prospective participant. For eligible prospective participants, the research assistant then provides an explanation of what participation entails:

- Completing a baseline in-person assessment at the practice,
- Completing a baseline telephone interview,
- Providing several alternative telephone numbers to facilitate contact,
- Completing 6-month and 12-month follow-up assessments,
- Using a home BP monitor, and
- Possibly working with a peer coach.

The research assistant also explains randomization, which occurs at the practice level. Willing participants then provide informed consent, which is explained and read to the participants by the research assistant. The study research assistant answers any question the participant may have, and the participant then signs the informed consent (see Appendix 4). A copy of the consent form is provided to the participant. Only trained and quality controlled research assistants provide the information required to obtain informed consent.

5.5 Participant Compensation

Participants are expected to complete 3 assessment visits over one year. They will receive $40 per visit for a total of $120.

5.6 Coordination of Practice Recruitment, Peer Coach Recruitment and Training, and Participant Recruitment

The recruitment of practices, coaches, and participants must be carefully coordinated. The goal of the study is to have all participants exposed to the interventions for as close to one year as possible. However, for the practice facilitation intervention, it can take up to 3 months to complete self-assessment, identify and plan

for the first activity, and finally implement it. If participant recruitment begins on randomization, the participants recruited earliest could have considerably less exposure to actual practice change than those recruited later. Similarly, coaches ideally are identified from the practice, followed by 2-3 months to complete training. For practices receiving both interventions, participants should be recruited only once the practice is ready to start on its first activity *and* the coaches have been trained.

In order to address these issues, there will be a 3-month run-in phase after practice randomization and prior to participant recruitment, as shown in Figure 7. Practices that meet readiness criteria will be batched into stages for randomization 3-6 months prior to anticipated start of patient recruitment.

Run-in activities for those practices randomized to the coaching intervention include:
(1) identifying coaches who can travel easily to the practices, followed by
(2) training any new coaches.

Run-in activities for those practices randomized to the practice facilitation intervention include:
(1) establishing rapport,
(2) conducting the self-assessment,
(3) providing an overview of the intervention,
(4) helping the practice to select their first activity, and
(5) planning for the implementation of that activity.

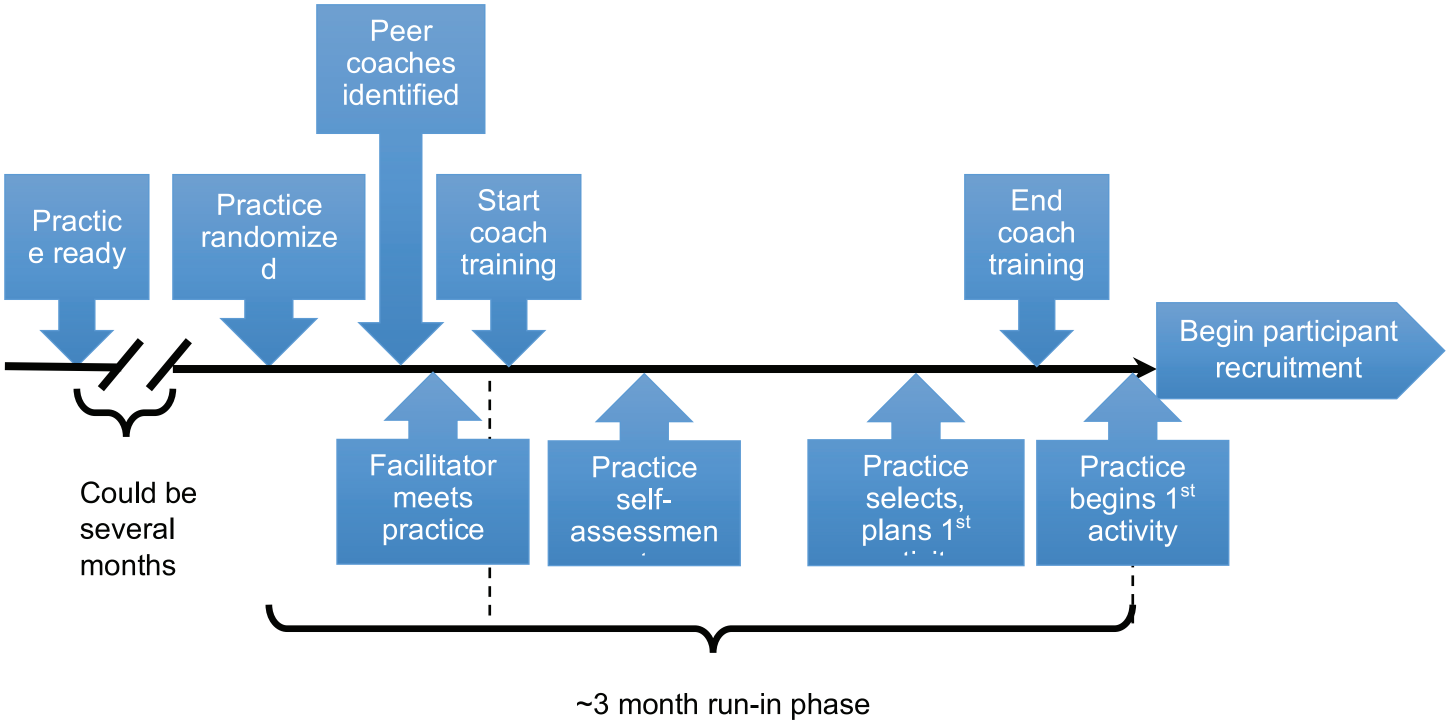

Figure 7. Run-in phase between practice randomization and start of participant recruitment. Schematic shows activities for the 20 practices that receive both practice facilitation and peer coaching. Those practices randomized to the coaching-only arm would not receive the practice facilitation run-in activities. Those practices randomized to the practice facilitation-only arm do not receive the coaching run-in activities. Schematic not to scale.

6. Interventions

6.1 Conceptual Framework

The Affordable Care Act's provisions for alternative models of healthcare (like practice transformation and community health workers) along with CMS's recent announcement that they will move to mostly value-based reimbursement within 2 years together create a unique opportunity to study these two components alone and in combination in a very high-need region of the US from the perspective of HTN. This study will implement programs that will be designed with practices, peer coaches, and patients with HTN residing in the Black Belt, assuring that the interventions are sustainable and scalable throughout the region.

For the multi-level practice facilitation intervention, we draw on Donabedian's structure-process-outcomes paradigm and Wagner's chronic care model.[53-56] Donabedian proposed that healthcare systems should be structured to facilitate the provision of high quality care. This long-standing paradigm has guided quality improvement and health system design for decades, and is now being applied at the individual practice level. The chronic care model posits that care should be patient-centered and responsive to individual needs and preferences, drawing on community resources to optimize health outcomes. Practice facilitation is specifically designed to restructure practices and introduce new processes of care with an emphasis on patient-centered, proactive care. Practice facilitation in this intervention will help practices to fulfill 4 key functions:

1) **Team-based care** that engages members of the healthcare team other than the physician for BP medication management (structure and process change);
2) **Self-management support** through home BP monitoring and engagement of community resources (chronic care model);
3) **Standardized Care Process**, for example through telephone management (structure and process change);
4) Providing **outreach,** based on audit and feedback reports generated from HTN registries (structure and process change).

In this functional intervention, facilitators will assist practices in making highly tailored changes in structure and process to accomplish these 4 functional goals, aiming to improve BP control, health, and patient satisfaction.

Despite emphasis on patient-centered care, practice facilitation focuses on processes of care that address low uncertainty clinical situations, such as titrating BP medications when BP is not controlled. However, more difficult patient behavior modification may respond more to the relationship-focused peer coaching intervention, since behavior change is complex and filled with uncertainties. Bandura's social cognitive theory posits that we learn by watching others like ourselves and underlies the effectiveness of peer coaches, who are "someone like me with problems like mine."[46] Peer coaching is another functional intervention, fulfilling 3 key relationship-based functions:

1) **Assisting** clients in carrying out the care plan;
2) **Supporting** clients emotionally, establishing meaningful, supportive relationships, and encouraging them to take an active role in their healthcare; and
3) **Linking** clients into care and community resources.

Adult Learning Theory posits that adult learners are internally motivated, goal and relevancy oriented, and highly practical.[57] They also bring life experiences and knowledge to learning experiences, and they like to be respected. By interacting with another community member who understands and supports them, patients share experiences and barriers to behavior modification. Importantly, our formative work shows that they prefer to interact with the peer coach for health-related questions, allowing the peer coach to be the link to the healthcare team to find answers together. In the peer coaching functional intervention, the relationship is the

operator to accomplish the 3 key functions, aiming to improve BP control and satisfaction, but through different influences compared with practice facilitation interventions.

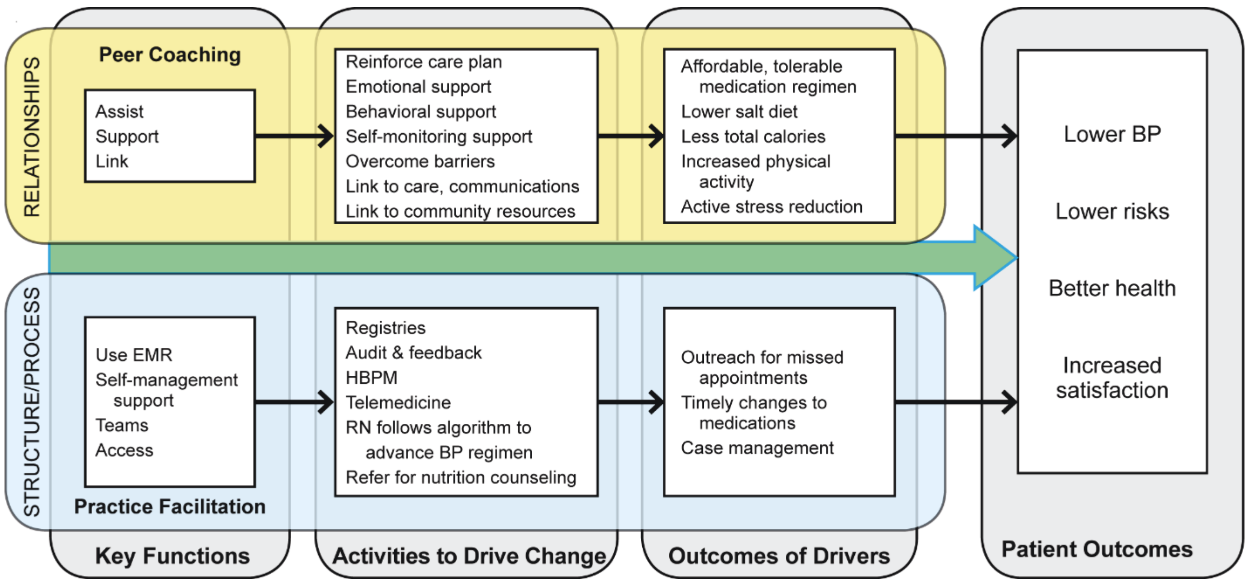

Figure 8. Conceptual framework for the study. BP = blood pressure. EMR = electronic medical record. HBPM = home BP monitoring.

We propose that both structure/process improvement and strong relationships are needed to overcome distance, trust, and psychosocial barriers to optimize BP control among African Americans living in the Black Belt region. We anticipate that both practice facilitation and peer coaching will have clinically important effects on BP, but that together they will generate a greater improvement in BP control trough their complementary pathways of influence (Figure 8). As healthcare reform moves forward, the results of this study will inform policy decisions at the regional and local levels to help communities and practices in vulnerable regions like the Black Belt to decide which approach is right for them, informed by how much effort is required for each intervention, and how much benefit they can expect to derive from implementing either practice facilitation or peer coaching, or both.

6.2 Implementation Framework: RE-AIM

Because this is a pragmatic trial seeking to test scalable, sustainable approaches to improving HTN control in the Black Belt, we use an implementation framework to inform other Black Belt practices as they consider the results of the study and implications for their own patients. A widely used framework is Glasgow's RE-AIM, which stands for Reach, Efficacy/effectiveness, Adoption, Implementation, and Maintenance of the intervention effects.[58-60] We integrate the framework throughout the project as shown below.

RE-AIM Domain	How accomplished	Section in protocol
Reach	Track the number of participants approached for participation, compare with the number enrolled	4
Effectiveness	Baseline and follow-up BP assessments	7, 8
Adoption	Track the number of primary care practices approached, compare with the number agreeing to participate	4
Implementation	Intervention fidelity	6.6
Maintenance	1-year follow-up calls to practices	7.d

6.3 Intervention Approach/ Description

6.3.a Enhanced Usual Care

Enhanced usual care will be the comparator for the study. All practices receive enhanced usual care regardless of randomization status, which includes:

1) A laptop computer.
2) The freely available web-based PALS education system. Patients and practice staff will be able to access evidence-based information using the PALS question-and-answer searchable system using a user name/logon ID to track utilization. This system offers short, engaging content including liberal use of video clips to provide education.
3) 25 home BP monitors and BP logs that are theirs to keep on conclusion of the study.
4) A binder of practice tips that will include flow sheets and the same BP medication advancement algorithm used in the practice facilitation and peer coaching interventions (Appendix 6).

Past research has shown that education and simply providing practices tools without more intensive intervention does little to change outcomes.[61-63] Nevertheless, the practices we have engaged while preparing this proposal requested some sort of program regardless of the arm of the trial, and novel education materials plus home BP monitors was acceptable to these stakeholders in this real-world setting. Our experience with community-based interventions also indicates that "control" arm participants strongly prefer some sort of program rather than only usual care. Therefore, enhanced usual care directly responds to requests from stakeholders while creating little chance of compromising the ability to observe control-intervention differences and aligning with **PCORI Methodology Standard 1:RQ-6** which suggests that researchers "select outcomes based on input directly elicited from patient informants and people representative of the population of interest, either in previous studies or in the proposed research".[1]

6.3.b Practice Facilitation

6.3.b.i Practice Facilitation Intervention Development

Practice facilitation is a highly customized, staged approach to helping a practice to implement process and structural changes to enhance the quality of care and improve patient and staff satisfaction. Facilitators are certified to have 4 core competencies:

1) Interpersonal skills to build support for and facilitate change;
2) Methods for accessing and using data to drive change;
3) Quality improvement and change management strategies; and
4) Health information technology optimization.[24]

They build rapport and trust with the practice team, and they work together to assess their practice, and decide what process and structural changes would create the greatest chances for achieving the desired outcome, in this case, improving BP control in their patients. As such, practice facilitation is a *functional* intervention that naturally steps up efforts based on data-driven feedback reports from the practice itself. Practice facilitation is often focused on improving a key quality metric such as HTN control. Based on the evidence, practices will implement 4 key functions, including:

1. Team Management: Medication titration algorithm implemented by a nonphysician. We anticipate that many practices will implement telephone-based medication titration using the patient's home blood pressure measures. Implementing an algorithm that advances intensity of treatment has been shown to improve BP control in a variety of settings and studies, many of which have used nurses or pharmacists to titrate the doses.[41,64,65] Physicians often do not intensify the regimen even after several encounters with patients with uncontrolled BP,[66,67] practice facilitation will result in another healthcare team member such as a nurse taking over algorithm-guided medication titration (Table 11 and Appendix 6). The algorithm used

here is guided by the 2010 Consensus Statement issued by the International Society of Hypertension in Blacks, the JNC8, the 2017 AHA Guideline for the Prevention, Detection, Evaluation, and Management of High Blood Pressure in Adults, as well as the recently concluded SPRINT trial algorithm (see Appendix 6).[42,68,69,96]

An important aspect of this algorithm is the attempt to institute chlorthalidone (CHLD) therapy, which is the diuretic used in the major clinical trials of HTN. In practice, physicians more often use hydrochlorthiazide (HCTZ) because it is less expensive and causes less hypokalemia. However, Dr. Oparil's 30+ year experience in the UAB Hypertension Clinic show that CHLD is a more effective antihypertensive agent: an observation supported by a wealth of literature. When used in doses of no more than 25 mg, CHLD is an extremely safe medication which does not cause the profound hypokalemia seen with doses of 50 mg or 100 mg. Dr. Oparil therefore advises that CHLD be utilized in all African Americans with uncontrolled hypertension. She also advises against the indiscriminate use of angiotensin converting enzyme inhibitors (ACEI) in African Americans, citing an ineffective BP lowering profile and a higher incidence of angioedema compared with angiotensin receptor blockers (ARB): she recommends that ACEI and ARB only be used for the patient subpopulation with chronic kidney disease (CKD), defined as eGFR < 60 mL/min/1.73 m^2, and that under these circumstances ARB should be preferentially employed. Additionally, she recommends we avoid the use of beta blockers, which have little BP lowering effect in African Americans and have a high side effect profile. Beta blockers can, however, be used for other guideline indications, namely in the 3 years following a heart attack or in the setting of systolic heart failure. Finally, she contends that loop diuretics are not to be used as antihypertensive agents and that they should be used only if there are other indications such as systolic heart failure. The algorithm has been formulated to incorporate all of these recommendations.

An important consideration in this trial is the likelihood that most of the trial's participants will be living in low socioeconomic circumstances, and cost will likely be a considerable issue for them. For this reason, we will include in the algorithm less expensive alternatives like HCTZ. However, we will recommend the medications with the most robust evidence of efficacy as first line, and those with less robust evidence as second line.

Similarly, we recognize that pill burden may be substantial in many of the trial's participants because of the well-known clustering of cardiovascular risk factors in African Americans, especially those living in low socioeconomic circumstances. Therefore, we will encourage the use of fixed-dose combination pills, several of which are low in cost. We will also include extensive relative cost information which will be as locally relevant as possible.

Table 11. BP Management Algorithm for Medication Intensification for Uncontrolled BP (see Appendix 6)

Current Therapy	Options if BP Uncontrolled
No medications	*Start 12.5 mg chlorthalidone (if diabetic or eGFR 45-60 mL/min/1.73 m^2, add ACEI or ARB (prefer ARB); if BP ≥ 160 mm Hg, start 25 mg chlorthalidone)*
HCTZ monotherapy	*Switch to chlorthalidone (if diabetic or eGFR 45-60 mL/min/1.73 m^2, add ACEI or ARB (prefer ARB)*
ACEI or ARB monotherapy*	*Add 12.5 mg chlorthalidone (25 mg if BP ≥ 160 mm Hg) and switch ACEI to ARB*
DhCCB monotherapy	*Add 12.5 mg chlorthalidone (25 mg if BP ≥ 160 mm Hg) (if diabetic or eGFR 45-60 mL/min/1.73 m^2, also add ACEI or ARB, prefer ARB)*
HCTZ + ACEI/ARB	*Switch to 12.5 mg chlorthalidone (25 mg if BP ≥ 160 mm Hg) and increase dose of ACEI or ARB (if not already at maximum dose), or add dhCCB*
HCTZ + maximum dose dhCCB	*Switch to 12.5 mg chlorthalidone (25 mg if BP ≥ 160 mm Hg) and add ARB*
ACEI/ARB + maximum dose dhCCB	*Add 12.5 mg chlorthalidone (25 mg if BP ≥ 160 mm Hg), change ACEI to ARB*
ACEI/ARB + dhCCB + thiazide-like diuretic	*If all at maximum dose, consider adding 25 mg spironolactone*
4-5 agents, all at maximum tolerated doses	*A study Clinical Hypertension Specialist will provide recommendation*

ACEI=angiotensin converting enzyme inhibitor. ARB=angiotensin receptor blocker. CCB=calcium channel blocker. dh=dihydropyridine. HCTZ=hydrochlorothiazide.*ACEI and ARB are not to be used together in any regimen. †Equivalent doses of chlorthalidone are more potent and longer-lasting than HCTZ. If cost of chlorthalidone is a barrier to adherence, consider using indapamide.

Both Drs. Oparil (in Alabama) and Viera (in North Carolina) run HTN clinics at their hospitals. Questions about care for truly resistant HTN will be directed at them for tailored care.

2. Self-management support has evidence for modest benefit.[70] A 2005 meta-analysis of RCT included 13 studies of HTN, and concluded that there was modest benefit (~5 mm Hg reduction) of self-management support, and earlier studies supported this conclusion.[71] Self-management support can take the form of group classes, educational materials for self-administration, one-on-one sessions in the clinic, goal-setting, among others.[72] Practice facilitators will customize self-management support based on the practice's preferences and local feasibility. Practice facilitators will also train practice staff on how to use the PALS with their patients; the PALS includes engaging educational materials on HTN, BP medications and side effects, healthy eating in accordance with the DASH diet and modified to Southern culinary tastes, and physical activity.

An important multi-level aspect of the self-management support function is tapping into community resources, which include nutrition counseling through the Department of Health and the Cooperative Extension. Additional community resources include farmer's markets, community gardens, walking groups, and church-based health programs. To facilitate community linkages, Practice facilitators will train practices how to use mydiabetesconnect.com, a diabetes-related resource bank for the Birmingham area developed by UAB investigators. This site has been expanded into the AL Black Belt counties, and we are further expanding this website for the NC Black Belt communities surrounding our partnering practices, drawing on community resource guides developed by the NC team,[73,74] as well as suggestions from patients, peer coaches and practice staff (see section 3.1.b above). The web site includes local community resources that practice staff will be able to use to help patients with self-management support.

Additional self-management support will be through home BP monitoring. A 2011 systematic review of 37 RCTs concluded that there was evidence of modest benefit, especially when combined with telephone support, as will be done here. Home BP monitoring is endorsed by the Million Hearts campaign[75] and JNC8.[42] Practice facilitators will teach practice staff and physicians how to use home BPs and how to train patients in the use and reporting of home BP readings. Participants will use their home blood pressure measures to reinforce the need for medication adherence, medication titration, and lifestyle changes, and will report home blood pressure measures to practice staff during telephone management calls. Practice staff will use home blood pressure measures to make treatment decisions and to reinforce messages on medication adherence and lifestyle adherence.

Cultural sensitivity is an important aspect of successful BP management in the targeted population. Patients living in poverty are often embarrassed by their inability to follow physician recommendations due to limited resources or confusion and lack of understanding. They will not readily disclose nonadherence for these reasons. Part of the study-specific practice facilitation training will include training in cultural sensitivity, based on numerous workshops conducted at UAB under the auspices of its Minority Health Research Center. Drs. Safford and Cherrington have both led such workshops.

3. Improved access. Distances combined with poverty create large access barriers in rural areas like the Black Belt. Practices will overcome access barriers by expanding telephone management based on home BP readings if patients cannot attend clinic. As value-based reimbursement is fully implemented, past barriers to providing more telephone advice and support, which is not reimbursed in AL, will be removed. Practice facilitators will assist the practices in developing protocols for telephone-based management.

4. Outreach through use of the EMR: registries, audit and feedback. Facilitators assist practices in the meaningful use of their EMRs, where present, to create a registry of their HTN patients, with audit and feedback reports to identify individuals who are uncontrolled, and/or have missed appointments. These reports are used for outreach and to re-engage individuals at high risk for uncontrolled HTN. Visit frequency is directly correlated with BP levels.[76] Outreach can include sending reminder cards or telephone calls. Audit and feedback reports also prompts plan-do-study-act cycles for improving processes that the practice and the facilitator identify as barriers to achieving the goal of 75% HTN control in their HTN patients.

Each practice randomized to receive the practice facilitation intervention will work with their facilitator to implement these 4 core functions. Similar to the peer coaching intervention, the specific activities that will be undertaken to accomplish this implementation will vary from practice to practice. For example, in practices without EMRs, their billing and scheduling systems may be used to create registries and identify patients for outreach. Such practices may need to conduct chart audits to create their feedback reports rather than learning how to extract reports from their EMR. Similarly, the team member identified for implementing algorithm-based BP medication management may vary from a nurse to an experienced nursing assistant, depending on the makeup of the practice team.

Although the specific activities across practices may vary, each facilitator will be trained to encourage the implementation of at least one activity in each of the 4 key areas over the 1-year intervention period. We anticipate that all practices will work toward developing a data-driven approach to improvement, to consider having some form of telephone management using home BP readings in place, to implement the algorithm to guide BP medication titration (Appendix 6), and to enhance self-management support. Some practices may implement several activities under each key function, but all will be asked to implement at least one under each key function.

An important aspect of this intervention is the successful engagement and activation of African American patients. While all of these practices have extensive experience with African American patients, emerging evidence suggests that even experienced practitioners may not utilize approaches and practices that maximize patient engagement. For example, although cost is a barrier to medication adherence, many patients do not feel comfortable raising this issue with the provider, and few providers systematically ask about affordability of the treatment regimen. Doing so in a supportive, culturally sensitive manner enhances the practice’s ability to assure that all of its patients are on an affordable medication regimen. Cultural sensitivity will be a key focus of practice facilitation training to assure that all of the activities undertaken by the practice are implemented in a culturally concordant manner.

The current draft of the practice facilitation guide is appended in Appendix 7.

6.3.c Peer Coaches

6.3.c.i Peer Coaching Intervention Development

The peer coaching intervention is modeled on lifestyle and medication adherence interventions we have developed for diabetes in collaboration with community partners now spanning over 6 years. The peer coaches prefer structured programs, thus we use that approach here.

We use a partnership approach to intervention development. We have learned from our participants that they greatly value lifestyle modification in any health-related intervention, as reflected in the fact that 93% of Encourage study participants chose to work on diet and exercise to improve their diabetes “numbers” rather than working on medication adherence. Responding to this request, the intervention includes chapters on healthy eating and exercise to enhance engagement.

The intervention is developed iteratively in partnership with existing peer coaches. We draft key evidence-based content elements for each chapter and our peer coaches provide feedback, which is then incorporated. We then create a full draft of the chapter and tape record the interaction between a mock peer coach and a patient. The peer coaches listen to the recording and view the fully developed chapter, and again provide feedback. They then practice with each other in pairs until they are ready to be certified on the chapter by playing the role of the peer coaches with one of our research assistants playing the role of the patient. We repeat this process weekly until each peer coach has been certified; for an 8-session program, this takes about 10 weeks. We anticipate 8 chapters for the structured phase of the intervention as shown in Table 12, followed by booster contacts at least once per month for a full year.

Table 12. Peer coaching intervention topics and timing

Topic	Overview; what is HTN	BP meds, home BP monitoring	Healthy eating 1	Healthy eating 2	Physical activity	Stress and Health	Family, friends, and health	Getting the most out of MD visits	Planning for the future and Booster sessions + intensification calls as needed
Timing	*Week 1*	*Week 2*	*Week 3*	*Week 4*	*Week 5*	*Week 6*	*Week 7*	*Week 8*	*Week 9 and ≤Monthly*

The interventions include engaging video presentations with visual images and few written words instead of didactics, and embed community members telling their perspectives and stories to personalize the intervention, and to incorporate Adult Learning Theory and Social Cognitive Theory. The educational material is presented on DVDs, since many community members do not have access to the internet. Participants are asked to watch the week's chapter prior to their telephone call with the peer coach. During the call, they discuss the content of the video and the peer coach reinforces key points. The participant is given an activity book, and the peer coach has a peer coach manual and a client plan book, both of which are coordinated to facilitate the discussion (see Appendix 7 for examples of a chapter in an Activity Book and coordinated coaching manual from an ongoing study that will be adapted here). The participant is given homework at the conclusion of each session, which they track in their Activity Book and which is reviewed at the beginning of the next session. The peer coach helps the patient to set goals around self-management, including medications, home monitoring, and diet and exercise, and she helps the patient to strategize how to accomplish the goals, using motivational interviewing techniques.

Peer coaches may also use the expanded mydiabetesconnect.com website developed at UAB to link their clients into community resources. An attractive feature of the website is the peer coach's (or practice staff's) ability to enter new resources or make corrections to existing listed resources, such as a change in hours or location. The website thus serves as a dynamic resource, constantly being updated by its users. NC also has web-based links to community services that will be emphasized as a resource during training. Furthermore, during the peer coach trainings, the peers as a group complete an asset mapping activity to help identify and share additional resources located in the clinic's community. These are added to mydiabetesconnect.com.

An important component of the peer coaching intervention is linking back to the practice about the patient's progress. Each peer coach will meet the Practice Champion in person at the start of the intervention period to establish rapport. Practice Champions are briefed on the value of peer coaches and a synopsis of the evidence supporting peer coaching interventions in HTN. Each practice will tailor their communication approach with peer coaches, whether by telephone or in person visits to the practice, assuring timely communication of progress and issues that arise during the interactions between the patient and the coach. For example, if the patient discloses that they cannot afford the HTN medication regimen, the peer coach will notify the practiced through the agreed upon communication channel, and the physician will review the medications and recommend an affordable regimen that the peer coach then assures is received by the patient. The study provides a resource for medication price comparisons for practice staff through the PALS web portal. The coach reaches out to the practice every two weeks during the first 2 months, then monthly, using a structured form to report on progress. This form will include home BP readings, which will be used by the peer coaches to assess progress, reinforce educational messages, reinforce the importance of adherence to medications and lifestyle changes, and during the maintenance phase, they will use these measures to assess the need to intensify contacts if blood pressures begin to rise.

6.3.d Hybrid Intervention: Practice Facilitation with Integrated Peer Coaches

The 4th arm of the trial combines practice facilitation with peer coaches. During the intervention development and practice engagement process, it became clear that practices were enthusiastic about the peer coaching intervention and how it could be integrated into the practice facilitation intervention. For

example, practices reported difficulties in getting their patients to understand medication titration instructions, and they identified a role for the peer coaches in which they would assist patients with implementing these instructions, providing the extra help and reinforcement to assure that the patient understands what they are supposed to do. This was felt to be especially critical for telephonic medication management. Another area where our partnering practices felt peer coaches could be instrumental was in providing self-management support, especially with lifestyle changes. Based on extensive discussions with our partnering practices, it is likely that peer coaches may be integrated into the activities in several of the 4 Key Driver domains, making it difficult to tease apart the effects of the practice facilitation intervention and the peer coaching intervention. Practices were also enthusiastic about the peer coaches assisting with outreach, especially if telephone numbers change and the practice has difficulty reaching the patient. They looked forward to having the peer coach be tasked with obtaining new contact information and encouraging the patient to keep follow-up appointments. For these reasons, it is likely that the combination of the two interventions – practice facilitation and peer coaching – will result in an intervention that is more than a simple sum of the two interventions.

Practice facilitators and peer coaches will receive the same training for this hybrid intervention, but the practice facilitator change packet will add examples of activities that integrate peer coaches such as those listed above.

6.4 Training

6.4.a Facilitator Training

We will certify facilitators using an AHRQ-funded certificate program available through the State University of New York at Buffalo/Millard Fillmore College. This web-based program spans 13 weeks and includes 1.5 hours/week of webinars, plus 26 hours of reflective learning. The certification program is designed to build competency in interpersonal skills, accessing and using EHR data, quality improvement strategies, and health information technology optimization. A 40-hour practicum experience with another certified facilitator is also required. The NC AHEC program has trained several certified facilitators, who will provide practicum experience for the project's facilitators, also serving as mentors for the AL AHEC program, which currently does not have any certified facilitators. AL facilitators will be part of the AHEC program, building a work force of facilitators for the state of AL, and laying the groundwork for a sustainable practice facilitation program.

Once certified, facilitators receive additional training to provide study-specific skills. They receive training on HTN and its treatments, become familiarized with the HTN treatment algorithm (Appendix 6), learn skills related to cultural sensitivity, and practice motivational interviewing skills. This training will be conducted by webinar and teleconference as well as in person during the 40-hour practicum that the AL Practice facilitators will spend in NC with the NC AHEC team. An important objective of the in-person time spent in NC is to establish relationships with mentors who serve as a resource and support throughout the study period. Once trained, facilitators participate in biweekly calls to troubleshoot and brainstorm ideas. Practice facilitators also reach out to their mentors for ongoing help and advice.

6.4. b Peer Coach Training

Peer coaches are trained using the programs we have developed in the course of our work in the Black Belt.[14,15] Unlike practice facilitation, there is no nationally recognized certification program for peer coaches. Training is made up of 8 courses that take place in-person and by phone or webinar. The goals of the training include: peers are confident to implement the program as designed, peers understand ethical obligations of being part of a research study team, and to establish an infrastructure for continued support and education for that clinic's peer group. Peer coaches are certified for each of the first 8 program sessions and the maintenance phase after each training course. Our training approach begins with 1 or 2 in-person workshops, which take place in community settings. We spend the day covering techniques of motivational interviewing and supportive counseling, with live modeling and ample opportunities for role-playing and practice. Coaches also learn how to use a BP home monitor. Certifications are scheduled to be completed over the phone after

each training course, with opportunities for later telephone remediation for those coaches who do not meet minimum standards of motivational interviewing and supportive counseling.

Training continues by telephone or webinar through a series of conference calls over 4 to 6 weeks. We provide basic information about HTN and its consequences, along with basic information on medications used to treat HTN along with common side effects and costs. We provide information on self-monitoring and the interpretation of BP readings. We familiarize the coaches with the same algorithm used in the practice facilitation intervention (Appendix 6), with instructions to reach out to the practice if they discover that the patient is not able to tolerate a new dose or newly added medication, or if the home BP is consistently above goal. The coaches learn common titration recommendations (e.g., double the dose of this medicine and then fill the new prescription at the pharmacy), allowing them to reinforce the plans with patients. Our discussions with practicing physicians during practice engagement have revealed that many patients have a great deal of difficulty with even the simplest medication titration instructions, and we will be sure that coaches understand how to follow these types of instructions.

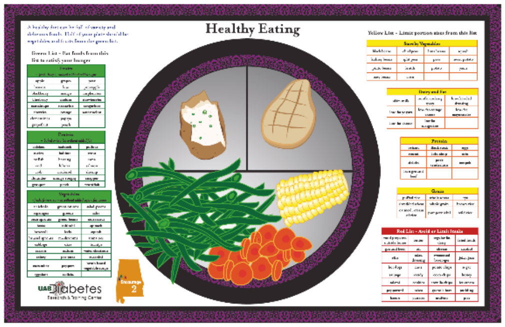

Figure 9. Placemat used in the peer coaching intervention

We also teach the peer coaches about healthy eating using the plate method (Figure 9) and emphasizing a few key messages in the form of easily remembered “sound bites” (e.g., “respect the border” to reinforce portion size control by leaving the border of the plate unobstructed by food; “be sweet on yourself” to avoid sugar sweetened drinks; “one and done” to avoid second helpings). This simple approach resulted in weight loss in the Encourage study even though weight loss was not a specific goal of that study. We add specific lessons on lowering salt intake, and serve a buffet lunch so that coaches can immediately put into practice what they learned. We also have a post-prandial exercise session to reinforce learning about physical activity. We finish the afternoon by providing an overview of the intervention, and practicing the first session using a buddy system and trainers that float between pairs, providing reinforcement of key skills. Coaches receive their manual and review the participant activity books and self-monitoring calendars. See Appendix 8 for a peer coaching training manual that is being adapted for the present study.

Since they are not healthcare professionals, peer coaches are often uncertain about their clients’ questions related to health, especially the numerous questions about medications. Therefore, coaches are trained on how to use the PALS, providing a resource for peer coach interactions with their clients designed to give them confidence in the reliability of the information they provide. As described above in section 6.3.b.i, the PALS is a web-based question-based system with evidence-based, referenced answers that covers basic facts about the pathophysiology of HTN, its long-term consequences, the medications used to treat HTN, and their cost and side effects. Answers are short, and when there is a need for didactics (e.g., “what is HTN?”), answers are in the form of engaging videos designed to encourage the viewer to watch the segment several times. The PALS will be made available to each practice on the laptop provided by the study, with the intent of providing factually correct education material for the practice’s patients (and staff). The PALS is also available wherever there is an internet connection, thus its use as a resource for the peer coaches provides the opportunity to encourage wider use outside the healthcare setting.

After the in-person trainings, coaches meet on weekly conference calls or by webinar to discuss the next chapter in the intervention, after which they practice the intervention at least twice, once playing the role of the patient and once of the coach. When they feel confident, they schedule a call with the research assistant, who plays the role of the patient, and the coach works through the session to become certified. Research assistants have the opportunity to reinforce skills over the course of this training program, which takes approximately 8-10 weeks to complete for an 8-session program and booster/maintenance sessions.

The peer coach training is timed to finish close to the start of intervention delivery. This timing resulted from requests from the peer coaches to maximize their confidence in their ability to deliver the intervention as

intended. Because of the duration of the study, peer coach training sessions will be held in both AL and NC in the later years of the study period in order to assure availability of peer coaches.

6.5 Duration of Intervention

The duration of the invention is one year.

6.6 Implementation of Intervention

6.6.a Staged Implementation across the 80 Participating Practices

We will implement the interventions across randomized practices in stages to maximize feasibility in this real-world setting. We will first implement the intervention at 4 practices, each randomized to one of the 4 arms of the study, which will constitute the vanguard. The vanguard will allow for minor modifications to study procedures based on the real-world challenges we will encounter, prior to wider implementation across all 80 practices. Following the vanguard, we will implement the study in stages of 10-12 practices at a time, as shown in Figure 11. This approach maximizes feasibility within the constraints of the available funds under this funding opportunity. We have designed the implementation to aim to complete data collection by the end of year 4, allowing sufficient time to complete data collection and data analysis in Year 5.

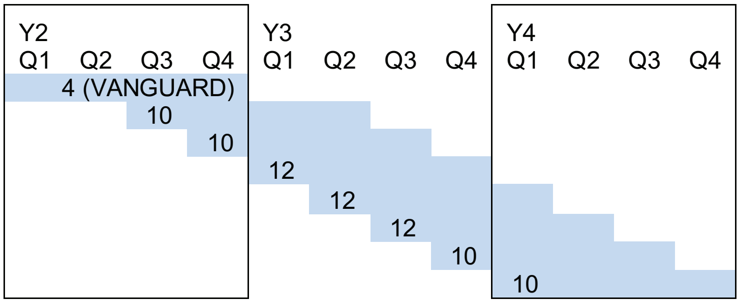

Figure 10. Staged implementation scheme for the 80 participating practices. Numbers indicate the number of practices per wave.

6.6.b Implementing the Interventions at the 80 Participating Practices

6.6.b.i Implementation of Enhanced Usual Care

At the time that patient recruitment starts at the practice, we will implement the enhanced usual care condition at all practices (see also section 6.3.a). Each practice will determine the best location for the laptop with the education system, along with a set of headphones for patient use. All practices will also receive a binder of practice tips, including the BP medication management algorithm (Appendix 6). All patient participants regardless of study arm will also receive a HPBM at the time of baseline data collection, and the research assistant will instruct each patient in its use.

6.6.b.ii Practice Facilitation Intervention Implementation

For practices randomized to receive the practice facilitation intervention arm, a practice facilitator will contact the practice within one week of the start of recruitment and initiate the practice facilitation intervention. The practice facilitator will meet with the practice face-to-face within 2 weeks of the initial call, and initiate collaborative planning with the practice team using the readiness assessment tool, called the Key Drive Implementation Scale (KDIS). This tool serves dual purposes with roles during recruitment as part of the assessment of the practice's ability to participate as well as during the intervention itself as a kind of barometer

of the progress that the practice is making. Practice facilitator will make face-to-face visits at least monthly for the duration of the intervention period. In addition, the practice facilitator is in frequent telephone contact with the Practice Champion in order to provide ongoing consultative advice on the practice's transformation efforts. At each monthly visit, practice facilitators will assess the practice's progress using the KDIS, and compare this assessment with the practice's self-assessment. This comparison is designed to stimulate conversation, action planning, and build rapport. Both the practice facilitator assessment and the practice's self-assessment serve as process measures for the intervention.

The BP titration algorithm is a central focus of practice facilitator activities (Appendix 6). If patients are adherent with medications and lowering salt intake, BP can be controlled in upward of 80% of African American patients. However, many may not achieve a BP <140/90 mm Hg despite following the algorithm. In these cases, Drs. Oparil (in Alabama) will be consulted for tailored advice on medication titration. Dr. Oparil is a leading clinical trialist in the field of HTN, having served on the 7th JNC and co-chairing the 8th JNC. She was also the PI of the SPRINT. In addition, she has led a HTN referral practice for over 30 years.

An important aspect of practice facilitation is the management of change fatigue. The practice facilitator Collaboratory is designed to provide peer support among practices undergoing transformation.[77-81] [75-79] The practice facilitator Collaboratory is a biweekly conference calls with a short presentation from one of the practice facilitators discussing how they overcame barriers in collaboration with the Practice Champion and/or team and succeeded at implementing the 4 key functions. Facilitators are partnered with experienced practice facilitator mentors from the NC AHEC, and they are encouraged to reach out to their mentors for help and advice. If needed, the study's physician leaders may be asked to communicate with the practice team to provide advice as academic detailers, drawing on their collective experience with practice change interventions. The calls are recorded for those that miss a call. Others bring their challenges to the group for brainstorming. In addition to the conference calls, a study specific list serve is being used by the practice facilitators; this messaging capability allows practice facilitation practices to share experiences and successful strategies for overcoming barriers. The Practice Facilitation lead investigator, Dr. Halladay, and the AHEC point person, Ms. Mackey, are available by cell phone for in-the-moment consultations. These support mechanisms serve as an important source of invigoration, overcoming change fatigue.

6.6.b.iii Peer Coaching Intervention Implementation

Practices assigned to the peer coaching intervention arm have a peer coach assigned to each recruited patient. The coaches meet the Practice Champion face-to-face at the start of the coaching intervention, and they firm up communication plans. Peer coaches contact the practice monthly to provide updates on progress using a structured form that may be placed in the patient's record at the practice's discretion. In between these monthly contacts, telephone contact is maintained with the Champion as needed to meet the patient's needs.

At enrollment, each participant receiving the coaching intervention receives a packet with their DVD and activity book. We had originally planned to provide study phones to participants but were unable to do so due to financial constraints. All participants will be briefed on the availability of so-called 'Obama phones' that are available free of charge to individuals with low incomes.

The peer coach contacts the participant within 2-4 weeks of enrollment, and sets up a time to conduct the first telephone session of the intervention. They instruct the participant to watch the first chapter of the educational DVD in preparation for their first intervention session. They work through each intervention session on the telephone, reviewing the content of the DVD as the springboard to their discussion. The coach encourages the participant to examine their barriers to achieving BP control, including barriers to medication adherence, healthy eating, physical activity, and keeping their appointments. They collaboratively set a realistic, achievable goal, which the participant tracks daily until their next session. The coach also encourages the participant to monitor their BP using the home monitor and to record their numbers to familiarize themselves with normal levels of BP and their own progress toward achieving control. The coach assists the patient in reaching out to the practice for problems such as inability to afford medications, side effects, or high readings that may trigger

escalation of medication doses. The PC start date is the date of consent and the intervention ends exactly 365 days later. For example if a participant provides informed consent on January 15, 2018, they end their PC intervention on January 14, 2019.

Once the initial 8-session program has been delivered, the monthly calls will be intensified to weekly calls for patients who have high home blood pressure levels as well as reaching out to practice champion. For patients who cannot be controlled despite appropriate titration of medications by the practice, Drs. Oparil and Viera will advise the practice on alternative approaches to achieving control on a case-by-case basis.

Once the BP is consistently in the controlled range (or as controlled as possible after guidance provided by Dr. Oparil), contacts become less frequent. Coaches are asked to contact clients at least monthly to assess progress on personal healthcare behavioral goals and to check on home BP monitoring numbers. Should BP become uncontrolled, contacts are intensified with reassessment of medication and dietary adherence and outreach to the practice for medication titration or medical management, including an additional office visit as deemed necessary by the doctor or nurse practitioner. The coach assists the client in making arrangements to keep any appointments at the practice. Weekly contacts are maintained until the BP is controlled or as controlled as possible, where after contacts are again made less frequently, at least once per month until the end of the intervention period.

7. Measurements

7.1 Overview of Measurements

The study will collect outcome measures based on physiologic data, patient-derived data in the form of surveys, medical record-derived data, and a variety of process measures designed to understand which aspects of the interventions were most successful in driving change. The potential change drives and their associated outcomes are shown in Table 13. Each of these data elements and the frequency and timing of their assessment is discussed in more detail in this section.

Table 13. Change drivers and driver outcomes and how they will be assessed. See also Figure 4 on page 12.

Intervention	Change Drivers (example activities)	Driver Outcomes
Peer Coaching	*Reinforce care plan: CM*	*Affordable medications: PS*
	Emotional, behavioral support: PS	*Side effects, adherence: PS*
	Self-monitoring support: CM, PS	*Healthier diet: PS*
	Overcome barriers: CM, PS	*More physical activity: PS*
	Link to care: CM, PS	*Stress: PS*
	Link to community: PS, CM, web use	
Practice Facilitation	*Registries: PrS*	*Outreach: PS, PrS*
	Audit and Feedback: PrS	*Timely medication titration: MR*
	Home BP monitors used: PrS, PS	*Case management: PS, PrS*
	Telephone management: PrS, PS	
	RN applies algorithm: PrS	
	Refer for nutrition: PrS, PS	

CM = coach manual. PS = patient survey. PrS = practice survey. MR = medical record review.

7.2 BP (Primary Outcome) and Height and Weight

Physiologic measures are assessed in the practices by a trained, quality controlled research assistant following standardized protocols guided by JNC recommendations. The protocol for these assessments is shown in Appendix 10.

To measure the BP, the research assistant first measures the upper arm to determine the appropriate cuff size. The participant is seated with both feet on the ground and asked to rest for 5 minutes prior to assessing the BP using an *Omron IntelliSense*™ digital BP machine. Two measures are taken and the second is used for analyses. Height and weight are assessed using a digital scale and a stadiometer. All research assistants use identical equipment for data collection.

BP, height, and weight will be assessed at baseline. BP and weight will also be assessed at the 6-month follow-up and the 12-month follow-up.

7.3 Patient survey

Quality of life and patient satisfaction are assessed as part of the study's secondary outcomes. These measures meet **PCORI Methodology Standard 2:PC-3** which recommends the use of patient-reported outcomes when patients or people at risk of a condition are the best source of information. Where available, we use validated instruments with acceptable psychometric properties in the targeted population (Appendix 10) The use of validated instruments meets **PCORI Methodology Standard 3:IR-4** (Document validated scales and tests **-** Studies should include documentation of the name of the scales and tests selected, reference(s), characteristics of the scale, and psychometric properties).[1] The instruments included in the patient survey are shown in Table 14. Two questions related to the gender and race of the patient's primary care provider were added to the survey.

Minimizing participant burden is a major emphasis in pragmatic trials. In this study, one of the interventions acts through relationships, which can only be assessed through participant report. Further, health behaviors are targeted, which are poorly documented in medical records. In our past work we have learned that community members value programs targeted at CVD risk reduction that include diet, exercise and stress reduction, thus they are included here.

Table 14. Domain, instruments used to assess the domain, and assessment schedule of the patient survey

Domain	Instrument	B	6m	12m
Demographics	Insurance	Y	Y	Y
	Education	Y	N	N
	Employment	Y	N	N
	Income	Y	N	N
	Marital status	Y	N	N
	Ethnicity	Y	N	N
	Provider Demographic	Y	N	Y
Satisfaction (outcome)	Patient Assessment of Care for Chronic Conditions (20)	Y	Y	Y
	Perceived Usefulness of CHW	N	Y	Y
Quality of life (outcome)	Short form 12 (12)	Y	Y	Y
	Patient Health Questionnaire (8)	Y	Y	Y
	4-item Perceived Stress Scale (4)	Y	Y	Y
	Questionnaire for verifying stroke-free status	Y	Y	Y
Social Support (outcome)	PROMIS Social Functioning – 3 subscales (12)	Y	Y	Y
Health literacy	CHEW Health Literacy Scale (3)	Y	N	N
Numeracy	3-item subjective numeracy scale (3)	Y	N	N
Knowledge (barrier)	Hypertension Knowledge and Attitudes (11)	Y	N	Y
Barriers	Barriers to Medication Adherence (20)	Y	Y	Y
Patient Activation (activation)	3-item Patient Activation Measure (3)	Y	Y	Y
Medication adherence	4-item Morisky Medication Adherence (4)	Y	Y	Y
Fruit & vegetable intake (behavior)	Pfp FV question (1)	Y	Y	Y
Physical activity	REGARDS / NHANES PA question (1)	Y	Y	Y
	Framingham Heart Study PA question (1)	Y	Y	Y
Tobacco use	NHIS Tobacco use questions (2)	Y	Y	Y
Alcohol Use	AUDIT-C (4)	Y	Y	Y
Goals and Attainment of Self-Define Goals	Patient goals and attainment of self-defined goals (15)	Y	Y	Y
Safety/side effects (safety)	Falls (1)	Y	Y	Y
	Side effects re: BP meds (10)	Y	Y	Y
	Utilization – Hospital	Y	Y	Y
	Utilization – ED or Urgent care	Y	Y	Y
	Utilization – Primary care doctor / NP	Y	Y	Y
	Question to assess if PC office called to discuss HBP	Y	Y	Y
Cognitive Function	6-item screener to identify cognitive impairment (6)	Y	Y	Y
Comorbidities	Diabetes questions (2)	Y	N	N

	Comorbidities questions (8)	Y	N	N
Discrimination / Trust	Discrimination in healthcare scale (11)	Y	N	N

We recognize that several of the proposed domains are not ideally assessed using brief self-reported measures, specifically diet, physical activity, and healthcare services utilization. However, given the pragmatic trial context and the main interest in BP, we use brief measures that have been widely implemented in well-established surveys, minimizing participant burden. Health services utilization has been shown to be reliably assessed through patient report when the interval being assessed is shorter than one year.[82]

If the interview cannot be completed in person, the interview is administered by a research assistant on the telephone shortly after the data collection visit in the practice. Research assistants are trained and certified for cultural sensitivity and interviews are recorded for quality control. The elements of the survey collected at baseline, 6-month follow-up, and 12-month follow-up are also shown in Table 14. The steps to completing the 6-month and 12-month interviews with enrolled patients are listed below:

7.3.a **Completion of 6-month and 12-month interviews with enrolled patients**

All enrolled patients will be contacted for follow-up interviews at approximately 6 and 12 months after their date of consent.

a. The ‘gold standard’ for all interviews is a ‘face-to face’ meeting where the patient’s BP and weight are assessed by the data collector.

b. In special circumstances, data collectors will be allowed to conduct a follow-up survey over the telephone, but the patient must meet a data collector and have their BP and weight assessed to receive the $40 incentive payment. These cases should be discussed with the program manager.
    i. A minimum of 15 calls should be made to patients in attempts to arrange interviews.
    ii. Additional postcards and texts can be sent, alerting the patient that the study team is trying to reach them.
    iii. Calls to the practice champion should be made to check for new contact info, and to strategize on how to reach these difficult patients.
    iv. If you reach patient: emphasize the $40 incentive, much shorter interview than baseline

c. Remind patients to bring in their BP machines, or their hand written logs for the study team to copy or keep. Always give the patient extra logs, even at 12 months for their continued use and sharing with their provider.

d. 12-month interviews have higher priority. Extra efforts should be made to complete 12-month data collection.

e. Due to COVID-19, we have continued to encounter barriers to in-person data collection. Due to the time sensitive nature of our data collection schedule, we sacrificed in-person visits to conduct follow-up visits via phone/Zoom to complete the surveys. We also obtained IRB approval to allow participants to assess their blood pressure at home at the time of the survey and we recorded those values, but these values will not be used in analyses

7.3.b Timing of interviews

The schedule for completing interviews is shown in Table 14A and Figure 11.

a. To complete a 6-month interview ***on time***, conduct the interviews within 30 days before or 30 days after the date 6 months after informed consent. After this window closes, researchers should shift their focus to prioritizing 12-month interviews.

b. To complete a 12-month interview *on time*, conduct the interviews within 30 days before or 60 days after the date 12 months after informed consent. Note that the 12-month interview is due 12 months from the consent date – not six months after the 6-month interview date.

c. Interviews can still be conducted up to 60 days after the official due date, but they will be considered *late*. Interviews that are past their time window should be prioritized to complete late 12-month interviews over completing 6-month interviews if resources are too scarce to permit completion of both.

**Table 14A. Interview Completion Timing***	
**Interview**	**Timing***
Baseline	Within 30 days after consent date
6-Month Follow-Up	Within 30 days before and 30 days after 6-month target date
12-Month Follow-Up	Within 30 days before and 60 days after the 12-Month target Date
*Target date for the interview is determined by consent date +6 months for 6-month follow-up or +12 months for 12-month follow-up. See Figure 11.	

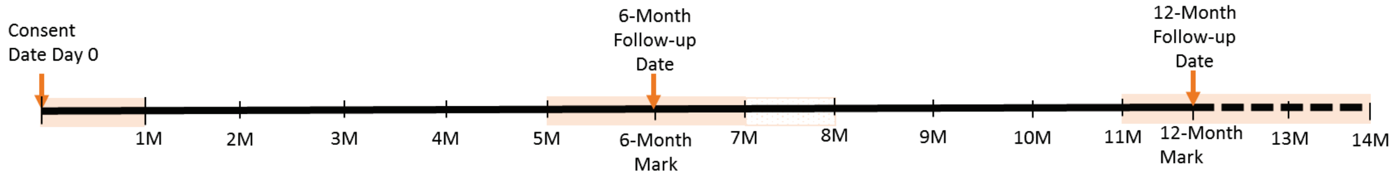

Figure 11. Ascertainment of windows for follow-up data collection.

For all unreachable patients, Program Managers should be notified. Program Managers will help strategize and change the patient status in *ClinvestiGator* to either "non-responsive," "lost to follow-up," or "dropped out"**.** The *ClinvestiGator* scheduling report automatically calculates interview due dates, and color codes them when they are 'do-able.'

For example, for a patient consented on January 1, the official 6-month mark is July 1 and the official 12-month mark is January 1 of the following year.

a. Six month 'ON TIME' can be completed as early as June 1 and as late as August 1.
b. Six month 'LATE' can be completed as late as September 1.
c. Twelve month 'ON TIME' can be completed as early as December 1 and as late as February 1.
d. Twelve month 'LATE' can be completed as late as March 1.

7.3.c Participant Follow-up Phone Call Attempts

Recognizing that there are constraints on available resources, this section provides guidance on the number of calls made to a participant to schedule a follow-up study visit. Up to 15 calling attempts will be made for each phone number in the sample. These attempts should be made at various times to increase the chances of reaching the participant. For example, 20% of surveys have been completed on weekdays and 80% on week nights and weekends. Changing the schedule to accommodate holidays and special events is an important strategy. Make weekday calls just after the dinner hour. Make appointment callbacks during hours that are not scheduled for other interviews, generally on weekdays.

7.3.d Text Messaging

Text messaging is available in *ClinvestiGator* for research assistants to assist with follow up scheduling and reminders, as well as retention messages. Text messages can be sent from the patient data base in *ClinvestiGator* with responses recorded within the database to track correspondences. The text messaging option is available to all sites under the guidance of the program managers. This is not a requirement but is available as an additional resource if desired. The two main purposes of text messaging are:

1. To remind study participants of their upcoming 6 or 12 month follow up appointments to reduce the number of “no show” for follow up visits. The approved verbiage is:
   “You have a UAB visit at [Practice name] tomorrow, [date], at [time]. Call xxxxxxxxx for questions” OR
   “You have a UAB visit today at [Practice name], at [time]. Call xxxxxxxxx for questions”.

2. To inform study participants in the peer coach arm to expect a phone call from their peer coach. Messages include the peer coach’s name, cell phone # (xxx)xxx-xxxx, and the date/time of the scheduled call. Participant are more likely to answer the peer coach’s initial contact, if they know the number in advance to avoid confusing the call with telemarketing. The intent of this text message is to improve participant compliance with the Peer Coaching intervention. The peer coach cell phones are UAB owned. The proposed verbiage is “[name], your peer coach, will call you on [date], at [time] from this #: xxx-xxx-xxxx.”

Text messaging will only apply to newly enrolled patients who have read the communications study page and gave permission to be contacted by text messages for the 2 reasons mentioned above. Moving forward, this communication page will be added to the consent form as an additional page.

Study staff will use the approved verbiage that is embedded in the system to send an alert message from the data system and not from an actual cell phone.

The day of consent:

1- Make copies of the communication page and add to your consent form
2- Help patient understand it and ask them to initial where indicated (whether yes or no)
3- Enter or update the patient cell phone number at the time of enrollment in the contact information section in *ClinvestiGator* and specify that this a mobile phone.
4- Once you do that, a box will appear next to the mobile phone # to include the carrier name (i.e, AT&T, Verizon, Sprint……etc.). Enter the carrier name (the carrier name is the most important piece of information). Without the carrier name, a text message can’t be sent.

How to send an alert text message: Go to the patient data base→ select your patient→ go to Reports → select “send text message via email” report→ confirm that your patient is selected in the box→ select approved verbiage→ update phone number and the peer name if indicated→ send.

7.4 Medical Record Derived Data

Medical records are abstracted for the following data at baseline and follow-up:

- Health insurance
- Medical conditions
    - Renal function (eGFR, creatinine, urinary albumin to creatinine ratio)
    - Chronic medical diagnoses that are part of the Charlson comorbidity index
- Medications (name, dose, frequency)
- Titration of antihypertensive medications in the past year
- Number of in-office encounters (including outreach and case management efforts by the practice)
- Number of hospitalizations with reason for hospitalization
- Emergency Department (ED) utilization, and telephone outreach
- For patients at practices randomized to the peer coaching arm: number of peer coaching chart entries

These data are abstracted using a structured abstraction form on the study’s web-based data entry system. Abstractors will be trained and certified and quality controlled to assure $\geq$95% reliability.[63,83-86] Medical record abstraction is conducted in the practice by the research assistant into laptop computers into the web-based study data collection system. In some cases, electronic records may be accessed remotely, in which case abstraction will take place remotely using an identical protocol as for on-site abstraction. Records will be abstracted at baseline, 6 months and 12 months.

Medical record abstraction will meet **PCORI Methodology Standard 3:IR-1**, which recommends assessing data sources for accuracy when selecting variables for confounding adjustment.[1] Alternatives are to ask participants directly, but the reliability of patients living in low socioeconomic circumstances for these types of data is not certain. Therefore, the most suitable data source available is the medical record.

7.5 Safety Measures

This study will not test the efficacy of a new BP medication, and the interventions are designed to implement guideline-concordant, evidence-based care that should represent the standard of care. However, although no novel treatments are being tested, we recognize that the efforts to lower BP to <140/90 mm Hg may result in some participants experiencing hypotension and resulting adverse events. We monitor adverse events through active surveillance at the time of data collection, asking participants whether they were hospitalized or had ED or office visits for falls or dizziness, and whether they experienced any symptoms that they attributed to BP medications. We also abstract this information from the medical records, including data on renal function, as shown in Table 15. All participants are provided a toll-free number to notify study staff of potential adverse events. Adverse events will be tracked in the study data system as described in section 8 and interim results will be monitored by the Data Safety Monitoring Board (DSMB).

An adverse event is defined as any untoward or unfavorable medical occurrence in a human subject, including any clinically significant abnormal sign (for example, abnormal laboratory finding), symptom, or disease, temporally associated with the subject's participation in the research, whether or not considered related to the subject's participation in the research. The burden of collecting and reporting data on every possible AE in this trial would require a great deal of effort, therefore all serious adverse events will be reported, and selected non-serious adverse events will be reported.

Consistent with NHLBI guidelines and OHRP policy, adverse events are events that meet any of the following criteria:

- Fatal
- Life-threatening (immediate risk of death from the event)
- Result in significant or persistent disability
- Requires hospitalization or prolongs hospitalization
- Important medical events that investigators judge to represent significant hazards or harm to research participants and may require medical or survival intervention to prevent one of the other outcomes listed in this definition (e.g., hospitalization, death, persistent disability)

Any event that meets any of these criteria will be documented and reported as a serious adverse event.

Table 15. Adverse Events and Source

Adverse Event	Source	Practice Facilitation (N =)	Peer Coaching (N =)	Both (N =)	Enhanced Usual Care (N =)	P value
Death	Chart review					
Ever had a stroke	Patient Survey/ Chart Review					
Ever had a myocardial infarction	Chart Review					
Ever have heart failure	Chart Review					
Dizziness on standing	Patient Survey (Falls/Side Effects)					
Fainting (syncope)	Patient Survey					
Falls	Patient Survey					
Falls precipitating medical treatment	Patient Survey					
Falls with head injury	Patient Survey					
Falls with fracture	Patient Survey					
Hospitalizations	Patient Survey/ Chart Review					
ED/urgent care visits	Patient Survey/ Chart Review					

Hypotension, SBP <90 or DBP <60 mmHg	Chart Review					
Bradycardia, heart rate <40 beats/min	Chart Review					
Hypertension, SBP ≥180 or DBP ≥110 mmHg	Chart Review					
Hyponatremia, serum Na < 130mEq/L	Chart Review					
Hypernatremia, serum Na >15 mEqL	Chart Review					
Hypokalemia, serum K < 3.0 mEq/L	Chart Review					
Hyperkalemia, serum K > 5.5 mEq/L	Chart Review					
Serum creatinine increase by at least 50% since previous measure	Chart Review					

Abbreviations: DBP = diastolic blood pressure; SBP = systolic blood pressure.

In reports to DSMB, all events will be reported, and a separate assessment will be made whether they are considered related or not related to the trial interventions. For DSMB reports, self-report of a stroke, heart failure, myocardial infarction, side effects, falls, hospitalizations, and ER visits will be combined with data from chart review.

All laboratory values found to be outside the safety range will be brought to the attention of the primary care office staff. Adverse events should only be documented in the 6 month and 12 month follow-up at the time that all 6 month and 12 month follow-up data are collected for a participant. The only exceptions are as follows:

- Participant dies
- Participant withdraws from the study and their reason for withdrawing was an adverse event (example, had a stroke or MI).

For these participants both a withdrawal and an adverse event form should be completed.

7.6 Process Data

7.6.a Process Measures for the Peer Coaching Intervention

The study has several sources of data that will be used to assess the peer coaching intervention dose and fidelity: cell phones, peer coach manuals, monitored interactions, and participant report through both spot checks and survey at final followup, as shown in Table 16. Each of these are described in greater detail in the following section.

Table 16. Measures of peer coaching intervention fidelity

Domain	How assessed
Intervention dose	*Frequency and duration of contacts (cell phone minutes)*
Degree of intervention implementation	*Peer coach intervention manual data, monitored calls, participant spot checks*
Emotional support	*Participant report at final follow-up*

7.6.a.i Cell Phone Data

Peer coaches will be provided cell phones to collect data on intervention dose. Cell phone data include the telephone number of ingoing and outgoing calls, as well as minutes per call. These data are used to total the minutes spent on the telephone for each session and the number of total telephone contacts between peer coach and client as a measure of intervention dose. In addition, minutes spent in contact with the practice by coaches is tracked, providing a measure of intervention fidelity related to frequency of contacts with the practice.

These data will be entered into the study's web-based data collection system.

7.6.a.ii Peer Coach Manual Derived Data

Peer coaches use a paper manual for each participant designed for multiple purposes:

1) A practical tool for coaches to document details about each client that is used over the course of the intervention (such as contact information, personal goals, etc.)
2) A tool to enhance intervention fidelity

3) A tool for process data collection

A copy of a chapter of the manual and client Activity Book are provided in Appendix 7. The coach has a separate manual for each client and records process data such as:

- Completion of each session
- Goal setting, including advancing the goal over time
- Adherence to self-monitoring, including home BP monitoring
- Medication adherence and barriers to medication adherence
- Adherence to low salt diet, physical activity, stress reduction
- Clinic outreach frequency and outcome
- Use of community resources
- Timing and frequency of booster contacts when BP becomes uncontrolled

Manuals are collected at the conclusion of the 1-year intervention period and data will be abstracted into a form on the study's web-based data collection system by trained and quality controlled research assistants.

7.6.a.iii Monitored Telephone Interactions

Intervention fidelity will be assessed by randomly selecting interactions for tape recording by the coaches. These sessions will be monitored by study staff with corrective actions taken as warranted to reinforce intervention implementation as planned, and to retrain coaches if necessary.

7.6.a.iv Participant Satisfaction: Spot Checks and Survey at Follow-up

Two strategies are implemented to assess intervention fidelity based on participant report. First, study personnel will make random telephone contacts with participants in the peer coaching intervention arms and assess whether all intervention sessions are being delivered in timely fashion, and also whether the coach is being supportive and helpful. Second, the final follow-up participant survey includes questions assessing satisfaction with interactions with the peer coach for those participants receiving the peer coaching intervention.

7.6.b Process Measures for the Practice Facilitation Intervention

The process data to be collected to assess intervention fidelity are shown in Table 17. There are measures of intervention dose as well as degree of intervention implementation, described next.

7.6.b.i Measures of Intervention Dose

Practice facilitators track frequency of contacts on forms that are part of the study's web-based data collection system. These forms serve the dual purpose of allowing the practice facilitator to track contacts across multiple practices in their caseload and to allow assessment of intervention dose. The total number and type (telephone brief and extended follow-up, in-person) of contacts is captured.

Table 17. Measures of practice facilitation intervention fidelity

Domain	How assessed
Intervention dose	*Frequency, type (in-person, face-to-face) and duration of practice facilitator contacts*
Degree of intervention implementation	*Monthly practice facilitator assessments of implementation of 4 key functions*

7.6.b.ii Measures of the Degree of Intervention Implementation

A practice assessment tool is used to assess intervention implementation monthly by the practice facilitator. The tool is filled out by the practice facilitator within the study's web-based data collection system. Separate sections assess the progress of the practice's transformation in relation to the 4 key functions of self-management support, use of the electronic record, team-based management, and outreach. We use a tool developed by Dr. Halladay called the Key Driver Implementation Scale (KDIS) which is adapted for the present study and focused on HTN.[37] The adaptation of this tool has been finalized through collaboration with the NC

AHECs and the AHRQ materials on practice facilitation. In brief, the KDIS assesses progress by the practice on leadership, team engagement, use of clinical information systems, standardized care processes, and self-management support (see Appendix 11). Sections on use of outreach and telephone management are being added for this project.

In addition, practice facilitators will track specific activities being implemented under each of the 4 key functions. Below, we provide some examples of the types of activities the practices may engage in. The facilitator will enter the specific activities into the study data system monthly, along with a qualitative assessment of the current stage of implementation (e.g., planning, early implementation, evaluation, etc.).

1) Self-management support
    - Implement a process for using home BP monitors for patients with uncontrolled HTN
    - Implement education on dietary salt restriction in a culturally concordant manner (i.e., use customized dietary advice that incorporates the kinds of foods local people eat)
    - Assess medication adherence systematically at every visit
    - Implement a process for supportively helping the patient to overcome barriers to adherence (such as finding the cheapest pharmacy, or switching to a more affordable regimen)

2) Use of the electronic record for population health management (Clinical Information Systems/Outreach)
    - Create a registry of HTN patients in the practice
    - Create a report identifying patients who are overdue for an encounter
    - Create a report of the practice's HTN control rate, by race and gender

3) Team-based management
    - Implement a protocol for BP titration carried out by a non-physician
    - Implement standing orders for BP titration, laboratory tests, recall appointments
    - Implement processes that permit non-physicians to provide self-management support
    - Implement systematic assessment of medication adherence and barriers to medication adherence completed by non-physician staff member

4) Standardized Care Processes
    - Work with practices to reach out to HTN patients who have not been seen recently
    - Work with practices to use the medication algorithm to guide medical decision making for their patients, specifically their AA population with uncontrolled HTN
    - Implement telephonic BP medication titration based on home BP measures
    - Implement protocol to use reports from the electronic record to recall patients who are overdue for visits

It is not expected that each practice implement all possible activities under each key function. However, it is expected that each practice attempt to implement at least one activity under each function over the one-year intervention period.

As discussed above, it is expected that it will take up to 3 months for the practice to implement its first activity. At the first monthly visit, the facilitator and practice staff will first get to know one another with ice breaker activities. At the next visit, the facilitator and staff members complete the practice self-assessment consisting of the KDIS (Appendix 11). They discuss the results and begin to learn basics of quality improvement including plan-do-study-act (PDSA) cycles. The first activity centers on using the practice's data to assess their current performance. The facilitator helps the practice to plan for how to obtain the data, whether from the electronic record or from a limited chart review of 30 patients with HTN recently seen at the practice. Many electronic record systems now include the ability to create registries and create reports such as on the practice's HTN control, but staff often have not had the time to learn how to create such reports. This is the role of the facilitator. The facilitator will teach the practice how to estimate their population-level BP control rate by using either the EHR or billing or scheduling data. Ideally, PDSA cycles would use 30-day denominators for estimating population-level BP control rates to assess the success of the QI intervention.

However, by using the model for improvement, the PF's work with the practices to use any data that is relevant to testing if their changes result in improvement, for instance if barriers to appropriate BP measurement are lessened over time, the PDSA data may simply reflect survey data from staff quantifying barriers monthly. Such barriers can include, but are not limited to, broken equipment, lack of right sized cuffs, physical environment not conductive to appropriate patient positions, and others. It may prove difficult in many practices to do this without some chart review due to challenges with customizing the data reports available in the EHR. Therefore, facilitators will teach practices to identify all patients with HTN (based on ICD-10 codes) seen in the past 30 days. If there are sufficient numbers (90 or more), a random sample of the 30 can be drawn by selecting a number n between 1-3, and then reviewing the record of every n^{th} patient to abstract only the last recorded BP. If there are not sufficient numbers (<90), the last 30 patients seen will be reviewed. The hope is that over time practices learn and see the value in higher quality data, thus will invest in processes that provide high quality population level BP control data. This is a key skill facilitators can build with practice staff.

Once the practice has examined its performance, the team is ready to plan its first improvement activity. The facilitator assists them in selecting their first improvement activity under one of the key functions, and the facilitator coaches them in setting an achievable, measurable, and realistic goal with detailed implementation plans for the first PDSA cycle. The first activity is carefully selected to be particularly achievable to let the practice achieve momentum. The facilitator documents these plans and then contacts the practice Champion weekly by telephone to track progress and help the practice overcome barriers they encounter. The facilitator visits the practice in-person monthly, with telephone contacts in between. All contacts are recorded and the facilitator completes the KDIS in the study's data system which includes a graph of BP control data over time that can be shared with the practice as part of their QI work.

As the practice makes progress, the facilitator assists the practice in selecting another improvement activity, encouraging them to eventually select at least one activity under each key function. Activities are evaluated as part of the PDSA cycles, and adjustments are made until the new process is deemed successful and ready for permanent implementation. The facilitator helps the practice to keep adding new activities under each function and documents progress for a total of 12 monthly visits and at least weekly calls between visits.

Practice facilitation will be assessed using questions that query general satisfaction with participation, workload, acceptance by the practice, difficulty in carrying out their expected duties, and support by fellow practice facilitators and mentors.

7.6.c Measures of Practice Satisfaction

Practices that receive either the practice facilitation or coaching interventions (or both) will be asked to complete an assessment of their satisfaction with the interventions. For practice facilitation, this will consist of the Practice Facilitation Provider Satisfaction Survey, recently used in a statewide program evaluation in Oklahoma (see Appendix 12).

7.6.d Measures of Peer Coach Satisfaction

Peer coach satifaction will be assessed using a set of questions used in several past studies that assess domains related to support from the coordinators, peer-to-peer mentoring, and ease of communication with the practices as well as how well the coach felt integrated into the practice team. See Appendix 16.

7.6.e Measures of Practice Facilitator Satisfaction

Practice facilitation will be assessed using questions that query general satisfaction with participation, workload, acceptance by the practice, difficulty in carrying out their expected duties, and support by fellow practice facilitators and mentors.

7.6.f Baseline Population-level BP Control Rates

Preliminary findings of practice-level BP control rates in hypertensive patients reveal that some practices have control rates <30%. FQHC's report higher rates, in some areas as high as 60%. Because practices with very low control rates may be more sensitive to intervention effects, we will attempt to estimate baseline population-level BP control rates in the practice's hypertensive patients for each practice separately. These values will not be used in the main analyses for the study since we will not be able to collect these data in a rigorous enough fashion to support more than exploratory analyses. Nevertheless, this will be a highly informative view of small private practice-level control rates in the heart of the Stroke Belt's rural areas, to our knowledge the first in the state of Alabama, as reflected in the Alabama State Department of Health's request to share these estimates as they accrue (advice offered at February 2017 Community Advisory Board meeting).

In order to obtain these rates, we will first request the past year's HTN control rate, which may or may not be readily available in EHRs. Ideally we will examine the practice-level control rate in the 365 days prior to the date of the first patient recruited at the practice. Preliminary experience indicates this may not be available at many practices. In this case, the practice's billing and scheduling databases will be queried for a list of all HTN patients seen in the 365 days prior to the date of the first patient's recruitment, using ICD-9 or ICD-10 codes. Duplicate visits will be removed, resulting in the denominator of patients with HTN, D. We will design the data system to select a random number n from the range (rounded to the whole number) {[D/30] ± .1*D}. Every n^{th} patient chart will be reviewed for the last BP recorded until 30 patients have been reviewed. The formula assures that the sample includes patients seen over the entire course of the year. The BP control rate will be calculated automatically in the study's data system.

We considered whether to limit these population-level control rates to just African American patients, concordant with the emphasis of this study. However, race data are so incompletely captured in these practices' data systems and medical records that this proved unfeasible. Therefore, population-level control rates will be estimated for this purpose on the practice's entire patient population with diagnosed HTN seen over the prior year.

7.6.g Collection of Home BP Data at 6-Month and 12-Month Follow-up Visits

All participants in the Southeastern Collaboration Study are asked to use a home BP monitor provided by the study free of charge, and are asked to record home BP in logs provided at enrollment, the 6-month, and the 12-month follow-up. While they are not to be used for the main analyses of the study, these data have multiple other uses to our study team, the practice facilitators and coaches, and to practice personnel.

The research assistants will program each new home BP monitor with the time and date before giving the monitor to a patient. This will facilitate accurate retrieval of data over the study period.

7.6.g.i To program the BP cuff:

1. Hold down the SET button (with the people and clock) until the year flashes at the bottom of the screen. Use the up and down arrows to select the year.
2. Push SET again to switch to date. Use the up and down arrows to select, hitting SET to switch between month and day.
3. Push SET again to switch to time. Use the up and down arrows to select the hour. Push SET to switch between hour and minute.
4. Hit SET a final time to complete setup.

7.6.g.ii Collecting BP data at six and twelve-month interviews

1. **REMIND**: Remind patients coming in for 6-month and 12-month follow-up visits to BRING WITH THEM their home BP monitor, and/or their home BP logs.
2. **Scan/Copy/Secure Fax:** If the patient brings in paper home BP logs, please obtain a copy. The method used will vary by implementation site and technology available. As always, treat patient information as confidential, and secure it in a HIPAA compliant manner.

3. **UPLOAD:** Once back at your university site, please upload the monitor data and/or scanned images into a computerized file. Please make sure that there is a patient ID associated with each set of home BP measurements.
4. **REPORT:** Share with the program manager that you have collected this home BP data and uploaded it. We will utilize work study students or others to enter this data into *ClinvestiGator* or other database.

7.7 Qualitative Assessments Post-Intervention

In addition to the quantitative individually completed assessments described in section 7.5 above, on conclusion of the intervention, we will collect qualitative data from all study participants – patients, coaches, practice facilitators, primary care practitioners, and practice staff to learn their perspectives on the interventions. We will develop discussion guides including participant perspectives on what went well and what did not go as well, in addition to their recommendations for long-term implementation and scale-up. These data will be collected via discussion groups as well as semi-structured interviews as detailed next (see also Appendix 15).

7.7.a Patients

We will conduct semi-structured individual interviews with patients in each of years 4 and 5 of the study, shortly after they have completed final follow-up. We will randomly select patients, assuring that we include men and individuals aged <60 years in the focus groups. For patients who participated in practices that received practice facilitation, we will include their qualitative impressions of the changes in the practice structure and how well they believe these changes improved their experience of care. For patients who worked with a peer coach, we will include their qualitative impressions of working with a peer coach, both positive and negative, and their opinions about working with a coach for a longer period of time, and how well telephone interactions met their needs and expectations (see Appendix 15).

Program managers at each site will work with their study team to determine which study team member will administer the interviews. Most will be administered at the time of the 12-month follow-up by the research assistant, but to reduce respondent burden, some may be administered within 30 days over the telephone. The program manager and study team at each site will select up to 5 participants at each practice for these interviews, aiming to achieve similar demographic composition as the study's overall sampling strategy to obtain insights from sufficient numbers of men and younger patients. Data will be entered into *ClinvestiGator*.

7.7.b Providers and Practice Staff

As with patients, we will survey the Practice Champion and provider after completion of the intervention period in ongoing fashion in years 4 and 5 of the study to obtain their perspectives on the interventions (see Appendix 15). The survey is designed for self-administration and includes open-ended questions. Only practices that received the intervention will be surveyed. Both provider and staff interviews are distributed via paper surveys for self-administration, and the data are entered into *ClinvestiGator* on receipt of the completed survey at the respective university.

7.7.b.i Post Intervention Survey Implementation

The survey should be fielded approximately 13 months after the first patient was consented, regardless of the recruitment period. In some cases, recruitment may last longer than the target 2-3 months. The timing of the Post Intervention Survey will be tied to the date of first consent, not the duration of recruitment.

There may be differences in how the survey is implemented according to intervention arm. In Practice Facilitation practices, the Practice Facilitator will notify the site's program manager via email after the last intervention session has occurred and provide the names and contact information of the individuals who were involved in the intervention activities that could provide relevant feedback on the intervention. The Practice Facilitator will recommend the most involved person, but in some practices, there may be more than one

individual identified. The number of surveys will be left to the discretion of the site PI and program manager, recognizing that the goal of these surveys is to obtain insights on the intervention implementation.

The knowledge of individuals at the practice by the study team is likely to differ at Peer Coaching practices. Because the main engagement in these practices will be with the Practice Champion and possibly the provider, it is likely that these two individuals will be the ones identified as those most likely to provide meaningful feedback. However, there may be exceptions where additional practice staff have been identified by study personnel as particularly engaged in the intervention. These names will be forwarded by the Peer Coaching intervention Workgroup to the study's program manager for survey administration.

The program manager will send out the survey to the identified personnel at each site and be responsible for collecting completed surveys within 30 days. While the surveys are designed for self-administration, if the practice personnel request a telephone interview to expedite completion, this will be left to the discretion of the program manager at each site. Timing of the surveys may differ by intervention arm. The intervention period for the Practice Facilitation intervention is from the date of the first recruited participant to 365 days after this date. The survey will be administered at any time after that date. The intervention period for the Peer Coaching intervention is determined by each individual participant's consent date, thus if recruitment is prolonged, the intervention period may be longer than 365 days after the first participant is enrolled at that practice. For these practices, the program manager will use the date of the last participant's enrollment to add 365 days to determine the end of the intervention period.

An additional Post intervention Semi-Structured Interview Brief Survey and Guide About Practice Facilitation was added. This will be complete among Practice Champions (see Appendix 12).

7.7.c Peer Coaches

We will have monthly meetings with peer coaches throughout the study period, actively seeking their feedback on implementation challenges in an ongoing fashion. We will conduct debriefing discussions with the peer coaches in year 5 of the study period to seek their advice about implementation and long-term sustainability.

7.7.d Practice Facilitators

We will interview each of the practice facilitators separately and as a group to learn their perspectives on the practice facilitation intervention and their opinion about its success, in addition to their perspectives on the special challenges that practices wishing to restructure to improve BP management may encounter. As for the peer coaches, we will teleconference monthly with the facilitators while the interventions are ongoing, obtaining ongoing feedback on the intervention. The final year discussions will therefore focus mainly on maintenance, sustainability and scale-up plans, as for the peer coaches.

7.7.e Maintenance Interviews with Practice Champions

An important part of the RE-AIM framework is maintenance, defined as how well the intervention can be sustained after conclusion of grant-funded support. Therefore, we will conduct semi-structured interviews with a sample of intervention Practice Champions one year after concluding the intervention period to assess which elements of either the practice facilitation or the peer coaching interventions were sustained over the year since they completed the interventions. These interviews will also serve to obtain further insights now that the intervention period is more remote. We anticipate that the practice facilitation intervention will result in lasting changes at the practice, therefore the sustainability of this intervention is likely to be high. We will ascertain whether practices moved on to NCQA recognition, and if so, which level they achieved. We will ask whether further improvements in their patient population's BP control have been observed in the interim since the last data collection was completed.

For the coaching intervention, we anticipate that ACA-related changes may have resulted in wider implementation of peer coaching in practices serving communities with a high prevalence of poverty, thus for

those practices that received the peer coaching intervention we will ascertain the degree to which the practice continues to work with peer coaches to improve the quality and outcomes of care.

7.8 Data Quality

Data quality is optimized through several strategies. First, we train and certify research assistants to follow protocols, most of which have been previously field tested. Second, we create checklists for data collection that guide data collectors through the protocol to assure adherence; these checklists are closely monitored with appropriate action taken for protocol deviations. Third, the web-based data entry systems have built-in range and quality checks, minimizing the entry of erroneous data. These systems also have robust reporting capabilities, permitting data collectors and their supervisors to track which participant is due for which type of follow-up to assure the timely collection of data. Fourth, we monitor the quality of data collected in the field through monitoring at the data coordinating center located at Weill Cornell, reaching out to field-based research assistants in near real-time to correct problems identified through monitoring (see also section 9). Last, we send two research assistants to the same site to conduct site quality checks (e.g., monitor protocol adherence) for 5% of the field data collection days. Quality issues are addressed through retraining and recertification as needed.

A major threat to data quality is low retention for the final follow-up, a problem that has challenged several studies in similar low income, minority settings, achieving 1-year retention in the 60-65% range.[87-89] We have achieved 85-95% retention in our studies using several strategies. Perhaps most effective is the use of trained community members to assist with outreach to participants. We ask for at least 3 contact numbers for family members and close friends for each participant on enrollment. The community-based study staff are highly supportive and assist participants to overcome transportation barriers in order to complete follow up visits.

8. Data Analysis

8.1 Overview of the Statistical Analysis of the Trial

In this section, we discuss the approach to preliminary analyses, the general analytic approach, the approach to testing the main hypothesis, additional practice-level and patient-level analyses, analyses to examine the heterogeneity of treatment effects, how we will handle missing data, sensitivity analyses, and power and sample size calculations. The analysis approach meets **PCORI Methodology Standard 3:IR-3,** which states that plans for data analysis should be specified a priori and should be designed to correspond to the major aims of the study.[1]

8.2 Preliminary Analyses and Data Summaries

All analyses will begin by examining numerical and graphical summaries of patient and practice characteristics, and outcomes (e.g., HTN control and BP at both the patient and practice levels). Smoothed bivariate plots will be used to examine unadjusted pairwise associations between variables and variability among stages of the trial and over time throughout the trial's duration. Particular attention will be paid to assessing whether distributions appear to be approximately normal within each study group and whether there appear to be any secular trends, conspicuously nonlinear relationships, or outlying observations. We will use analysis of variance (ANOVA) (potentially with rank-transformation if non-parametric tests are needed) and distribution-appropriate bivariate tests for pairwise and overall differences between and among study groups in baseline characteristics. Preliminary analyses will be shared with the DSMB as required by the sponsor.

8.3 General Analytic Approach

Strictly speaking, this is a cluster-randomized RCT factorial trial. However we believe that there is likely to be a negative interaction between the practice facilitation and the peer coaching interventions so that the effect of receiving both interventions will not be additive. For this reason, this trial can be better understood as a four-arm trial testing three interventions versus a single control group. While the traditional analysis of a factorial trial would assume the absence of an interaction (which it would likely be under-powered to detect) and proceed to test main effects, the presence of even a moderate interaction considerably reduces to power to detect main effects, even when the effect in the group receiving both interventions is larger than for either group receiving a single intervention. However, in this case there will be more than adequate power to detect the larger effect in the group receiving both interventions compared to the enhanced usual care control. The analysis plan and design have taken this into account to ensure adequate statistical power for bivariate tests of main effects between each the three intervention groups and the enhanced usual care group (control). All analyses will be intention-to-treat.

In this RCT, one of the interventions acts at the practice level (PF) and the other at the patient level (PC). Nevertheless, the outcome of interest is patient-level hypertension control. Therefore the main analysis will be at the individual patient level with appropriate modeling to account for the clustering of patients within practices. Recognizing that some experts recommend a practice-level analysis when the intervention operates at the practice level, secondary analyses will be done with practice-level data. The main hypothesis is that the proportion of patients with controlled hypertension will differ among study groups when analyzed without adjustment for covariates with the individual as the unit of analysis. Recognizing the stepped nature of the study implementation (see Figure 9), further analyses will also consider adjustment for baseline measures and secular trends. Patient-level analysis will have greater sample size, allowing for much more precise analyses and consideration of heterogeneity of effects among subgroups. For each patient-level analysis we will estimate the intra-class correlation (ICC) of patient-level measures within practices. If they are negligible (both statistically insignificant with point estimate <0.01), main patient-level hypothesis tests can proceed in the

same fashion as the practice-level analysis. If ICCs are non-negligible, unadjusted testing will use regression accounting for clustering with generalized estimating equations (GEE) or random effects but with adjustment for a limited number of baseline covariates such as age and baseline systolic blood pressure. All further patient-level analyses will use distribution-appropriate regression models (linear regression, generalized linear models, or generalized additive models) with additional covariates in addition to accounting for clustering within practices. Secondary parallel analyses will be conducted using the practices as the unit of analysis. Although likely to have less power, these analyses will not require accounting for clustering and will therefore have fewer assumptions, Unless otherwise specified, all statistical tests will be two-sided, and a p<0.05 will be considered to be significant. The analyses described in detail below are specific to changes in the rates of controlled HTN at the practice and patient levels. Analyses of all other endpoints will be carried out in parallel form, but with distribution-appropriate tests and regression models. All analyses will be intention-to-treat, regardless of intervention fidelity, intensity, and dose.

8.4 Testing the Main Hypotheses

By virtue of its factorial/multi-arm design, this study will provide evidence for the efficacy of three separate interventions, practice facilitation, peer coaching and an integrated hybrid intervention incorporating both compared the enhanced usual care control. The main analyses will consider the patient-level outcome of Stage 2 hypertension control (<140/90 mm Hg) at the 12-month follow-up.

There is thus a single primary hypothesis: that there will be statistically significant differences in rates of hypertension control among the study groups using logistic regression with adjustment for a limited number of baseline covariates such as age and baseline systolic blood pressure, and accounting for clustering using generalized estimating equations (GEE).

If the omnibus test is significant, we will proceed to identify which interactions had significant effects, testing three subsidiary hypotheses, one for each intervention:

1) that patients in practices randomized to practice facilitation have higher levels of hypertension control at one year than patients in practices randomized to enhanced usual care (EUC);
2) patients in practices randomized to peer coaching will have higher levels of hypertension control at one year than patients in practices randomized to EUC; and
3) that patients in practices randomized to both interventions will have higher levels of hypertension control at one year than patients in practices randomized to EUC.

Because patient-level Stage 2 hypertension control is a dichotomous outcome, the main test will be a two-sided chi-square test for differences in the proportion of hypertension control between study groups with p<0.05 serving as the threshold for statistical significance.

There is considerable controversy in the literature about whether the tests of each treatment group versus EUC require adjustment for multiple comparisons. To aid in interpreting findings, in addition to the standard p-values, we will also report p-values adjusted for the multiple comparisons using Dunnett's method.

8.5 Patient-Level Analyses

All additional unadjusted tests for differences between pairs of study groups will use the Benjamini-Hochburg approach to control the false-discovery rate at 0.05. These planned comparisons are1) practice facilitation vs. peer coaching, 2) practice facilitation alone vs. the hybrid intervention; and 3) peer coaching vs. the hybrid intervention.

Having performed the unadjusted analyses we will proceed through several more nuanced analyses accounting for particular characteristics of this trial. First, we will repeat the analyses above using linear

regression to estimate intervention effects adjusting for patients' baseline systolic blood pressure to reduce variance. We will then include terms representing secular trends, and time from baseline to follow-up of patients within each practice. Finally, we will use generalized additive models to explore whether any observed relationships are nonlinear in a statistically meaningful way.

Additional patient level analyses will allow us to use data observed at all time points as well as a much more granular analysis of moderating factors and differential efficacy among subgroups of patients. Two sets of patient-level analyses will proceed in parallel, one examining hypertension control as a dichotomous outcome (the primary outcome), and the other considering systolic BP as a continuous outcome. Patient-level analysis of hypertension control will use logistic regression accounting for clustering as described above. Initial analyses will proceed by examining smoothed longitudinal plots of study measures at each time point stratified by study group to characterize how intervention effects may evolve over time. Generalized additive mixed models will be used to model the outcomes over time to test for non-linear trends while accounting for the clustering of patients within groups and multiple observations per patient. If no evidence for nonlinear trends is seen, generalized linear models will be used in subsequent steps. Further analyses will include indicators for phase of the trial and calendar time as well as patient-level factors of explicit interest such as baseline SBP, practice type, age, literacy, depression, and gender.

8.6 Practice-Level Analyses

Modeling patient-level outcomes requires accounting for the cluster-randomization and findings may be somewhat sensitive to distributional assumptions. Although they will have less statistical power, we will perform parallel secondary analyses at the practice-level. Unadjusted analyses will use ANOVA considering the practice-level hypertension control rate as a continuous outcome. Modeling strategies will be similar to those described for the patient-level analyses adjusted for mean patient characteristics within practices. Because some of these analyses may use more degrees of freedom than is recommended for our number of practices, these will be treated as exploratory, and any findings will be carefully weighed against these limitations.

8.7 Heterogeneity of Treatment Effects

Although not the primary goal, an analysis of the heterogeneity of treatment effects is crucial for efficiently targeting interventions to patients for whom they are most likely to be successful. These analyses will be carried out at the patient level. Whether or not there is a significant difference between the group receiving the hybrid intervention vs. either alone, we will also be able to explore which of the three interventions is more effective for specific patient subgroups. The large number of patients will also allow us to examine this differential effectiveness within and between interventions by adjusting for predisposing factors (e.g. low literacy) and through analyses stratified by these factors. The explicitly planned subgroup analyses include stratification by gender, health literacy (as measured by the REALM), depressive symptoms (as assessed by the PHQ8), and age dichotomized as <60 vs. ≥ 60 years. An additional patient-level heterogeneity analysis will consider an interaction term between each patient's SBP and the intervention group.

We will formally conduct separate pre-specified heterogeneity of effects among practices by stratifying by FHQC status, practice size, and mean practice-level SBP of participants at baseline. These heterogeneity analyses will be done at the patient level with the formal test of heterogeneity determined by the interaction term between the practice-level stratification variable and the intervention. We will also examine associations with the variables collected in the practice characteristics questionnaire at baseline.

The planned analysis of the heterogeneity of treatment effects meets **PCORI Methodology Standards 5:HT-1,2,3, and 4**, which state that the goals of any heterogeneity of treatment effects analysis should be stated clearly (HT-1); the analysis plans should be pre-specified and any hypotheses related to heterogeneity should be pre-specified with supporting evidence provided (HT-2); all heterogeneity claims must be based on appropriate statistical contrasts among groups being compared, such as interaction tests or estimates of

differences in treatment effect (HT-3); and for any heterogeneity of treatment effect analysis, all pre-specified analyses should be reported as should, at a minimum, the number of post hoc analyses including all subgroups and outcomes analyzed (HT-4).[1]

8.8 Preplanned Subgroup Analyses

An important aspect of a pragmatic trial is the ability to examine the heterogeneity of treatment effects in high-risk subgroups of interest that are often difficult to recruit into research studies. We preplan subgroup analyses on:

1) Men,
2) Those younger than age 60 years,
3) Those with low health literacy/numeracy, and
4) Those reporting mild or greater depressive symptoms.

Past reports have shown that male sex and younger age are associated with higher risk for uncontrolled CVD risk factors, and health disparities are also greatest in younger individuals.[2,11] Low health literacy and numeracy and depression have also been shown to be associated with uncontrolled CVD risk factors.[48-52] We will therefore recruit at least 10 men and 12 individuals younger than 60 years of age at each practice; mild depressive symptoms are present in 50% of this population, and health literacy is also very low. The background prevalence and oversampling of men and younger individuals will provide enough power to conduct these pre-specified subgroup analyses.

These subgroup analyses meet the recommendations of **PCORI Methodology Standard 1:RQ-4**, which states that participant subgroups of interest should be identified and assessed.[1]

8.9 Sensitivity Analyses

We have planned other analyses to assess the robustness of our findings. The first set will include any outlying variables excluded during data cleaning. The next set will trim the upper and lower 5% tails of observations with continuous distributions to identify any associations that may be due to or exaggerated by extreme, but not spurious, observations. The final set of planned sensitivity analyses will consider process measures and measures of intervention fidelity to consider the association between variable exposure and outcomes. This will be particularly important both for planning future studies and for efficient resource allocation when planning for scale-up and wider implementation. Additional sensitivity analyses may be undertaken if missing data is a serious concern, as discussed below.

Sensitivity analyses are recommended in **PCORI Methodology Standards 3:IR-5** (which recommend the use of sensitivity analyses to determine the impact of key assumptions) and **4:MD-5** (which recommend that the sensitivity of inferences to missing data methods and assumptions should be examined and incorporated into the interpretation of the results).[1]

8.10 Sample Size Estimates

We plan to recruit 80 practices and 25 patients per practice for a total projected sample size of 2,000 patients. We estimate patient attrition at 20% (a conservative estimate given our past 85% retention in prior Black Belt studies), resulting in a reduced sample of 1,600. Because patients are sampled within practices, we accounted for clustering using a design factor $D=1+\rho(n-1)$, where ρ is the ICC and n is the number of patients per practice. The effective sample size (N_{eff}) is then N/D. In our Encourage trial the estimated ICC for systolic BP was 0.02, although it was not statistically significant. With 2,000 participants, N_{eff}=1351, or 337 per study group. We conservatively assume 20% practice-level attrition, resulting in dropping to 16 practices per group,

which decreases power more than reducing the patients per practice; the resulting N_{eff}=1081 with 270 per group. Given the readiness criteria required by practices in this study, we expect practice attrition to be much lower. We thus expect that the effective sample size estimate for 20% attrition with ICC=0.02 is highly conservative, and use it in the following power calculations to show what we expect to be our minimal detectable differences with 80% power.

8.11 Power and Significance Levels

All power calculations are based on two-sided tests with type one error rate of 0.05% and 80% power (Table 18). Practice-level analyses will generally rely on t-tests or Wilcoxon tests for differences between the change in HTN control between groups. Power calculations are given for pairwise tests assuming the 'worst case' scenario of a large interaction so that the effect in the group receiving both interventions is the same as the groups receiving a single intervention. For all scenarios presented, the power of the omnibus test is >80%. Given a less extreme interaction power increases rapidly both for the omnibus test and for the pairwise comparison of Given that only patients with uncontrolled HTN at baseline will be eligible, at follow-up we expect to observe that 5% to 35% will be controlled in the control group, and **at least** 15% more among the groups receiving an intervention, e.g. 20% to 50%. Patient-level analyses assumed a standard deviation of 16 mm Hg in individual-level systolic blood pressure measurements. For practice-level analyses, data from the Encourage trial conducted in the AL Black Belt suggests a standard deviation of 10 percentage points for cluster-level HTN control rates and a standard deviation of 16 percentage points was observed in a study of 60 primary care practices in North Carolina.[90] In this trial, because we will be starting with a more homogeneous sample of uncontrolled hypertensive patients we expect the standard deviation (SD) to be lower among the control group, and somewhat higher among the intervention group. Table 18 displays power calculations for individual-level analysis and for practice level analyses assuming standard deviations of 10% and 16% in practice-level rates for the control and intervention groups, respectively, and for SD of 8% and 10%, respectively. Because the observed SD among practices varies with the number of patients per practice, power calculations were based on simulation studies. To further guard against deviations from distributional assumptions, simulations also examined the power using Wilcoxon tests, which was found to be very similar.

Table 18. Detectable differences in the main study outcomes and examples of driver variables.

Measure	Enhanced Usual Care % or Mean (SD)	Detectable Alternative:			
		Patient Level		Practice Level	
		N_{eff}=1351 (337 per arm)	N_{eff}=1081 (270 per arm)	N=80 (20 per arm)	N=64 (16 per arm)
HTN Control Rate	5-20-35% (SD 10%)	11.2-29.7-45.9%	12.0-30.8-47.3%	18-35-50% (SD 16%)	20-37-52% (SD 16%)
	5-20-35% (SD 8%)			17-32-47% (SD 10%)	18-33-49% (SD 10%)
HTN Control Rate with Dunnett's adjustment	5-20-35% (SD 10%)			22-38-54% (SD 16%)	24-40-56% (SD 16%)
	5-20-35% (SD 8%)	11.7-30.5-47.1%	12.7-32.1-48.5%	18-34-50% (SD 10%)	20-36-52% (SD 10%)
Detectable Difference in SBP change (mmHg)	Individual-level sd of 16mm Hg	3.1mm	3.4mm	4.9 mm (SD 4)	5.5mm (SD 4)
Detectable Difference in SBP change: Dunnett's		3.9mm	4.4mm	5.5mm (SD 4)	6.2mm (SD 4)
MCS 12	53.6 (9.2)	55.6	55.8	62.0	63.0
PHQ8	6.6 (5.5)	7.8	7.9	11.6	12.2
PACIC	2.6 (1.0)	2.8	2.8	3.5	3.6
Patient Activation	27.9 (6.1)	29.2	29.4	33.5	34.1
HTN Tx Compliance	21.4 (5.1)	22.5	22.6	26.0	26.6

MCS 12 = mental health component summary score from the Short Form 12. PACIC = patient assessment of care for chronic conditions PHQ = patient health questionnaire. SBP = systolic blood pressure. SD = standard deviation. HTN compliance assessed via the Hill Bone scale. Tx= treatment. In the HTN control lines, we present three different hypertension control rates in the EUC arm, with corresponding detectable alternatives as shown for 1351 and 1081 patients, and also for. 80 practices and 64 practices For example, if the hypertension control rate in the EUC arm is 5% and the practice-level SD is 10% in the EUC arm and 16% 'in the intervention arms, with 80 practices, we will be able to detect control rates in the intervention arms of 18% or greater with 80% power with 80 practices and 20% or greater with 80% power with 64 practices.

Power for subgroup analyses: As indicated above, for patient-level analyses of pairwise differences between groups we can assume an effective sample size of at least 270 patients per group. For a patient-level analysis, only 80 patients per group are required to detect a difference of 5% vs. 20%. The implication is that subgroup analyses, even when limited to a third of the sample, will have sufficient power for the minimum anticipated main effect of a 15% difference. The further implication is that with a sample size 3 times larger than that required for testing main effects, the work of Brookes et al., suggests that we will likely have 80% power to detect interaction effects as small as 1.4 times the size of the minimum expected main effects.[91]

8.12 Procedures for Accounting for Missing, Unused, and Spurious Data

In a real-world study, some missing data is inevitable even with careful training, web-based data entry forms, and active quality controls. Our prior studies have taught us many effective approaches for minimizing loss to follow-up even among a disadvantaged and geographically dispersed population. In our Encourage trial we maintained over 85% follow-up over the course of nearly two years. In this trial, due to the readiness criteria required of participating practices, including data collection combined with routine visits in a known clinical environment with some data obtained by chart abstraction, we anticipate that follow-up will be even higher. Nevertheless if any data items are more than 15% missing we will analyze patterns of missingness and, if the data is plausibly missing at random, we will repeat analyses using multiple imputation via 10 chained equations to address the missing covariates. This approach results in retention of the sample, and results in less biased estimates. A common covariate with missing data is annual household income, which is often missing in 15-20% of the study sample. The complete case method would drop those without income information from the analysis, introducing biases. We have used multiple imputation with chained equations to retain the sample.

We will also pursue analyses of changes over time that will use data from all available time points, and thus make more complete use of the data where a participant's data are missing only at a few time points.

Unfortunately, in studies of this nature, missingness due to incomplete follow-up cannot often be treated as missing and is plausibly related to the study outcome. In these case where there is concern for imbalances in loss to follow-up among study groups, we will conduct sensitivity analyses assuming outcomes for those lost to follow-up that will assess the potential of their loss to have biased findings against the null hypotheses.

Our plans to address missing data meet the recommendations of **PCORI Methodology Standards 4:MD-1,2,3, and 5**. Standard 4:MD-1 recommends describing methods to prevent and monitor missing data. Standard 4:MD-2 recommends describing statistical methods that will be used to handle missing data. Standard 4:MD-3 recommends the use of validated methods to deal with missing data that properly account for statistical uncertainty due to missingness. Standard 4:MD-5 recommends examining the sensitivity of inferences to missing data methods and assumptions and incorporating this information into the interpretation of the study's results.[1]

8.13 Analyses to Assess Dose-Response

A particular interest in implementation settings is the effect close adherence to the intervention vs. less intensive implementation. To assess intervention dose in relation to effectiveness of blood pressure lowering, we plan several analyses to examine dose-response for each of the interventions.

For the peer coaching intervention, we will assess dose both quantitatively and qualitatively. We hypothesize that greater quantity and higher user-rated quality of interactions as well as self-efficacy for high blood pressure self-management will be associated with better outcomes. We will assess quantitative and qualitative dose through variables and data sources as shown in Table 19. We will test the dose-response hypothesis by examining the association between these variables and the outcome measures among those participants who received peer coaching. We will first examine the bivariate association between the dose

variables and each outcome measure separately, then we will examine the role of participant characteristics in this relationship by entering sociodemographic (age, gender, social support) and physiologic variables (duration of hypertension, baseline systolic blood pressure, number of antihypertensive medications used at baseline, presence of chronic kidney disease, presence of diabetes, use of insulin, number of comorbid medical conditions) into a multivariable model. We will observe the effect of entry of the covariates on the parameter estimate for the dose variable in each of these models. We will first examine each dose variable separately, then construct a model in which all dose variables are entered simultaneously, separately for each outcome. We will examine interactions with dose-response variables to explore whether subgroups are more or less sensitive to any observed dose-response effects, recognizing that the power for these analyses will be limited by the available sample size. We will also explore the feasibility of a dose-response score that incorporates all dose variables that are bivariately significantly associated with the outcome and weighting each variable according to the strength of the association, following the approach of Charlson.[92]

Table 19. Data for dose-response analysis of each intervention

Domain of Dose-Response	Peer Coaching (data source)	Practice Facilitation (data source)	Peer Coaching + Practice Facilitation (data source)
Quantitative	Number of intervention sessions delivered (peer coaching manual), mean number of minutes spent per session (cell phones), total time spent with the peer coach (cell phones)	Number of in-person contacts, mean time per in-person contact, total time spent in-person, number of telephone and email contacts between in-person contacts (data entry system)	As for Peer Coaching and Practice Facilitation; peer coach report of number of contacts with practice (peer coach manual); practice report of number of contacts with peer coach (6 and 12-month practice staff assessment); number of activities at practice that integrate peer coach (practice facilitator monthly entries into data system)
Qualitative	Satisfaction with peer coach, perceived supportiveness of peer coach, self-efficacy for high blood pressure self-management (12-month follow-up)	Key Driver Implementation Scale (KDIS) at 12 month follow-up (practice facilitators enter into data system); practice staff assessments of practice commitment to change, helpfulness of the practice facilitator (staff survey as 12 month follow-up).	As for Peer Coaching and Practice Facilitation; peer coach report of quality of interaction with practice staff (peer coaching manual); practice staff assessment of quality of interaction with peer coach (6 and 12-month practice staff assessment); intensity of integration of peer coach (practice facilitator monthly entries into data system)

For the practice facilitation intervention, we will assess intervention dose both quantitatively and qualitatively. We hypothesize that greater quantity of interactions, more intensive activity in each of the 4 Key Driver domains, and higher user-rated quality of interactions with the practice facilitator will be associated with better outcomes. We will assess quantitative and qualitative dose as shown in Table 19. We will analyze dose-response by examining the association between these variables and the outcome measures among those participants who received care at practices in the practice facilitation arm in analogous fashion to the analysis of dose-response for the peer coaching intervention. We will assess whether each of the quantitative and qualitative variables are associated with better outcomes first in bivariate analyses, followed by multivariable analyses examining the effect of participant characteristics on these associations, as well as tests of interaction. Analyses will also explore subdomains of quality of dose by examining the intensity of activity on each of the 4 Key Drivers assessed by the KDIS, first examining each Key Driver separately, then all 4 simultaneously. Members of our team have examined the KDIS as a simple ordinal scale and demonstrated associations of higher KDIS scores with better outcomes.[93] This analysis will explore whether intensive activity in one of the domains is more strongly associated with outcomes than intensive activity in another domain, for example, whether more intensive activity around self-management support is more effective than more intensive use of the practice's data systems. We will use a similar approach as outlined above for the

evaluation of a dose response scale for practice facilitation in the peer coaching intervention, following the approach of Charlson.[92]

For the hybrid practice facilitation with integrated peer coaching intervention, we will examine all the same variables as for each of these interventions separately, in addition to variables that assess the blended intervention specifically (see Table 19). The analysis will be analogous to that described for the peer coaching and practice facilitation interventions, adding the variables specific to the blended intervention.

9. Quality Control and Quality Assurance

9.1 Overview of Study Quality Control

Study quality control is a process that begins with the planning of the study and extends through the completion of data archiving. The implementation of this process will be under the direction of Dr. Joshua Richman at UAB and Dr. Hollenberg at Weill Cornell through the standing Data Work Group (DWG) that meets weekly to plan, implement, and review data reports to produce the highest quality data upon which to base study findings. The domains to be addressed by the DWG include focusing of study design and data variables, documentation, training, design of the data management systems, data entry monitoring, auditing of data entry, exercising of data, scheduled review of study progress, and data closure, described next. These approaches meet recommendations set forth in **PCORI Methodology Standard 3:IR-1** (assess the data source adequacy).[1]

9.2 Focusing of Study Design and Data Variables

Perhaps the most powerful factor in study data quality is a tight and strong study focus to collect only the required information in the most efficient manner. This focus has been a factor in the conceptualization of the Triple Threat study as a pragmatic trial, placing minimal burden on participants and practices.

9.3 Documentation

Having clearly written instructions for the trial is a vital component of data quality. The protocol will be the guide for the development of a much more dynamic Manual of Procedures (MOP) maintained by the DWG where decisions on the implementation of the protocol will be documented, thereby ensuring that the same approaches are applied uniformly. The MOP is viewed by the DWG committee as the guiding operational document of the study, and is subject to review and modification as the study progresses. The DWG will work with the field-based data collectors to ensure that current MOPs are maintained and that study procedures are followed rigorously.

These approaches meet recommendations set forth in **PCORI Methodology Standard 1:RQ-2**, which recommend developing a formal study protocol.[1]

9.4 Training

A shared understanding of both the protocol and MOP is critical to ensuring high quality data, and the key to this shared understanding is a strong and on-going training program. Each data collector will undergo training and certification in addition to an in person refresher training every 6 months as needed under the direction of each site's Program Manager, overseen by the DWG. The data collectors will meet weekly by teleconference and webinar for cross-training and the development and review of training materials. Training activities will include mock exercises, review of the biometrics measurement protocol, BP machine checks, and cuff size selection. New data collectors will be trained individually via phone and web-based training programs and in person. All training materials will be available on the trial web site managed at the DCC.

9.5 Design of Data Management Systems

One of the advantages of an integrated data system is extensive data checks that are performed at the time of data entry, which have been refined over 30 years of field use for the *ClinvestiGator* system. Range and validity checks (including cross-form checks) are performed as data are keyed, and the AL and NC program managers are informed immediately regarding data that do not meet the pre-defined validations on the program manager dashboard. The system allows for restricting data to specific options where violations are not permitted (for example, sex is restricted to either men or women, with no other acceptable options) and validating data that is entered, such as weight, where suspicious values are allowed with confirmation (for example, weights above 400 pounds are possible, but require confirmation by the data collector before adding to the database). In addition, data can be checked across CRFs to prevent conflicting information from being

entered and data from previously entered CRFs can be auto-populated in subsequent CRFs to prevent conflicting information from being entered.

9.6 Data Entry Monitoring

While data errors arising from data entry are a relatively minor source of errors in studies, tight data control will be instituted to ensure the highest quality data. Data entered will be assessed for patterns of unlikely options within a participant and practice, e.g., large weight fluctuations within a short period of time. If the DWG notice unacceptable error rates (i.e, consistently above 0.5%), we will undertake retraining of the field-based staff member.

9.7 Auditing of Data Collectors

Random audits of 10% of each field based staff with data entry activities will be performed to ensure that data entered into the computer system matches CRFs and retraining will be undertaken when appropriate.

9.8 Exercising of Data

Many data issues are identified when data analyses are performed. There will be an early and active program to produce draft DSMB reports and blinded reports similar to those that will ultimately be produced to interpret study results. Producing blinded reports early allows for time to ensure the correct programming to produce reports and provide documentation of approaches.

9.9 Scheduled Review of Study Progress

The DWG will be charged with weekly review of reports on recruitment, data completeness and data quality, and will monitor these reports at the level of individual practice sites. If recruitment does not meet the expected goal at each site within 30 days of recruitment initiation, site-specific plans will be developed.

9.10 Data Closure

As the study reaches completion, the DCC will actively work with data collectors and the practice sites to resolve any outstanding queries with the goal of data closure and documentation.

The DWG is one of the most important committees in the study organization. It is empowered to request additional information from any study unit (e.g., practice sites, facilitators, peer coaching committee, Executive Committee), to implement a stepped program to resolve discrepancies, where data collectors are first notified of issues, and then the relevant PI (Drs. Cherrington, Viera, Cummings) is involved to resolve issues with retraining or replacement of staff, as warranted. The DWG will provide a standing report to the Executive Committee at each of its meetings.

9.11 Audit Trails

The DWG will use well established approaches to create data audit trails for data and data entry (e.g., login attempts, selection of forms), requests for changes in the system, any changes to servers/software, system tests used in application validation and development, and physical security.

9.12 Data Security

The DWG is committed to ensuring the privacy and integrity of the data and systems under its authority. Best practices dictate adequate security measures are achieved in a multi-layer approach: physical, electronic, application, detection, and response to breech and training.

Physical security is maintained through video surveillance of the server room and hallways; two different systems of environmental surveillance; electronic locks on all doors providing access to servers; physical

securing of desktop PCs; onsite backup media are kept in a secure room and offsite backup media are kept in a fireproof safe in a secure room; and PCs and laptops are placed so that they are not publically viewable.

Electronic security is assured through PGP encryption; firewall Patchlink screensavers and installed antivirus packages; required login IDs and strong password authentication with passwords changed every 180 days; "least needed" access granted by the DWG; Event Sentry software to provide secure remote logging and notification of exceptional events.

Applications are designed following the Clark-Wilson model. User credentials alone will not possess sufficient rights to access study data via any other means than the application provided. Applications containing sensitive data will use Federal Information Processing Standard (140-2) compliant software to encrypt/decrypt the data. Passphrase used to initiate access to secured data is held in memory and not hard coded into the application.

Detection. Campus IT Security operates twice daily scans of every computer on the network for known security risks, such as open ports, missing software patches, weak administrative passwords, and misconfigurations. When a computer is identified, campus security notifies the responsible system administrator and sets a deadline for remediation. In egregious cases, the network port supporting the computer will be shut off. Campus IT Security operates network monitoring system which scans network packets for signatures of known malware, data streams addressed to known malware or suspect sources, or other anomalous (heuristic detection) network traffic. In the cases of known malware or known traffic to suspect sources, campus security will shut off the network port supporting the computer and notify the responsible system administrator. Anomalous network traffic is examined and may result in the shut off of the network port supporting the identified computer. Applications employing decryption mechanisms (as described earlier) will remotely log access attempts to data it manages; logging mechanism will have filters that can be set to send alert messages to responsible administrators if access attempts exceed guidelines and have the ability to completely halt access to secured data.

Security Response to Breech. It is institutional policy that all instances of data security breaches be reported to campus IT Security to initiate an investigation to document and determine the nature of the breech, what information is involved, how best to stop further loss, what remediation is necessary and if the breech is reported to authorities. Campus IT Security staffs an active response unit that provides forensics and maintains a close working relationship with State and Federal authorities.

Training. All DWG employees take part in annual training regarding data security. Application developers are required to read the "Application Development Guidelines SOP" and provide signed affirmation. Application developers are required to read and stay abreast of the guidelines provided by Open Web Application Security Project (www.owasp.org).

9.13 Data Handling and Recordkeeping

This study will utilize *ClinvestiGator,* under the direction of Dr. James Hollenberg, as its data management system (Appendix 13). *ClinvestiGator* will provide full service electronic data capture (EDC), data management, and reporting. Data management includes: database specification, development and testing; edit check programming; data management plan development and maintenance; ongoing manual data review and query management; and data cleaning and locking. *ClinvestiGato*r will generate monthly reports during the study to monitor enrollment and maintain and improve the quality of the study database and will facilitate the ability to:

(1) Collaborate and communicate with the study PIs to review the proposed protocol and contribute to finalizing that document;
(2) Develop case report forms;
(3) Develop relevant Standard Operating Procedures (SOPs);
(4) Develop and maintain full service EDC and data management that will include a web-based data entry system;
(5) Create and distribute data entry processes;
(6) Train and certify users in the data entry systems;

(7) Receive, collect, process, store, and analyze data collected from the participating clinical practices; and
(8) Participate in preparing and distributing quality control reports to the participating practices, study committees, and the sponsors and Research Coordination Unit.

9.14 Data Management

*ClinvestiGator h*as been successfully used to support over 40 studies involving over 2500 patients at multiple sites over the past decade. These studies have included externally funded projects with over $26 million in funding.

For studies with moderate or heavy activity (such as this proposed trial), a web-based data management system, where field-based data collectors are responsible for the entry of their own data, is both cost effective (as it removes a large proportion of the query process) and results in higher quality data and a reduced need for retraining. At the practice level, participant data will be entered directly into the web-based integrated system.

The integrity of the data is ensured by limited access and password protection. Each is assigned access to only the portions of the database they need. Access is provided under "hard" password protection (at least 8 characters and including both symbols and numbers) that are changed at 6 month intervals. All data are encrypted and data transfers into the system are one-way (data is not transferred out of the system). Incremental data back-ups are made on a daily basis, and complete data backups are made on a weekly basis and stored at an off-site repository. As a SQL database, interfaces for reporting with SAS are an ordinary feature of that package. Finally, the system being proposed is FDA audit ready, with appropriate Systems Life Cycle Documentation. The functionalities of the system are shown in Table 18.

Table 20. Functionalities of the data system that will support the trial.

Component	Comment
Establish eligibility	*Inclusion/exclusion criteria built into system.*
Manage participant flow in practices	*System updates data collection schedule at baseline, 6 and 12 months. Scheduling portion of system provides "real time" reports of expected assessments to be delivered for each participant. System presents participant-specific forms for each encounter, ensuring that the correct forms (and only the correct forms) are completed with each visit.*
Platform for data entry	*At each participant encounter, CRFs entered into laptops with secure internet access; data keyed into database at DCC. System tracks each CRF through its "life-cycle" from first data entry to completion and "locking" (i.e., all data fields completed, all pass data range and validity checks). System includes visual color-coded alerts for coordinator to instantly know of forms pending or overdue for specific participants. Once form is locked, data collector can review but not change data on form; data changes require DCC request with appropriate justification and approval prior to one-time use, participant- and form-specific, time-limited unlock code being issued.*
Data quality	*System enforces participant schedule with visits entered within windows; also monitors and tracks other components of data quality, including missed visits and missed forms and data lag for entry (i.e., overdue forms). In addition, system reports status of data quality to both local site (both summary and participant-level reports) and to the standing Data Work Group.*
Participant safety	*Tracking system for each AE. All CRFs associated with an AE event generate separate AE form for completion at practice site. When AE form entered, study safety monitor at UAB notified of AE through automatically generated e-mail. Safety monitor has access to details of AE through separate interface to system; as s/he evaluates AE (determine likelihood of association with BP drug, severity of AE, expected/unexpected nature of AE, resolution of AE), event can be closed and locked in system.*

AE = adverse event. CRF = case report form. DCC = data coordinating center.

The plans for data management meet **PCORI Methodology Standard 2:IR-1**, which state that data sources should be examined for accuracy to assure the robust capture of the needed covariates.[1]

10. Challenges and How we will Overcome them

Although we have an experienced team with established relationships in the communities we target, as well as highly engaged community members, pragmatic trials face several challenges. Recruitment of practices could lag, which is why we have planned for a staged process, with only half the total number of practices (n=40) ready to be randomized by the start of recruitment. While the emphasis on small practices engages the very practices that most need help, conducting the study in these practices will also be more difficult. We have therefore designed the study to allot no more than 10 practices per facilitator, a relatively low number across practice facilitation programs, but allowing facilitators to provide more intensive consultation if needed. Recruitment of African American patients is also notoriously difficult, especially for men and younger individuals. We have therefore minimized the number of patients per practice to 25. The team's past experience with similar interventions involving both practice facilitation (at UNC and ECU) and peer coaching (at UAB and Weill Cornell) permits the interventions to be refined using previously collaboratively developed intervention materials, speeding the intervention development timeline.

Once fielded, additional challenges arise. This implementation trial engages real-world interventionists, a strategy that enhances long-term sustainability at the potential expense of intervention fidelity. We will overcome this challenge by having engaged both facilitators and peer coaches in the intervention development process, which serves to empower them and make the study "their" study, enhancing internal motivation to adhere to the intervention protocol. We have developed multiple strategies to monitor intervention fidelity as detailed above in section 7.

Data quality in real-world settings can be compromised by nonadherence to high quality protocols for data collection. We therefore plan to have all study variables collected by trained research assistants rather than practice personnel. This is especially true for BP measurement, which has well documented departures from JNC recommendations in clinical care. Data collection will present additional challenges because patients may not be able to attend appointments at the practice. The community coordinators have been successful in assisting study participants to overcome barriers to attending study appointments in our past projects, and these same strategies will be employed in the present study.

Data systems for complex data entry and management needs for a study of this nature are challenging to create, especially in a relatively short timeframe. We use a well-established data collection system, *ClinvestiGator*, which has over 30 years of field experience. Data security is a challenge in all trials, but especially in trials conducted in real-world settings; the well-established approaches to data security outlined in section 9 that will be used by our DWG will lessen this potential threat. The depth of experience on the investigative team will be critical to the success of the trial, and the CABs, the DWG, and the sponsors all represent important resources to help and advise on how to overcome unforeseen challenges.

11. Ethical Considerations

11.1 Confidentiality

The confidentiality of all participant information must be protected at the Clinical Sites and within the data management system. Paper records and computer files must be appropriately safeguarded from unauthorized access. Paper and/or electronic records for study participants will be stored at the Clinical Sites. Copies of records identified by participant identification number pertaining to SAEs and study-defined clinical events, including necessary medical records, will be stored at the clinical data management system, *ClinvestiGator* and within the offices of the investigative team. These records will receive the same care as would ordinary medical records. They will be stored in locked filing cabinets and/or filing rooms within secure office space. Only study personnel who have completed STUDY training in data handling will have access to study forms.

11.2 Informed Consent

Before individuals may participate in any screening procedures, informed consent must be obtained. As described in section 5 above, informed consent will be obtained from all participants after a presentation by the trained study research assistants. Because low literacy is prevalent in the targeted region, the study will provide consent in a video format, an approach that will also standardize the consent process. The consent video will be integrated into *ClinvestiGator* and will include periodic comprehension assessments (mini quizzes) to assure that the content is being understood by participants. In the event a significant protocol change occurs, the informed consent will be adjusted appropriately and sites will submit the revised documents to their IRB for approval. Local IRB's will determine whether it is necessary to re-consent participants.

11.3 Institutional Regulatory Requirements

Annually, each participating institution will submit to the Principal Investigator stamped IRB approval letters and current copies of all consent forms. These records will be maintained within a central archive. Upon request, the consent forms may be released for internal IRB review. The study will be conducted in accordance with Good Clinical Practice (GCP), all applicable subject privacy requirements, and the guiding principles of Helsinki, including but not limited to:

1. Local Institute Review Board (IRB)/Central IRB review and approval of study protocol and any subsequent amendments.
2. Subject informed consent for the study. The study consent will contain the six essential elements from GCP guidelines that include:
    - Research statement, reasonably foreseeable risks or discomforts, reasonably expected benefits to subjects or others, appropriate alternatives, extent of confidentiality, compensation or treatment for injury.
    - Additional elements where appropriate such as unforeseeable risks to subjects, investigator-initiated termination of participation, additional costs, significant new findings, authorization for release of protected health Information for research purposes.
3. Investigator reporting requirements.

Written informed consent and Health Insurance Portability and Accountability Act (HIPAA) authorization must be obtained from each person prior to enrollment into the study. The study team will provide full details and template documents for the above procedures in the MOP and provide training to the investigators and clinical staff on regulatory and ethical considerations. All study personnel will be responsible for completing and remaining current with all applicable human subjects' protection, good clinical practice and data security and privacy training requirements

11.4 Ethical Considerations Related to this Patient Population and Peer Support Studies

There are a number of specific ethical issues that arise in relation to the study population and peer-support studies. African American residents in the Black Belt have deep felt, historically based distrust of the health care system. Peer coaching interventions have been utilized because of the strength of the trusting relationships that can be leveraged to foster health behavior change. Potential ethical challenges have been identified along with strategies for addressing them and are reproduced here for informational purposes.[94,95]

1. **Honouring the Dignity of Persons**
   Any relationship between persons must be premised on an understanding and acceptance that all people have an inherent dignity that has been variously codified in international documents. Peer support relationships must be founded on mutual respect.

2. **Selection and Training of Peer Supporters**
   This will be determined by the setting and may be through an open call for expressions of interest and/or an approach involving a person (e.g., a health care professional) who is acquainted with the potential peer supporter. Information governance principles need to be adhered to in this process. The selection process for those with, or without, given characteristics needs to be transparent, justifiable and fair. Peer supporters need to be trained in confidentiality. Peers have the right to confidentially refuse a given peer supporter: this may be more or less common with friends, relatives or neighbors.

3. **Professional-Lay Boundaries**
   Standard professions have delineations for boundaries within which relationships may be ethically practiced. While peer supporters are not professionals, support relationships are breeched when there are conflicting roles that compete with the primary goals of peer support. This means that peer supporters need to carefully negotiate the kinds of contacts and activities they enter into with their peers and have appropriate guidelines for support from clinical personnel.

4. **Simplified Informed Consent**
   The need for full informed consent for peer support interventions is a matter of some debate. Some have proposed that there are two aspects where implied consent may constitute an appropriate standard: intervention and the surrounding research. Agreeing to pair up with a peer or attend a group within an IRB approved framework should imply consent. Similarly, there are research activities where an individual gives consent by implication from his/her actions (e.g., completing an IRB approved questionnaire). However, more intrusive research activities (e.g., measurements, recorded activities, blood sampling) should require standard consent processes and these will be followed here. Medical record review needs to follow standard information governance procedures.

5. **Documentation of Peer Relationship and its Activities**
   Formalizing peer relationships requires establishing some basic standards of documentation of the peer relationship and of the activities and outcomes of such a relationship. While documentation in standard professions is elaborate, standards and scope of peer documentation are not well-defined. Certain critical cross cutting issues like ensuring completeness and accuracy, confidentiality, avoiding falsifying of records, and truth telling must be adhered to.

6. **Confidentiality and Privacy of Person's Records and Information.**
   The freedom to be left alone should extend to all peer support relationships. Patients do not have to document a written release in order to voluntarily share their own personal health information with a peer supporter. They can assume that it will be treated confidentially. They can freely choose whether to have a peer supporter and whether or not to share any personal health information.

7. **Involvement in Illegal Activities**
   Parties involved in peer relationships should not abet or foster crime including involvement with illegal drugs. Helping a peer partner in crime and covering it up is immoral and should be discouraged.

8. **Non-licensure to Practice Medicine**
   Peer supporters are not qualified nor licensed to diagnose, give medical advice or recommend medications. Their interventions involve support that in many aspects aids the implementation of the licensed medical practitioner's recommendations.

9. **Payment/Volunteerism**
   Any payments made to peer supporters must be carefully considered. A tension exists between the benevolence of volunteers and the capacity for health systems to exploit this benevolence which might undermine evidence-based (but more costly) structures and system changes. If a health system finances peer supporters, then there is an obligation to provide adequate training and support for their work.

10. **Support for Peer Supporters**
    Peer support volunteers must receive emotional support and adequate supervision in order to address potential emotional issues and other practical concerns that arise from their activities in working with patients.

12. Publication Policy

The purpose of the policy is to encourage and facilitate the presentation and publication of the study background, rationale, design, and analyses; ensure appropriate use of the study data, timely completion of manuscripts and presentations, equitable access to authorship, and adherence to established principles of authorship; and coordinate the reporting of trial results. The policy applies to all investigators analyzing, presenting, and publishing data from the study. There are several principles underlying this policy:

1. Research questions and hypotheses to be addressed using study data should be formulated a priori and clearly stated in a manuscript proposal to reduce the likelihood that study results are attributable to type I error.

2. Publication of scientific findings from the study should proceed in a timely fashion once relevant analyses are complete.

3. The publications arising from the study should avoid overlap and conflicting representation of study findings. Overlaps are, however, acceptable for review articles.

4. Recognition through authorship will be distributed among the study investigators so that all study investigators and team members have equitable opportunity to lead and co-author study publications.

5. The study should promote the career development of trainees and junior faculty by providing them the opportunity to lead and be recognized as co-authors of study publications, as appropriate.

6. Standards for authorship on study publications will adhere to the Uniform Requirements for Manuscripts Submitted to Biomedical Journals of the International Committee of Medical Journal Editors (NEJM 1997;336:309-315) and those established by the destination journals.

7. The concept, in the form of a proposal, for all manuscripts must be approved by the P&P Subcommittee prior to preparation. There are three categories of manuscripts and anticipated authorship:

    i) Main results developed based on core study data and study aims/hypotheses (which will bear the corporate authorship). The design and main baseline papers will also be corporate authored.

    ii) Manuscripts developed and authored by investigators using data that are not considered to be main study results.

13. Study organization

The study is a collaborative effort spanning four universities and several community-based organizations. This team is organized into an Executive Committee composed of the study investigators and lead staff, supported by work groups and the CAB, as shown in the organizational chart (Figure 11).

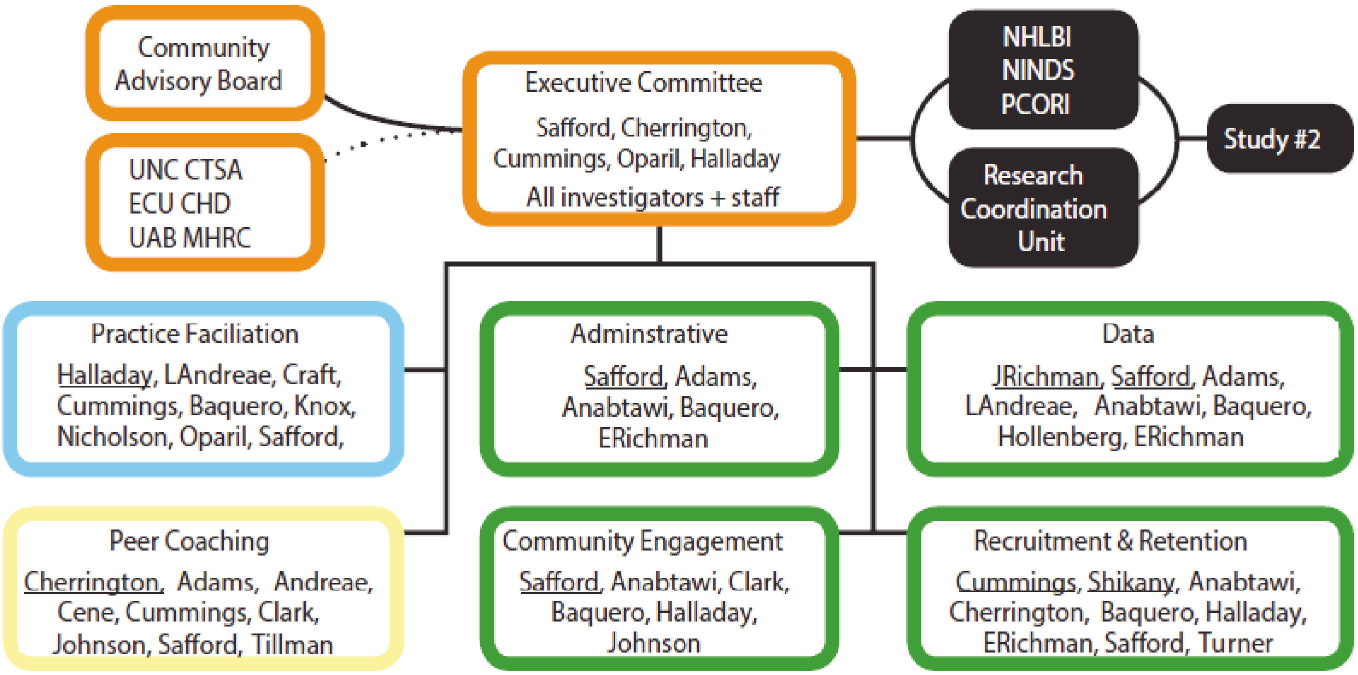

Figure 11. Organizational chart for the study. The Executive Committee is supported by practice facilitation, peer coaching, administrative, data, recruitment and retention, and community engagement work groups. Leads of each work group are underlined. The EC is also supported by the Community Advisory Board and university centers. The proposed study will collaborate with the sponsors, Research Coordination Unit and the other funded study. *CTSA = Center and Translational Science Award. CHD = Center for Health Disparities. MHRC = Minority Health Research Center.*

The study is organized as a multiple PI study. Dr. Safford is the overall scientific lead of the study as the submitting PI; Dr. Cherrington is the co-PI and contact PI in the multiple PI arrangement, since UAB is the recipient organization.

The internal **Steering Committee** meets monthly to obtain input from the group on major study decisions. Most of the work is conducted through the Workgroups. The Steering Committee is chaired by Dr. Safford, and co-chaired by Dr. Cherrington. Drs. Safford, Cherrington, and Andreae set the agenda for the Steering Committee. All investigators and the sponsors are invited to the monthly internal Steering Committee meetings. Once the study has begun to collect data, the internal Steering Committee will serve as the Publications and Presentations committee, approving study manuscript proposals, assuring appropriate authorship, and reviewing manuscripts prior to submission.

The Work Groups are designed to include investigators and staff from each of the four participating universities, and are each led by experts in the field. Work Groups meet every two weeks, and create time delimited ad hoc Work Groups as needed. For example, an ad hoc work group led by Dr. Oparil led the development of the BP titration algorithm for the study, which is integrated into both interventions (Appendix 6).

The **Recruitment and Retention Work Group** has been led by Drs. Harrington and Viera, but is now led by Drs. Shikany and Cummings, with Dr. Harrington's retirement and Dr. Viera's transition to a new institution. This group is responsible for developing the recruitment plan, recruiting practices, deciding on whether a practice is ready to be randomized, and conducting retention activities. This work group also oversees patient recruitment.

The **Practice Facilitation Work Group** is led by Dr. Halladay. This work group is charged with finalizing the practice facilitation intervention, overseeing the training of the practice facilitators, and overseeing the ongoing support of the facilitators. It will be responsible for monitoring intervention fidelity and taking corrective action should facilitators demonstrate lack of ability to implement the intervention as designed. The web-based data system permits reporting in near real-time, assuring the ability to closely monitor progress.

The **Peer Coaching Work Group** is led by Dr. Cherrington. This work group is charged with finalizing the peer coaching intervention, assisting with peer coach recruitment, overseeing the training and certification of the peer coaches, and monitoring intervention implementation as well as peer coach coordination and support.

The **Community Engagement Work Group** is led by Dr. Safford. This work group is charged with engaging stakeholders to obtain input on the design of the study, its implementation, and plans for scaling up. This work group coordinates community advisory board meetings, and in addition obtains input from practice facilitators, peer coaches, and practicing primary care physicians.

The **Data Coordination Center** is led by Drs. Richman and Safford. It is charged with establishing and maintaining the study's data systems and in-servicing study staff in their use. It is charged with providing randomization assignment for each practice, and assuring balance across practice types during the enrollment phase. It is charged with maintaining the integrity of the data, as well as providing interim reports to the DSMB. The Data Coordination Center will analyze the study's data for scientific manuscripts as well as reports for a wider lay audience.

The **Administrative Work Group** is led by Dr. Safford and includes study program managers from each university as well as the leads of the community-based organizations. It is charged with providing meeting support, assuring IRB approvals are obtained in timely fashion, creating and updating the study's protocol and manual of operations, creating reports as required by the sponsor, and managing the budgets and subcontracts.

References

1. PCORI Methodology Standards | PCORI. 2016; http://www.pcori.org/research-results/research-methodology/pcori-methodology-standards.
2. Mozaffarian D, Benjamin EJ, Go AS, et al. Heart Disease and Stroke Statistics-2015 Update: A Report From the American Heart Association. *Circulation.* 2014.
3. Howard VJ, Cushman M, Pulley L, et al. The reasons for geographic and racial differences in stroke study: objectives and design. *Neuroepidemiology.* 2005;25(3):135-143.
4. Washington BT. *Up From Slavery.* New York: Airmont Publishing Co; 1967.
5. Safford MM, Brown TM, Muntner PM, et al. Association of race and sex with risk of incident acute coronary heart disease events. *JAMA.* 2012;308(17):1768-1774.
6. Howard VJ, Kleindorfer DO, Judd SE, et al. Disparities in stroke incidence contributing to disparities in stroke mortality. *Ann Neurol.* 2011;69(4):619-627.
7. Howard G, Labarthe DR, Hu J, Yoon S, Howard VJ. Regional differences in African Americans' high risk for stroke: the remarkable burden of stroke for Southern African Americans. *Ann Epidemiol.* 2007;17(9):689-696.
8. Howard G, Prineas R, Moy C, et al. Racial and geographic differences in awareness, treatment, and control of hypertension: the REasons for Geographic And Racial Differences in Stroke study. *Stroke.* 2006;37(5):1171-1178.
9. Centers for Disease C, Prevention. Vital signs: awareness and treatment of uncontrolled hypertension among adults--United States, 2003-2010. *MMWR Morb Mortal Wkly Rep.* 2012;61:703-709.
10. Hansen J, Crowder C. Bleak diagnosis for sickly region. *The Birmingham News* November 17, 2002.
11. CDC. *CDC Health Disparities and Inequalities Report - United States, 2013.* Atlanta, GA November 22 2013.
12. Primary Care Physicians by Field. *State Health Facts* 2014; http://kff.org/other/state-indicator/primary-care-physicians-by-field/. Accessed December 1, 2014.
13. USCensus. American Fact Finder. http://factfinder.census.gov/faces/nav/jsf/pages/guided_search.xhtml. Accessed January 26, 2015.
14. Cherrington A, Martin MY, Hayes M, et al. Intervention mapping as a guide for the development of a diabetes peer support intervention in rural Alabama. *Preventing chronic disease.* 2012;9:E36.
15. Andreae SJ, Halanych JH, Cherrington A, Safford MM. Recruitment of a rural, southern, predominantly African-American population into a diabetes self-management trial. *Contemp Clin Trials.* 2012;33(3):499-506.
16. Herbert MS, Varley AL, Andreae SJ, Goodin BR, Bradley LA, Safford MM. Association of pain with HbA1c in a predominantly black population of community-dwelling adults with diabetes: a cross-sectional analysis. *Diabet Med.* 2013;30(12):1466-1471.
17. Sewell K, Andreae S, Luke E, Safford MM. Perceptions of and barriers to use of generic medications in a rural African American population, Alabama, 2011. *Preventing chronic disease.* 2012;9:E142.
18. NCQA. The Future of Patient-Centered Medical Homes. Foundation for a Better Health Care System. 2014; http://www.ncqa.org/Portals/0/Public%20Policy/2014%20Comment%20Letters/The_Future_of_PCMH.pdf. Accessed January 5, 2015.
19. Nagykaldi Z, Mold JW, Aspy CB. Practice facilitators: a review of the literature. *Family medicine.* 2005;37(8):581-588.
20. Frijling B, Hulscher ME, van Leest LA, et al. Multifaceted support to improve preventive cardiovascular care: a nationwide, controlled trial in general practice. *The British journal of general practice : the journal of the Royal College of General Practitioners.* 2003;53(497):934-941.
21. Baskerville NB, Liddy C, Hogg W. Systematic review and meta-analysis of practice facilitation within primary care settings. *Annals of family medicine.* 2012;10(1):63-74.
22. Nutting PA, Miller WL, Crabtree BF, Jaen CR, Stewart EE, Stange KC. Initial lessons from the first national demonstration project on practice transformation to a patient-centered medical home. *Ann Fam Med.* 2009;7(3):254-260.
23. HHS.gov. Better, Smarter, Healthier: In historic announcement, HHS sets clear goals and timeline for shifting Medicare reimbursements from volume to value. In: Services HH, ed2015.
24. Knox L, Branch C. Practice Facilitation Handbook. Training Modules for New Facilitators and Their Trainers. Rockville, MD: Agency for Healthcare Quality and Research; 2013.

25. Rogers E. *Diffusion of Innovations.* 4th ed. New York: The Free Press; 1995.
26. Jackson GL, Powers BJ, Chatterjee R, et al. Improving patient care. The patient centered medical home. A Systematic Review. *Ann Intern Med.* 2013;158(3):169-178.
27. Friedberg MW, Schneider EC, Rosenthal MB, Volpp KG, Werner RM. Association between participation in a multipayer medical home intervention and changes in quality, utilization, and costs of care. *JAMA.* 2014;311(8):815-825.
28. Rosenthal MB, Friedberg MW, Singer SJ, Eastman D, Li Z, Schneider EC. Effect of a multipayer patient-centered medical home on health care utilization and quality: the Rhode Island chronic care sustainability initiative pilot program. *JAMA Intern Med.* 2013;173(20):1907-1913.
29. Fifield J, Forrest DD, Burleson JA, Martin-Peele M, Gillespie W. Quality and efficiency in small practices transitioning to patient centered medical homes: a randomized trial. *J Gen Intern Med.* 2013;28(6):778-786.
30. Calman NS, Hauser D, Weiss L, et al. Becoming a patient-centered medical home: a 9-year transition for a network of Federally Qualified Health Centers. *Ann Fam Med.* 2013;11 Suppl 1:S68-73.
31. Leykum LK, Lanham HJ, Pugh JA, et al. Manifestations and implications of uncertainty for improving healthcare systems: an analysis of observational and interventional studies grounded in complexity science. *Implement Sci.* 2014;9(1):165.
32. Wiltshire JC, Person SD, Allison J. Exploring differences in trust in doctors among African American men and women. *J Natl Med Assoc.* 2011;103(9-10):845-851.
33. Adams M, Clay P, Ferguson J, et al. *Report of the Tuskegee Syphilis Study Legacy Committee - May 1996.* Charlottesville, VA: University of Virginia;1996.
34. Brownstein JN, Chowdhury FM, Norris SL, et al. Effectiveness of community health workers in the care of people with hypertension. *Am J Prev Med.* 2007;32(5):435-447.
35. CDC. Addressing Chronic Disease through Community Health Workers: A Policy and Systems-Level Approach. In: National Center for Chronic Disease Prevention and Health Promotion DoHDaSP, ed. Atlanta, GA: CDC; 2011.
36. Chen Z, Andreae S, Lewis M, Sewell K, Safford MM. Prevalence of beliefs about generic medications in Encourage study participants reporting medication non-adherence. University of Alabama at Birmingham Diabetes Day; May 1, 2012; Birmingham, AL.
37. Halladay JR, Vu M, Ripley-Moffitt C, Gupta SK, O'Meara C, Goldstein AO. Patient perspectives on tobacco use treatment in primary care. *Preventing chronic disease.* 2015;12:E14.
38. High Blood Pressure Facts | cdc.gov. 2016; http://www.cdc.gov/bloodpressure/facts.htm.
39. Ogedegbe G, Tobin JN, Fernandez S, et al. Counseling African Americans to Control Hypertension: cluster-randomized clinical trial main effects. *Circulation.* 2014;129(20):2044-2051.
40. Ogedegbe G, Tobin JN, Fernandez S, et al. Counseling African Americans to Control Hypertension (CAATCH) trial: a multi-level intervention to improve blood pressure control in hypertensive blacks. *Circ Cardiovasc Qual Outcomes.* 2009;2(3):249-256.
41. ALLHAT Officers and Coordinators for the ALLHAT Collaborative Research Group. Major outcomes in high-risk hypertensive patients randomized to angiotensin-converting enzyme inhibitor or calcium channel blocker vs diuretic: The Antihypertensive and Lipid-Lowering Treatment to Prevent Heart Attack Trial (ALLHAT). *JAMA.* 2002;288(23):2981-2997.
42. Armstrong C. JNC8 Guidelines for the Management of Hypertension in Adults. *American family physician.* 2014;90(7):503-504.
43. Wright Jr JT, Fine LJ, Lackland DT, Ogedegbe G, Dennison Himmelfarb CR. Evidence Supporting a Systolic Blood Pressure Goal of Less Than 150 mm Hg in Patients Aged 60 Years or Older: The Minority View. *Ann Intern Med.* 2014.
44. Tullos A. The Black Belt. *Southern Spaces.* 2004.
45. Shea CM, Jacobs SR, Esserman DA, Bruce K, Weiner BJ. Organizational readiness for implementing change: a psychometric assessment of a new measure. *Implementation science : IS.* 2014;9:7.
46. Bandura A. *Social Foundations of Thought and Action: A Social Cognitive Theory.* Englewood Cliffs: Prentice Hall; 1986.
47. Rogers EA, Hessler DM, Bodenheimer TS, Ghorob A, Vittinghoff E, Thom DH. Diabetes peer coaching: do "better patients" make better coaches? *Diabetes Educ.* 2014;40(1):107-115.

48. Lewis LM, Ogedegbe C, Ogedegbe G. Enhancing adherence of antihypertensive regimens in hypertensive African-Americans: current and future prospects. *Expert review of cardiovascular therapy.* 2012;10(11):1375-1380.
49. Lewis LM, Schoenthaler AM, Ogedegbe G. Patient factors, but not provider and health care system factors, predict medication adherence in hypertensive black men. *Journal of clinical hypertension.* 2012;14(4):250-255.
50. Adeseun GA, Bonney CC, Rosas SE. Health literacy associated with blood pressure but not other cardiovascular disease risk factors among dialysis patients. *American journal of hypertension.* 2012;25(3):348-353.
51. Gazmararian JA, Kripalani S, Miller MJ, Echt KV, Ren J, Rask K. Factors associated with medication refill adherence in cardiovascular-related diseases: a focus on health literacy. *Journal of general internal medicine.* 2006;21(12):1215-1221.
52. Kimbro LB, Steers WN, Mangione CM, Duru OK, Ettner SL. The Association of Depression and the Cardiovascular Risk Factors of Blood Pressure, HbA1c, and Body Mass Index among Patients with Diabetes: Results from the Translating Research into Action for Diabetes Study. *International journal of endocrinology.* 2012;2012:747460.
53. Donabedian A. Chapter One: Defining and Measuring the Quality of Health. In: Wenzel R, ed. *Assessing Quality Health Care: Perspectives of Clinicians*: Williams and Wilkins; 1992.
54. Donabedian A. *Evaluating the quality of medical care.* Vol 44: Millbank Memorial Fund; 1966.
55. Donabedian A. Promoting quality through evaluating the process of patient care. *Medical Care.* 1968;6(May-June):181-202.
56. Wagner EH. Chronic disease management: what will it take to improve care for chronic illness? *Eff Clin Pract.* 1998;1(1):2-4.
57. Mezirow J. *Transformative Dimensions of Adult Learning.* San Fransisco, CA: Jossey-Bass; 1991.
58. Gaglio B, Phillips SM, Heurtin-Roberts S, Sanchez MA, Glasgow RE. How pragmatic is it? Lessons learned using PRECIS and RE-AIM for determining pragmatic characteristics of research. *Implement Sci.* 2014;9:96.
59. Gaglio B, Shoup JA, Glasgow RE. The RE-AIM framework: a systematic review of use over time. *Am J Public Health.* 2013;103(6):e38-46.
60. Glasgow RE, Askew S, Purcell P, et al. Use of RE-AIM to Address Health Inequities: Application in a low-income community health center based weight loss and hypertension self-management program. *Transl Behav Med.* 2013;3(2):200-210.
61. Schoen MJ, Tipton EF, Houston TK, et al. Characteristics that predict physician participation in a Web-based CME activity: the MI-Plus study. *J Contin Educ Health Prof.* 2009;29(4):246-253.
62. Crenshaw K, Curry W, Salanitro AH, et al. Is physician engagement with Web-based CME associated with patients' baseline hemoglobin A1c levels? The Rural Diabetes Online Care study. *Acad Med.* 2010;85(9):1511-1517.
63. Estrada CA, Safford MM, Salanitro AH, et al. A web-based diabetes intervention for physician: a cluster-randomized effectiveness trial. *Int J Qual Health Care.* 2011;23(6):682-689.
64. Feldman RD, Zou GY, Vandervoort MK, Wong CJ, Nelson SA, Feagan BG. A simplified approach to the treatment of uncomplicated hypertension: a cluster randomized, controlled trial. *Hypertension.* 2009;53(4):646-653.
65. Santschi V, Chiolero A, Colosimo AL, et al. Improving blood pressure control through pharmacist interventions: a meta-analysis of randomized controlled trials. *Journal of the American Heart Association.* 2014;3(2):e000718.
66. Berlowitz DR, Ash AS, Hickey EC, et al. Inadequate management of blood pressure in a hypertensive population. *New England Journal of Medicine.* 1998;339(27):1957-1963.
67. Safford MM, Shewchuk R, Qu H, et al. Reasons for not intensifying medications: differentiating "clinical inertia" from appropriate care. *J Gen Intern Med.* 2007;22(12):1648-1655.
68. Flack JM, Sica DA, Bakris G, et al. Management of high blood pressure in Blacks: an update of the International Society on Hypertension in Blacks consensus statement. *Hypertension.* 2010;56(5):780-800.
69. Group SR, Wright JT, Jr., Williamson JD, et al. A Randomized Trial of Intensive versus Standard Blood-Pressure Control. *N Engl J Med.* 2015;373(22):2103-2116.
70. Chodosh J, Morton SC, Mojica W, et al. Meta-analysis: chronic disease self-management programs for older adults. *Annals of internal medicine.* 2005;143(6):427-438.

71. Warsi A, Wang PS, LaValley MP, Avorn J, Solomon DH. Self-management education programs in chronic disease: a systematic review and methodological critique of the literature. *Archives of internal medicine.* 2004;164(15):1641-1649.
72. Barlow J, Wright C, Sheasby J, Turner A, Hainsworth J. Self-management approaches for people with chronic conditions: a review. *Patient education and counseling.* 2002;48(2):177-187.
73. Jilcott SB, Keyserling TC, Samuel-Hodge CD, et al. Linking clinical care to community resources for cardiovascular disease prevention: the North Carolina Enhanced WISEWOMAN project. *Journal of women's health.* 2006;15(5):569-583.
74. McGuirt JT, Jilcott SB, Vu MB, Keyserling TC. Conducting community audits to evaluate community resources for healthful lifestyle behaviors: an illustration from rural eastern North Carolina. *Preventing chronic disease.* 2011;8(6):A149.
75. CDC. Self-Measured Blood Pressure Monitoring: Action Steps for Public Health Practitioners. Atlanta, GA: Centers for Disease Control and Prevention, US Department of Health and Human Serviceds; 2013.
76. Yoon PW, Gillespie CD, George MG, Wall HK, Centers for Disease C, Prevention. Control of hypertension among adults--National Health and Nutrition Examination Survey, United States, 2005-2008. *MMWR. Morbidity and mortality weekly report.* 2012;61 Suppl:19-25.
77. Mittman BS. Creating the evidence base for quality improvement collaboratives. *Annals of internal medicine.* 2004;140(11):897-901.
78. J OV, Bate P, Cleary P, et al. Quality collaboratives: lessons from research. *Quality & safety in health care.* 2002;11(4):345-351.
79. Cretin S, Shortell SM, Keeler EB. An evaluation of collaborative interventions to improve chronic illness care. Framework and study design. *Evaluation review.* 2004;28(1):28-51.
80. Young PC, Glade GB, Stoddard GJ, Norlin C. Evaluation of a learning collaborative to improve the delivery of preventive services by pediatric practices. *Pediatrics.* 2006;117(5):1469-1476.
81. Pearson ML, Wu S, Schaefer J, et al. Assessing the implementation of the chronic care model in quality improvement collaboratives. *Health services research.* 2005;40(4):978-996.
82. Roberts RO, Bergstralh EJ, Schmidt L, Jacobsen SJ. Comparison of self-reported and medical record health care utilization measures. *Journal of clinical epidemiology.* 1996;49(9):989-995.
83. Beckles GL, Williamson DF, Brown AF, et al. Agreement between self-reports and medical records was only fair in a cross-sectional study of performance of annual eye examinations among adults with diabetes in managed care. *Med Care.* 2007;45(9):876-883.
84. Mangione CM, Gerzoff RB, Williamson DF, et al. The association between quality of care and the intensity of diabetes disease management programs. *Ann Intern Med.* 2006;145(2):107-116.
85. Ackermann RT, Thompson TJ, Selby JV, et al. Is the number of documented diabetes process-of-care indicators associated with cardiometabolic risk factor levels, patient satisfaction, or self-rated quality of diabetes care? The Translating Research into Action for Diabetes (TRIAD) study. *Diabetes Care.* 2006;29(9):2108-2113.
86. Kim C, Williamson DF, Mangione CM, et al. Managed care organization and the quality of diabetes care: the Translating Research Into Action for Diabetes (TRIAD) study. *Diabetes Care.* 2004;27(7):1529-1534.
87. Nicholson LM, Schwirian PM, Klein EG, et al. Recruitment and retention strategies in longitudinal clinical studies with low-income populations. *Contemporary clinical trials.* 2011;32(3):353-362.
88. Gross D, Julion W, Fogg L. What Motivates Participation and Dropout Among Low-Income Urban Families of Color in a Prevention Intervention? *Family Relations.* 2001;50(3):246-254.
89. Mase R, Halasyamani L, Choi H, Heisler M. Who Signs Up for and Engages in a Peer Support Heart Failure Self-management Intervention. *The Journal of cardiovascular nursing.* 2014.
90. Rosenberger EL, Goff DC, Jr., Davis CC, Blackwell CS, Bertoni AG. Control of blood pressure in North Carolina primary care: baseline data from the GLAD Heart Trial. *N C Med J.* 2008;69(6):441-446.
91. Brookes ST, Whitely E, Egger M, Smith GD, Mulheran PA, Peters TJ. Subgroup analyses in randomized trials: risks of subgroup-specific analyses; power and sample size for the interaction test. *J Clin Epidemiol.* 2004;57(3):229-236.
92. Charlson ME, Pompei P, Ales KL, McKenzie CR. A new method of classifying prognostic comorbidity in longitudinal studies: development and validation. *Journal of Chronic Diseases.* 1987;40(5):373-383.

93. Halladay JR, DeWalt DA, Wise A, et al. More extensive implementation of the chronic care model is associated with better lipid control in diabetes. *J Am Board Fam Med.* 2014;27(1):34-41.
94. Fisher EB, Ayala GX, Ibarra L, et al. Contributions of Peer Support to Health, Health Care, and Prevention: Papers from Peers for Progress. *Ann Fam Med.* 2015;13 Suppl 1:S2-8.
95. Simmons D, Bunn C, Nakwagala F, et al. Challenges in the Ethical Review of Peer Support Interventions. *Ann Fam Med.* 2015;13 Suppl 1:S79-86.
96. Carey, Robert M.,Paul K. Whelton, et al. "Prevention, Detection, Evaluation, and Management of High Blood Pressure in Adults: Synopsis of the 2017 American College of Cardiology/American Heart Association Hypertension Guideline." Annals of Internal Medicine, vol. 168, no. 5, 2018, p. 351., doi:10.7326/m17-3203.
