# Supplementary material for: Practice Facilitation and Peer Coaching for Uncontrolled Hypertension Among Black Individuals: A Randomized Clinical Trial
Source: JAMA Intern Med. 2024 Mar 18;184(5):538–46. doi: 10.1001/jamainternmed.2024.0047 (PMC10949149; doi:10.1001/jamainternmed.2024.0047)
Supplement: Supplement 2. — eMethods 1. Details on the decision to reduce the trial’s sample size eMethods 2. Additional details on loss to follow-up eMethods 3. Examples of quality improvement activities eTable 1. Practice characteristics eTable 2. Perceived usefulness of Peer Coaches eTable 3. Practice Champions’ ratings of Practice Facilitators [file jamainternmed-e240047-s002.pdf]

Supplemental Online Content

Safford MM, Cummings DM, Halladay J, et al. Practice facilitation and peer coaching for hypertension among African American patients: a randomized clinical trial. *JAMA Intern Med*. Published March 18, 2024. doi:10.1001/jamainternmed.2024.0047

eMethods 1. Details on the decision to reduce the trial's sample size
eMethods 2. Additional details on loss to follow-up
eMethods 3. Examples of quality improvement activities
eTable 1. Practice characteristics
eTable 2. Perceived usefulness of peer coaches
eTable 3. Practice champions' ratings of practice facilitators

This supplemental material has been provided by the authors to give readers additional information about their work.

eMethods 1. Details on the decision to reduce the trial's sample size

This trial was a real-world study, with real-world unanticipated events that led to delays in accrual. The primary care practices we engaged experienced natural disasters including hurricanes, winter storms, floods, and the COVID-19 pandemic, all of which incurred delays and extra costs. Furthermore, in 2019, a research assistant admitted that she had falsified data, rendering the data she collected unusable. This discovery led to a reanalysis of the study's power to test the main hypothesis that now included actual intraclass correlation coefficients (rather than the pretrial estimates). The reanalysis revealed that a smaller sample size would still meet the study's main objectives. The Data and Safety Monitoring Board in December 2019 therefore approved the proposal to proceed with a smaller sample size of approximately 1600 participants and 69 practices. This new sample size provided at least 85% power to detect the study's original design goals when analyzed at the individual patient level. The original plans to analyze data at the practice level were abandoned due to insufficient number of practices. See also Safford MM, Cummings D, Halladay J, et al. The design and rationale of a multicenter real-world trial: The Southeastern Collaboration to Improve Blood Pressure Control in the US Black Belt – Addressing the Triple Threat. *Contemporary Clinical Trials* June 129 (2023) 107183.

eMethods 2. Additional details on loss to follow-up

Two practices withdrew, one in the peer coaching arm and one in the enhanced usual care arm. The latter had 4 participants at the time of withdrawal who continued to be followed and grouped with a nearby practice; thus, this practice's participants were not lost to follow-up although the practice closed. Practices lost to follow-up were missing both 6 and 12-month follow-up data.

eMethods 3. Examples of quality improvement activities

- Training and mutual reminder system to assess blood pressure following national guidelines.
- Estimating practice level blood pressure control.
- Create a hypertension registry.
- Use monthly practice level blood pressure control estimates to monitor progress toward population level blood pressure control.
- Implement home blood pressure monitoring.
- Implement a protocol to allow medical assistants to call patients in the hypertension registry who miss their appointments.
- Use teach back when providing education to hypertension patients.
- Provide blood pressure checks without appointment or co-pay.
- Implement a protocol for appointment reminders for hypertension patients with reminder to bring medications and their home blood pressure readings on the day of the visit.
- Refer patients to the Patient Activated Learning System to answer questions about medications.
- Implement a workflow to highlight abnormal blood pressure readings for providers.
- Implement a workflow to assure medication reconciliation at each visit.
- Use templates to capture treatment goals, barriers to adherence.

© 2024 Safford MM et al. *JAMA Internal Medicine*.

eTable 1. Practice characteristics

Characteristic	Enhanced Usual Care	Practice Facilitation	Peer Coaching	Peer Coaching plus Practice Facilitation
Number of practices	18	16	19	16
FQHC or community clinic, n (%)	8 (44)	9 (56)	9 (47)	7 (44)
Mean years in operation (range)	23 (1-89)	18 (3-39)	20 (3-43)	14 (1-42)
Staffing, mean n ±SD				
FTE full-time providers[a]	7 ±14	3 ±3	3 ±2	4 ±5
FTE full-time staff[b]	20 ±29	12 ±14	9 ±7	10 ±6
Insurance, %				
Medicare	26	25	22	20
Medicaid	20	23	20	23
Medicare plus Medicaid	10	8	9	9
Commercial	25	16	27	26
None	16	27	20	20
Visits per year, mean	18,651	11,820	8,168	9,167
Patient characteristics, %				
65 years of age or older	29	26	29	30
African Americans	56	59	56	56
PCMH recognition, n (%)	5 (28)	6 (38)	5 (28)	4 (27)

[a]Physicians, nurse practitioners, physician assistants, psychologists.
[b]Pharmacists, nurses, dietitians, medical assistants, laboratory staff, health coaches, social workers, administrative staff.
Abbreviations: FQHC = Federally Qualified Health Center; FTE = full time equivalent; PCMH = Patient Centered Medical Home. SD = standard deviation.

© 2024 Safford MM et al. *JAMA Internal Medicine*.

eTable 2. Perceived usefulness of peer coaches

Participant opinion about peer coach activity	N (%)
Thought teaching how to use a home blood pressure monitor and how to measure their blood pressure was very helpful or helpful	403 (93)
Thought that peer coach reminding them to take their blood pressure medicines was very helpful or helpful	411 (96)
Thought that peer coach reminding them to take their other medicines was very helpful or helpful	390 (91)
Thought that peer coach reminding them to follow a healthy diet was very helpful or helpful	415 (96)
Thought that peer coach giving them information about healthy eating was very helpful or helpful	409 (95)
Thought that peer coach reminding them to be physically active was very helpful or helpful	415 (96)
Thought that peer coach talking to them about issues that may affect their ability to take care of their blood pressure was very helpful or helpful	409 (95)
Thought that peer coach talking to them about issues that may affect their ability to take care of their other health conditions was very helpful or helpful	393 (91)
Thought that peer coach talking to them about what happened at their last doctor visit was very helpful or helpful	389 (90)
Thought that peer coach reminding them to keep their next doctor visit was very helpful or helpful	404 (94)
Thought that peer coach helping them to prepare questions to discuss with their doctor was very helpful or helpful	372 (87)
Thought that peer coach helping them find social services and community resources they may need was very helpful or helpful	353 (82)
Thought that peer coach helping them practice what they want to say to their doctor, when it may be something uncomfortable, such as cost or medication side effects was very helpful or helpful	357 (83)
Thought that peer coach met almost all or most of their needs	398 (92)
Thought their peer coach's honesty was excellent/very good	389 (90)
Thought the respect that their peer coach showed was excellent/very good	394 (91)
Thought how caring and concerned their peer coach was was excellent/very good	389 (90)
Thought the time that their peer coach took with them was excellent/very good	389 (90)
Thought how well their peer coach listens to them was excellent or very good	396 (92)
Thought their peer coach's dependability was excellent or very good	389 (90)
Thought their peer coach's attitude in general was excellent or very good	394 (91)
Strongly agreed or agreed that it's a good idea for other people with high blood pressure to have a peer coach	417 (97)
Liked their peer coach	428 (99)
Thought that peer coach liked them	425 (99)
Felt that their peer coach is like them	403 (94)
Rated quality of service they received from peer coach as excellent/good	420 (97)

© 2024 Safford MM et al. *JAMA Internal Medicine*.

eTable 3. Practice champions' ratings of practice facilitators

Activity	N (%)
Receiving information on the prevalence of hypertension and/or uncontrolled hypertension among your patients was very or somewhat helpful	30 (91)
Receiving training on evidence-based practice guidelines for hypertension, such as BP measurement techniques and BP treatment resources was very or somewhat helpful	31 (94)
Receiving assistance in customizing workflows and procedures for management of patients with hypertension was very or somewhat helpful	31 (94)
Identifying performance measures to track your improvement in managing the care of your patients with hypertension was very or somewhat helpful	30 (91)
Having a practice facilitator on site to work with you and your staff was very or somewhat helpful	32 (97)
Having a practice facilitator work with you and your staff via web/telephone was very or somewhat helpful	25 (76)
Receiving ongoing hypertension educational resources (e.g., websites, videos, flyers, blood pressure treatment algorithms) for patients and staff was very or somewhat helpful	30 (91)
Receiving information about local/community-level resources and other supports for patients to help them care for themselves between office visits was very or somewhat helpful	30 (91)

Note: At one practice, two individuals served as Practice Champions, thus these 33 Practice Champions responded on behalf of 32 practices.

© 2024 Safford MM et al. *JAMA Internal Medicine*.
