# Supplementary material for: Practice Facilitation and Peer Coaching for Uncontrolled Hypertension Among Black Individuals: A Randomized Clinical Trial
Source: JAMA Intern Med. 2024 Mar 18;184(5):538–46. doi: 10.1001/jamainternmed.2024.0047 (PMC10949149; doi:10.1001/jamainternmed.2024.0047)
Supplement: Supplement 3. — Data Sharing Statement [file jamainternmed-e240047-s003.pdf]

Data Sharing Statement

Safford. Practice Facilitation and Peer Coaching for Hypertension Among African American Patients. *JAMA Intern Med*. Published March 18, 2024. doi:10.1001/jamainternmed.2024.0047

Data
Data available: Yes
Data types: Other (please specify)
Additional Information: Available from corresponding author upon request.
How to access data: Available from corresponding author (mms9024@med.cornell.edu) upon request.
When available: beginning date: 11-01-2023

Supporting Documents
Document types: None

Additional Information
Who can access the data: Researcher whose proposed use of the data has been approved.
Types of analyses: For additional research.
Mechanisms of data availability: After approval of a proposal.
